# Supplementary material for: Bioinformatic Analysis of Secondary Metabolite Biosynthetic Potential in Pathogenic Fusarium
Source: J Fungi (Basel). 2023 Aug 15;9(8):850. doi: 10.3390/jof9080850 (PMC10455296; doi:10.3390/jof9080850)
Supplement: Supplementary file 1 [file jof-09-00850-s001.zip › jof-2530492-supplementary.pdf]

Bioinformatic Analysis of Secondary Metabolite Biosynthetic Potential in Pathogenic *Fusarium*

Chao Lin [1], Xi-long Feng [1], Yu Liu [1], Zhao-chen Li [1], Xiu-Zhang Li [2] and Jianzhao Qi [1,*]

1 Shaanxi Key Laboratory of Natural Products & Chemical Biology, College of Chemistry & Pharmacy, North-west A&F University, Yangling, Xianyang 712100, China
2 State Key Laboratory of Plateau Ecology and Agriculture, Qinghai Academy of Animal and Veterinary Sciences, Qinghai University, Xining 810016, China
* Correspondence: qjz@nwafu.edu.cn

Supplementary Table. ... 3
Table S1: The 35 pathogenic *Fusarium* species used in this study. ... 3
Table S2: BGC statistics based on antiSMASH predictions. ... 4
Table S3: The 16 identified BGCs for GCF network establishment. ... 5
Table S4: Sequence identities (%) of Ffsc4 and its homologues. ... 6
Table S5: Sequence identities (%) of Ffsc6 and its homologues. ... 7
Table S6: Sequence identities (%) of STC5 and homologues. ... 8
Table S7: Sequence identities (%) of STC3 and homologues. ... 9
Table S8: Sequence identities (%) of CLM1 and homologues. ... 10
Table S9: Sequence identities (%) of TRI5 and homologues. ... 11
Table S10: Sequence identities (%) of FlvE and homologues. ... 12
Table S11: Sequence identities (%) of DpfgD and homologues. ... 13
Table S12: Sequence identities (%) of GGS and homologues. ... 14
Table S13: Sequence identities (%) of CPS/KS and homologues. ... 16
Table S14: Sequence identities (%) of DpfgB and homologues. ... 17
Table S15: Sequence identities (%) of FgMS, FoFs and homologues. ... 18
Table S16: Sequence identities (%) of ERG7 and homologues. ... 19
Table S17: Sequence identities (%) of CarRA and homologues. ... 20
Table S18: Sequence identities (%) of DMATS1 and homologues. ... 21
Table S19: Sequence identities (%) of BEA1 and homologues. ... 22

Supplementary Figures. ... 23
Figure S1: GCF network of 1733 predicted biosyntheic gene cluster (BGC) from 35 pathogenic *Fusarium* species caculated by BiG-SCAPE pipeline and visualised with Cytoscape. ... 23
Figure S2: Cluster analysis of terpenoid synthase and their homologues based on phylogenetic tree. ... 24
Figure S3: Comparison of *Flv*BGCs from different species. ... 25
Figure S4: Phylogenetic tree-based cluster analysis of GGPP-related enzymes. ... 26
Figure S5: Comparison of *dpfg*BGCs from different pathogenic *Fusarium* species. ... 27
Figure S6: The predicted three-dimensional structure of protein of FgMS (A), FGSG_01738 (B), FGRMN_7913 (C), FPCIR_12113 (D), FPANT_13888 (E) and FoFs(F) predicted by Alphafold. ... 28
Figure S7: Phylogenetic tree-based cluster analysis of CarRA and its homologues. ... 29
Figure S8: Domain comparison of NPS1 and its homologues(A), comparison of the BGCs containing *nps1* from different species(B). ... 30
Figure S9: Domain comparison of NPS2 and its homologues. ... 31
Figure S10: Domain comparison of NPS6 and its homologues. ... 32
Figure S11: Domain comparison of SidE and its homologues. ... 33
Figure S12: Domain comparison of SidC and its homologues. ... 34
Figure S13: Domain comparison of ESYN1 and its homologues. ... 35
Figure S14: Domain comparison of PesF and its homologues. ... 36
Figure S15: Domain comparison of HTS1 and its homologues. ... 37
Figure S16: Comparison of *Bea*BGCs from different pathogenic fungi. ... 38
Figure S17: Comparative analysis of AclP and its homologues. Comparison of the amino acid sequence identity of AclP and its homologues(A), comparison of the structural domains of AclP and its homologues(B), structure of cyclo-(L-Phe-L-Phe)(C). ... 39
Figure S18: Comparison of *Acl*BGCs from different species. ... 40
Figure S19: Comparison of the BGCs containing *nrps4* from different *Fusarium* species. ... 41
Figure S20: Comparison of the BGCs containing *nrps5* from different *Fusarium* species. ... 42
Figure S21: Comparison of the BGCs containing *nrps*7 and PKS6 from different *Fusarium* species. ... 43
Figure S22: Structures of sansalvamide (A), comparison of the amino acid sequence identity of NRPS30 and its homologues (B), domain comparison of NRPS30 and its homologues (C), comparison of the BGC for aurofusarin and its similar BGCs (D). ... 44

Figure S23: Comparison of the amino acid sequence identity of Chry1 and its homologues (A), domain comparison of Chry1 and its homologues (B), comparison of *chry*BGCs from different *Fusarium* species. 45
Figure S24: Comparison of the amino acid sequence identity of GRA1 and its homologues (A), domain comparison of GRA1 and its homologues (B), comparison of the BGC for Gramillin A, Gramillin B and its similar BGCs (C), structure of Gramillin A and Gramillin B (D). 46
Figure S25: Domain comparison of apf1 and its homologues (A), comparison of *apf*BGCs from different *Fusarium* species (B). 47
Figure S26: Domain comparison of FrbI and its homologues (A), domain comparison of PKS-NRPS1 and its homologues (B), domain comparison of FUS1 and its homologues (C). 48
Figure S27: Phylogenetic tree-based cluster analysis of PKS-related enzymes. 49
Figure S28: Comparison of *gpy*BGCs from different *Fusarium* species. 50
Figure S29: Comparison of *fsr*BGCs from different *Fusarium* species. 51
Figure S30: Comparison of *FUB*BGCs from different *Fusarium* species. 52
Figure S31: Comparison of *bik*BGCs from different *Fusarium* species. 53
Figure S32: Comparison of *FPY*BGCs from different *Fusarium* species. 54
Figure S33: Comparison of *fog*BGCs from different *Fusarium* species. 55
Figure S34: Comparison of the amino acid sequence identity of SdnO and its homologues (A), domain comparison of SdnO and its homologues (B). 56
Figure S35: Comparison of *DEP*BGCs from different *Fusarium* species. 57
Figure S37: Domain comparison of PKS40 and its homologues. 59
Figure S39: Comparison of the amino acid sequence identity of Alt5 and its homologues (A), domain comparison of Alt5 and its homologues (B). 61
Figure S40: Domain comparison of DpfgA and its homologues. 62
Figure S41: Domain comparison of FSL1 and its homologues (A), comparison of *FSL*BGCs from different *Fusarium* species (B). 63
Figure S42: Domain comparison of Bet1 and its homologues (A), comparison of *bet*BGCs from different species (B). 64
Figure S43: Domain comparison of G433 and its homologues (A), comparison of the BGC for G433 and its similar BGCs (B). 65
Figure S44: Comparison of the amino acid sequence identity of Alt5 and its homologues (A), domain comparison of FUM1 and its homologues (B), comparison of *FUM*BGCs from different *Fusarium* species (C). 66
Figure S45: Domain comparison of ZEA1 and its homologues (A), domain comparison of ZEA2 and its homologues (B). 67
Figure S46: Domain comparison of PkhA and its homologues (A), domain comparison of PkhB and its homologues (B). 68
Figure S47: Comparison of *luc*BGCs from different *Fusarium* species. 69
Figure S48: Amino acid sequence identity comparison (A) and domain comparison (B) of newly discoveried PKS-NRPSs in 35 pathogenic *Fusarium* species. 70
Figure S49: Comparison of *smb*BGCs from different *Fusarium* species. 71
Figure S50: Comparison of *fsa*BGCs and *PKS-NRPS*BGCs from different *Fusarium* species. 72
Figure S51: Comparison of *icc*BGCs from different *Fusarium* species. 73
Figure S52: Comparison of the amino acid sequence identity of FsdS and its homologues (A), domain comparison of FsdS and its homologues (B), comparison of *fsd*BGCs from different *Fusarium* species (C). 74
Figure S53: Domain comparison of ACE1 and its homologues (A), comparison of *ACE*BGCs from different species (B). 75
Figure S54: Domain comparison of ThnA and its homologues (A), comparison of *thn*BGCs from different species (B). 76
Figure S55: Domain comparison of CghG and its homologues. 77
Figure S56: Domain comparison of LCI18_013989 and jgi.p_Fustri_620762. 78
Figure S57: Cluster analysis of NRPS-like sequences based on phylogenetic tree. 79

References. 80

Supplementary Table.

Table S1: The 35 pathogenic *Fusarium* species used in this study.

Species	Pathogenicity	Accession No.	Reference
Fusarium oxysporum	Wilt diseases	GCF_000271745.1	[1]
Fusarium fujikuroi	Bakanae disease	GCF_900079805.1	[2]
Fusarium solani-melongenae	*Fusarium* Root and Stem Rot in *Sweet potatoes*	GCA_023101225.1	[3]
Fusarium vanettenii	rot disease	GCF_000151355.1	[4]
Fusarium odoratissimum	*Fusarium* wilt of banana	GCF_000260195.1	[5]
Fusarium proliferatum	Vascular Wilt on Cowpea (*Vigna unguiculata*) in Brazil	GCF_900067095.1	[6]
Fusarium graminearum	*Fusarium* head blight in small-grain cereals	GCF_000240135.3	[7]
Fusarium pseudograminearum	wheat crown rot disease	GCF_000303195.2	[8]
Fusarium mangiferae	mango malformation disease (MMD)	GCF_900044065.1	[9]
Fusarium culmorum	root rot and head blight in wheat.	GCA_016952355.1	[10]
Fusarium solani	Root Rot and Stem Canker on Storage Roots of Sweet Potato	GCF_020744495.1	[11]
Fusarium redolens	American Ginseng Root Rot	GCF_020744475.1	[12]
Fusarium flagelliforme	Maize Leaf Blight	GCF_020744385.1	[13]
Fusarium musae	post-harvest disease in bananas and systemic and superficial infection in humans.	GCF_019915245.1	[14]
Fusarium falciforme	Leaf Blight in Acacia mangium	GCF_026873545.1	[15]
Fusarium venenatum	foot and root rot of wheat (*Triticum aestivum*)	GCF_900007375.1	[16]
Fusarium tricinctum	*Fusarium* head blight (FHB) and root rot	GCA_020744515.1	[17]
Fusarium poae	*Fusarium* Head Blight of Wheat	GCF_019609905.1	[18]
Fusarium keratoplasticum	associated with human infection	GCF_025433545.1	[19]
Fusarium graminum	*Fusarium* head blight (FHB) of small grain cereals	GCA_013266165.1	[20]
Fusarium napiforme	disseminated fusariosis	GCA_013396005.1	[21]
Fusarium mexicanum	agent of mango and big-leaf mahogany malformation in Mexico	GCA_013396015.1	[22]
Fusarium decemcellulare	Inflorescence Wilt and Vascular and Flower Necrosis of Rambutan (Nephelium lappaceum), Longan (Dimocarpus longan), and Mango (Mangifera indica)	GCA_013266205.1	[23]
Fusarium acutatum	permanent wilting of chickpea cuttings and killed cells	GCA_012932015.1	[24]
Fusarium subglutinans	Maize pathogen	GCF_013396075.1	[25]
Fusarium verticillioides	kernel and ear rot of maize	GCF_000149555.1	[26]
Fusarium avenaceum	Wheat pathogen		[27]
Fusarium globosum	Chlorosis and necrosis of wheat leaves and maize stems	GCA_013396165.1	[28]
Fusarium mundagurra	producer of fumonisins	GCA_013396205.1	[29]
Fusarium pseudocircinatum	Mango Malformation Disease	GCA_013396035.1	[30]
Fusarium denticulatum	chlorotic leaf distortion with deformation of young leaves and stunted vines.	GCA_013396175.1	[31]
Fusarium pseudoanthophilum	were capable of infecting vegetables such as tomatoes, bell and cayenne peppers,	GCA_013395995.1	[32]
Fusarium anthophilum	produced at least one of the analyzed mycotoxins	GCA_013364935.1	[33]
Fusarium sarcochroum	dry root rot, crown, trunk or twig canker or twig dieback of citrus trees	GCA_013266185.1	[34]
Fusarium moniliforme	ear rot and stalk rot of corn and infection of corn kernels		[35]

Table S2: BGC statistics based on antiSMASH predictions.

Species	BGC Type							Total
	NRPS	PKS	RiPP	Terpene	Hybird	Indole	Other	
Fusarium oxysporum	19	8	2	9	6	2	1	47
Fusarium fujikuroi	16	8	4	12	9	1	2	52
Fusarium solani-melongenae	15	11	1	6	4	1	1	39
Fusarium vanettenii	14	12	1	6	3	1	1	38
Fusarium odoratissimum	15	8	2	11	6	2	1	45
Fusarium proliferatum	22	11	4	13	8	2	1	61
Fusarium graminearum	15	7	4	11	10	0	1	48
Fusarium pseudograminearum	14	5	2	10	9	0	0	40
Fusarium mangiferae	17	7	3	13	9	3	1	53
Fusarium culmorum	14	7	2	12	5	0	1	41
Fusarium solani	14	11	1	5	7	1	1	40
Fusarium redolens	18	9	4	11	7	2	0	51
Fusarium flagelliforme	18	7	2	9	6	1	1	44
Fusarium musae	18	7	2	11	5	2	1	46
Fusarium falciforme	13	13	1	5	5	1	1	39
Fusarium venenatum	14	5	5	12	8	0	0	44
Fusarium tricinctum	20	9	6	11	10	3	1	60
Fusarium poae	15	9	4	14	5	0	1	48
Fusarium keratoplasticum	11	11	1	6	7	1	1	38
Fusarium graminum	16	12	6	9	5	2	1	51
Fusarium napiforme	17	13	4	10	7	3	1	55
Fusarium mexicanum	18	14	2	11	11	3	1	60
Fusarium decemcellulare	22	17	3	12	6	3	1	64
Fusarium acutatum	18	11	3	11	8	2	1	54
Fusarium subglutinans	19	13	3	11	6	3	1	56
Fusarium verticillioides	17	12	1	6	7	2	1	46
Fusarium avenaceum	19	12	5	12	9	2	2	61
Fusarium globosum	22	14	3	13	7	2	1	62
Fusarium mundagurra	21	15	3	10	5	2	1	57
Fusarium pseudocircinatum	16	14	5	11	8	1	1	56
Fusarium denticulatum	15	15	2	13	11	1	1	58
Fusarium pseudoanthophilum	19	13	5	12	6	2	1	58
Fusarium anthophilum	23	15	5	13	7	1	1	65
Fusarium sarcochroum	16	11	10	9	7	2	0	55
Fusarium moniliforme	17	12	1	6	7	2	1	46

Table S3: The 16 identified BGCs for GCF network establishment.

Type	GCF	Identified BGC	Representative Compound	Reference
PKS I	fsr_GCF	*fsr*BGC	oxyjavanicin	[36]
	BIK_GCF	*BIK*BGC	bikaverin	[37]
	ACTT/PKS19<br>_GCF	*ACTT*/*PKS19*BGC	fujikurin A-D<br>ACT-Toxin II	[38, 39]
	DEP_GCF	*DEP*BGC	depudecin	[40]
	alt_GCF	*alt*BGC	alternapyrone<br>alternapyrone B-F	[41, 42]
Terpene	SQS1_GCF	*SQS1*BGC	squalestatin S1	[43]
	Ffsc_GCF	*Ffsc*BGC	koraiol	[44]
	GA_GCF	*GA*_BGC	gibberellin	[45]
	Tri_GCF	*tri*BGC	deoxynivalenol<br>nivalenol<br>trichodiene-11-one	[46-48]
NRPS	chry_GCF	*chry*BGC	chrysogine	[49]
	san_GCF	*san*BGC	sansaivamide	[50]
	aba_GCF	*aba*BGC	AbT1<br>KK-1	[51, 52]
	APS_GCF	*APS*BGC	apicidin	[53]
PKS-<br>NRPS_<br>Hybrid	FSL_GCF	*FSL*BGC	fusarielin H	[54]
	ZEA_GCF	*ZEA*BGC	zearalenone	[55]
Other	has_GCF	*has*BGC	hexadehydroastechrome	[56]
	fsd_GCF	*fsd*BGC	fusaridione A	[57]

Table S4: Sequence identities (%) of Ffsc4 and its homologues.

FNAPI_12378	FPANT_5545	J7337_010974	FDENT_14120	FPCIR_8416	FMEXI_13175	FSUBG_8956	FANTH_11676	FACUT_6830	FMUND_8404	FGLOB1_10057	Ffsc4(FFUJ_12585)	FPRO_12247	FMAN_11750	BKA55DRAFT_562495	BKA59DRAFT_435158	jgi.p_Fustri1_103320	FGRMN_1905	FGSG_11327	HYE67_005221	FPSE_05683	FPOAC1_007502	FVRRES_10751	FPOAC1_012967	FPOAC1_013813	FPOAC1_003894	B0J16DRAFT_345894	BKA59DRAFT_497532	FSARC_7662	FDECE_5463
100	99.18	99.4	98.08	98.9	97.53	98.08	97.53	97.81	97.53	96.44	96.71	96.99	97.26	95.89	88.49	87.12	86.07	81.94	81.64	82.47	81.1	81.94	77.47	77.2	83.33	85.36	77.57	83.79	75.35
99.18	100	99.4	97.81	98.63	97.26	97.81	97.26	97.53	97.26	96.16	96.44	96.71	96.99	95.62	88.22	87.4	85.79	82.22	81.92	82.74	81.37	82.22	77.75	77.47	83.33	85.08	77.26	83.79	74.79
99.4	99.4	100	98.51	99.4	97.91	98.51	97.91	98.51	98.21	97.01	97.31	97.31	97.61	96.12	88.06	87.16	85.71	82.73	82.69	83.28	82.09	82.73	78.74	78.44	85.05	85.84	78.23	83.83	79.88
98.08	97.81	98.51	100	98.63	97.26	97.81	97.26	97.53	97.26	96.16	96.44	96.71	96.99	96.16	87.95	86.85	85.52	81.94	81.64	82.47	81.1	81.94	77.47	77.2	82.54	85.36	77.88	83.79	74.79
98.9	98.63	99.4	98.63	100	98.08	98.63	98.08	98.36	98.08	96.99	97.26	97.53	97.81	96.44	88.77	87.4	86.35	82.5	82.19	83.01	81.64	82.5	78.02	77.75	84.13	85.91	78.5	84.34	75.35
97.53	97.26	97.91	97.26	98.08	100	99.18	98.08	96.99	96.71	96.71	96.44	97.26	97.53	95.62	87.67	86.3	85.24	82.5	82.19	82.74	81.64	82.5	78.02	77.75	83.33	85.64	78.82	84.62	75.62
98.08	97.81	98.51	97.81	98.63	99.18	100	98.9	97.53	97.26	97.26	96.99	97.81	98.08	96.16	88.22	86.85	85.79	82.78	82.47	83.29	81.92	82.78	78.3	78.02	84.13	86.19	78.82	84.34	75.62
97.53	97.26	97.91	97.26	98.08	98.08	98.9	100	96.99	96.71	96.99	96.71	97.53	97.81	96.16	87.67	86.3	85.24	82.78	82.47	83.29	81.92	82.78	78.3	78.02	84.13	86.19	78.82	84.34	75.62
97.81	97.53	98.51	97.53	98.36	96.99	97.53	96.99	100	98.08	96.99	96.99	97.53	97.81	96.16	88.22	86.85	85.79	82.78	82.47	83.29	82.19	82.22	77.75	77.47	82.54	85.64	77.57	84.07	75.07
97.53	97.26	98.21	97.26	98.08	96.71	97.26	96.71	98.08	100	96.99	96.99	97.53	97.81	95.89	88.49	86.85	86.07	82.5	82.47	83.01	81.92	82.5	78.57	78.3	84.13	85.91	77.88	84.34	75.62
96.44	96.16	97.01	96.16	96.99	96.71	97.26	96.99	96.99	96.99	100	98.08	99.45	99.18	95.89	87.95	86.58	85.79	82.22	81.92	82.74	81.64	82.22	78.3	78.3	84.13	85.64	77.88	84.07	75.07
96.71	96.44	97.31	96.44	97.26	96.44	96.99	96.71	96.99	96.99	98.08	100	98.63	98.36	96.16	87.95	86.58	86.35	82.5	82.19	83.01	81.92	82.5	78.3	78.3	83.33	85.91	78.5	84.34	75.62
96.99	96.71	97.31	96.71	97.53	97.26	97.81	97.53	97.53	97.53	99.45	98.63	100	99.73	96.44	88.22	87.12	86.35	82.78	82.47	83.29	82.19	82.78	78.3	78.3	83.33	86.19	78.19	84.34	75.35
97.26	96.99	97.61	96.99	97.81	97.53	98.08	97.81	97.81	97.81	99.18	98.36	99.73	100	96.71	88.49	87.4	86.63	83.06	82.74	83.56	82.47	83.06	78.57	78.3	84.13	86.46	78.5	84.62	75.62
95.89	95.62	96.12	96.16	96.44	95.62	96.16	96.16	96.16	95.89	95.89	96.16	96.44	96.71	100	87.12	85.75	84.68	82.78	82.47	83.29	81.92	82.78	78.02	77.75	84.13	86.19	79.13	85.16	75.62
88.49	88.22	88.06	87.95	88.77	87.67	88.22	87.67	88.22	88.49	87.95	87.95	88.22	88.49	87.12	100	96.02	90.03	81.44	81.15	81.97	81.15	81.72	76.5	76.23	83.33	84.89	77.02	83.38	73.2
87.12	87.4	87.16	86.85	87.4	86.3	86.85	86.3	86.85	86.85	86.58	86.58	87.12	87.4	85.75	96.02	100	88.41	81.16	80.87	81.69	81.15	81.44	76.23	75.96	81.75	84.07	76.4	82.56	72.65
86.07	85.79	85.71	85.52	86.35	85.24	85.79	85.24	85.79	86.07	85.79	86.35	86.35	86.63	84.68	90.03	88.41	100	80.83	80.28	81.11	80	81.11	76.67	76.39	83.47	83.57	76.9	80.61	71.91
81.94	82.22	82.73	81.94	82.5	82.5	82.78	82.78	82.78	82.5	82.22	82.5	82.78	83.06	82.78	81.44	81.16	80.83	100	99.45	99.72	97.51	98.34	85.83	85.56	84.3	93.85	78.48	80.83	70.87
81.64	81.92	82.69	81.64	82.19	82.19	82.47	82.47	82.47	82.47	81.92	82.19	82.47	82.74	82.47	81.15	80.87	80.28	99.45	100	99.18	96.99	97.78	85.48	85.21	84.13	93.39	78.19	80.55	70.72
82.47	82.74	83.28	82.47	83.01	82.74	83.29	83.29	83.29	83.01	82.74	83.01	83.29	83.56	83.29	81.97	81.69	81.11	99.72	99.18	100	97.27	98.34	85.75	85.48	84.13	94.21	78.5	81.1	71.55
81.1	81.37	82.09	81.1	81.64	81.64	81.92	81.92	82.19	81.92	81.64	81.92	82.19	82.47	81.92	81.15	81.15	80	97.51	96.99	97.27	100	97.51	84.11	83.84	83.33	93.11	77.88	80.6	70.8
81.94	82.22	82.73	81.94	82.5	82.5	82.78	82.78	82.22	82.5	82.22	82.5	82.78	83.06	82.78	81.72	81.44	81.11	98.34	97.78	98.34	97.51	100	85.83	85.56	85.12	93.85	78.48	81.11	71.15
77.47	77.75	78.74	77.47	78.02	78.02	78.3	78.3	77.75	78.57	78.3	78.3	78.3	78.57	78.02	76.5	76.23	76.67	85.83	85.48	85.75	84.11	85.83	100	99.73	100	85.67	76.64	77.26	69.34
77.2	77.47	78.44	77.2	77.75	77.75	78.02	78.02	77.47	78.3	78.3	78.3	78.3	78.3	77.75	76.23	75.96	76.39	85.56	85.21	85.48	83.84	85.56	99.73	100	99.21	85.4	76.32	76.99	69.06
83.33	83.33	85.05	82.54	84.13	83.33	84.13	84.13	82.54	84.13	84.13	83.33	83.33	84.13	84.13	83.33	81.75	83.47	84.3	84.13	84.13	83.33	85.12	100	99.21	100	86.51	78.79	80.95	67.2
85.36	85.08	85.84	85.36	85.91	85.64	86.19	86.19	85.64	85.91	85.64	85.91	86.19	86.46	86.19	84.89	84.07	83.57	93.85	93.39	94.21	93.11	93.85	85.67	85.4	86.51	100	79	83.43	74.37
77.57	77.26	78.23	77.88	78.5	78.82	78.82	78.82	77.57	77.88	77.88	78.5	78.19	78.5	79.13	77.02	76.4	76.9	78.48	78.19	78.5	77.88	78.48	76.64	76.32	78.79	79	100	80.86	70.72
83.79	83.79	83.83	83.79	84.34	84.62	84.34	84.34	84.07	84.34	84.07	84.34	84.34	84.62	85.16	83.38	82.56	80.61	80.83	80.55	81.1	80.6	81.11	77.26	76.99	80.95	83.43	80.86	100	76.57
75.35	74.79	79.88	74.79	75.35	75.62	75.62	75.62	75.07	75.62	75.07	75.62	75.35	75.62	75.62	73.2	72.65	71.91	70.87	70.72	71.55	70.8	71.15	69.34	69.06	67.2	74.37	70.72	76.57	100

Table S5: Sequence identities (%) of Ffsc6 and its homologues.

**BKA55DRAFT_678444**	**FOIG_14796**	**FOYG_13912**	**FMAN_13218**	**Ffsc6(FFUJ_10353)**	**FGLOB1_9181**	**FPRO_13861**	**FDENT_4193**	**FPCIR_13313**	**FMEXI_2109**	**FSUBG_7935**	**FACUT_6710**	**FMUND_9317**	**FNAPI_6680**	**FPANT_6568**	**FVEG_13707**	**J7337_012260**	**FSARC_8230**	**jgi.p_Fustri1_457669**	**BKA59DRAFT_488114**	**FPOAC1_005877**	**FVRRES_06486**	**HYE67_001787**	**FGSG_03494**
100	95.15	94.61	88.68	88.41	85.35	88.95	86.46	85.68	85.48	79.51	84.37	87.4	83.56	84.11	84.66	84.66	67.31	74.29	75.34	70.64	71.23	68.22	67.67
95.15	100	98.38	92.45	92.18	87.61	92.18	89.23	88.65	87.67	81.13	86.25	89.59	86.58	86.85	87.4	87.67	65.66	74.58	74.79	70.64	71.23	68.22	67.67
94.61	98.38	100	92.99	92.72	88.17	92.45	89.5	88.38	87.4	80.86	86.52	89.32	86.3	86.58	87.12	87.4	65.66	74.29	74.52	70.36	70.96	67.95	67.67
88.68	92.45	92.99	100	98.38	92.96	97.3	89.5	88.65	87.67	81.4	84.64	89.32	86.58	87.95	87.95	88.49	64.84	71.75	71.51	68.98	68.49	65.75	65.21
88.41	92.18	92.72	98.38	100	94.37	98.92	89.78	88.92	87.95	81.67	85.18	89.59	86.85	88.22	88.22	88.77	64.56	72.32	72.05	69.25	69.04	66.3	65.75
85.35	87.61	88.17	92.96	94.37	100	94.37	85.31	85.88	84.18	84.7	90.16	85.88	83.33	84.75	83.9	84.46	64.76	70.2	71.35	68.19	68.48	65.62	65.04
88.95	92.18	92.45	97.3	98.92	94.37	100	89.78	88.92	87.95	81.67	85.18	89.59	86.85	88.22	87.67	88.22	65.38	72.32	72.6	69.81	69.59	66.85	66.3
86.46	89.23	89.5	89.5	89.78	85.31	89.78	100	97.53	92.86	87.64	85.16	94.51	92.58	92.31	92.31	92.86	63.79	69.86	70.11	67.69	68.72	66.2	65.36
85.68	88.65	88.38	88.65	88.92	85.88	88.92	97.53	100	92.18	81.59	78.61	93.26	92.57	91.91	91.91	92.45	63.39	70.42	69.4	67.49	67.49	64.21	63.39
85.48	87.67	87.4	87.67	87.95	84.18	87.95	92.86	92.18	100	88.14	82.21	95.15	91.64	92.72	92.99	93.53	62.88	70.99	71.19	66.76	67.04	64.54	65.1
79.51	81.13	80.86	81.4	81.67	84.7	81.67	87.64	81.59	88.14	100	84.73	87.33	81.77	85.44	85.44	85.98	60.92	66.67	66.04	63.04	63.61	60.38	59.84
84.37	86.25	86.52	84.64	85.18	90.16	85.18	85.16	78.61	82.21	84.73	100	84.37	77.47	81.67	81.13	81.67	61.14	69.75	69.29	63.84	64.67	61.96	61.14
87.4	89.59	89.32	89.32	89.59	85.88	89.59	94.51	93.26	95.15	87.33	84.37	100	94.88	96.23	95.69	96.23	63.43	70.99	71.19	67.87	68.42	65.65	64.82
83.56	86.58	86.3	86.58	86.85	83.33	86.85	92.58	92.57	91.64	81.77	77.47	94.88	100	95.42	93.8	94.34	62.6	68.45	68.7	66.2	66.76	64.54	63.16
84.11	86.85	86.58	87.95	88.22	84.75	88.22	92.31	91.91	92.72	85.44	81.67	96.23	95.42	100	96.23	96.77	61.5	69.3	69.53	66.76	67.31	64.54	63.99
84.66	87.4	87.12	87.95	88.22	83.9	87.67	92.31	91.91	92.99	85.44	81.13	95.69	93.8	96.23	100	99.19	62.05	68.45	68.7	65.93	66.48	63.71	63.16
84.66	87.67	87.4	88.49	88.77	84.46	88.22	92.86	92.45	93.53	85.98	81.67	96.23	94.34	96.77	99.19	100	62.05	68.73	68.98	65.93	66.48	63.71	63.16
67.31	65.66	65.66	64.84	64.56	64.76	65.38	63.79	63.39	62.88	60.92	61.14	63.43	62.6	61.5	62.05	62.05	100	70	70.27	68.94	70	68.11	67.57
74.29	74.58	74.29	71.75	72.32	70.2	72.32	69.86	70.42	70.99	66.67	69.75	70.99	68.45	69.3	68.45	68.73	70	100	93.89	80.56	80.28	79.72	79.72
75.34	74.79	74.52	71.51	72.05	71.35	72.6	70.11	69.4	71.19	66.04	69.29	71.19	68.7	69.53	68.7	68.98	70.27	93.89	100	80.11	80.86	80.32	80.32
70.64	70.64	70.36	68.98	69.25	68.19	69.81	67.69	67.49	66.76	63.04	63.84	67.87	66.2	66.76	65.93	65.93	68.94	80.56	80.11	100	94.55	91.83	91.28
71.23	71.23	70.96	68.49	69.04	68.48	69.59	68.72	67.49	67.04	63.61	64.67	68.42	66.76	67.31	66.48	66.48	70	80.28	80.86	94.55	100	92.45	91.91
68.22	68.22	67.95	65.75	66.3	65.62	66.85	66.2	64.21	64.54	60.38	61.96	65.65	64.54	64.54	63.71	63.71	68.11	79.72	80.32	91.83	92.45	100	98.65
67.67	67.67	67.67	65.21	65.75	65.04	66.3	65.36	63.39	65.1	59.84	61.14	64.82	63.16	63.99	63.16	63.16	67.57	79.72	80.32	91.28	91.91	98.65	100

Table S6: Sequence identities (%) of STC5 and homologues.

**FPCIR_3536**	**FPANT_9745**	**FDENT_6295**	**FACUT_9293**	**FSUBG_1573**	**FANTH_5434**	**FMEXI_4348**	**FPRO_15464**	**STC5(FFUJ_11739)**	**FGLOB1_2359**	**FMAN_14887**	**FOYG_15697**	**FOIG_10183**	**FGSG_08181**	**HYE67_00260**	**FPSE_10885**	**FVRES_04682**	**FPOAC1_004210**	**FSARC_5213**
100	97.76	96.76	97.51	96.51	96.51	95.76	96.51	95.26	96.01	97.26	94.51	94.76	88.53	89.53	88.78	86.28	87.03	80.1
97.76	100	96.76	98.25	97.51	97.51	96.76	97.26	96.01	97.01	97.51	94.76	95.26	88.53	89.53	89.28	86.53	87.53	80.37
96.76	96.76	100	97.26	96.26	96.26	95.76	96.51	95.26	96.01	97.01	95.01	95.51	88	89.03	88.28	85.79	86.78	79.58
97.51	98.25	97.26	100	97.76	97.76	97.26	98	96.76	97.51	98.75	95.76	95.76	89.6	90.52	89.53	87.03	88.03	80.1
96.51	97.51	96.26	97.76	100	100	98	97.26	96.01	97.26	97.51	94.76	95.01	88.53	89.53	89.03	86.78	87.78	80.89
96.51	97.51	96.26	97.76	100	100	98	97.26	96.01	97.26	97.51	94.76	95.01	88.53	89.53	89.03	86.78	87.78	80.89
95.76	96.76	95.76	97.26	98	98	100	96.76	96.01	96.76	97.51	94.26	94.51	87.2	88.28	87.78	85.29	86.78	79.58
96.51	97.26	96.51	98	97.26	97.26	96.76	100	98.25	99	98.75	95.76	96.51	88.27	89.03	88.78	87.78	88.28	80.89
95.26	96.01	95.26	96.76	96.01	96.01	96.01	98.25	100	97.76	97.51	94.51	95.26	87.2	88.28	87.78	86.53	87.03	79.84
96.01	97.01	96.01	97.51	97.26	97.26	96.76	99	97.76	100	98.25	95.26	96.51	88	88.53	88.03	86.78	87.28	80.63
97.26	97.51	97.01	98.75	97.51	97.51	97.51	98.75	97.51	98.25	100	95.76	96.01	89.07	89.78	88.78	86.78	87.78	80.37
94.51	94.76	95.01	95.76	94.76	94.76	94.26	95.76	94.51	95.26	95.76	100	98.25	87.47	87.53	87.03	85.54	85.79	80.89
94.76	95.26	95.51	95.76	95.01	95.01	94.51	96.51	95.26	96.51	96.01	98.25	100	86.67	87.53	87.03	86.28	86.53	81.68
88.53	88.53	88	89.6	88.53	88.53	87.2	88.27	87.2	88	89.07	87.47	86.67	100	98.94	96.54	90.67	91.2	78.37
89.53	89.53	89.03	90.52	89.53	89.53	88.28	89.03	88.28	88.53	89.78	87.53	87.53	98.94	100	96.77	91.52	92.02	79.32
88.78	89.28	88.28	89.53	89.03	89.03	87.78	88.78	87.78	88.03	88.78	87.03	87.03	96.54	96.77	100	92.02	92.52	79.58
86.28	86.53	85.79	87.03	86.78	86.78	85.29	87.78	86.53	86.78	86.78	85.54	86.28	90.67	91.52	92.02	100	98.25	78.53
87.03	87.53	86.78	88.03	87.78	87.78	86.78	88.28	87.03	87.28	87.78	85.79	86.53	91.2	92.02	92.52	98.25	100	78.8
80.1	80.37	79.58	80.1	80.89	80.89	79.58	80.89	79.84	80.63	80.37	80.89	81.68	78.37	79.32	79.58	78.53	78.8	100

Table S7: Sequence identities (%) of STC3 and homologues.

**FVRRES_13838**	**FGLOB1_629**	**STC3(FFUJ_04067)**	**FPRO_03449**
100	82.38	81.9	81.43
82.38	100	99.16	98.88
81.9	99.16	100	98.6
81.43	98.88	98.6	100

Table S8: Sequence identities (%) of CLM1 and homologues.

**FPSE_07410**	**HYE67_011182**	**CLM1(FGSG_10397)**	**FVRRES_13751**	**FDECE_15583**	**jgi.p_Fustri1_646467**	**BKA59DRAFT_555427**
100	99.12	98.23	76.7	71.04	71.6	71.98
99.12	100	99.12	76.7	71.04	71.89	72.27
98.23	99.12	100	76.7	71.34	71.6	71.98
76.7	76.7	76.7	100	72.97	75.51	76.45
71.04	71.04	71.34	72.97	100	78.57	79.53
71.6	71.89	71.6	75.51	78.57	100	95.39
71.98	72.27	71.98	76.45	79.53	95.39	100

Table S9: Sequence identities (%) of TRI5 and homologues.

**FPSE_12160**	**HYE67_001832**	**FGSG_03537**	**FPOAC1_005923**	**FVRRES_06532**	**TRI5(TOX5)**	**B0J16DRAFT_404726**
100	97.07	98.13	91.73	92.27	92	85.52
97.07	100	97.6	90.4	90.93	90.4	84.99
98.13	97.6	100	91.47	92	91.47	86.33
91.73	90.4	91.47	100	97.88	96.82	88.24
92.27	90.93	92	97.88	100	98.68	87.97
92	90.4	91.47	96.82	98.68	100	87.17
85.52	84.99	86.33	88.24	87.97	87.17	100

Table S10: Sequence identities (%) of FlvE and homologues.

**LCI18_010754**	**LCI18_011228**	**NECHADRAFT_97884**	**NCS54_01458300**	**NCS57_01441900**	**flvE**
100.00	100.00	95.15	91.67	96.52	50.90
100.00	100.00	95.15	91.67	96.52	50.90
95.15	95.15	100.00	90.29	95.66	52.25
91.67	91.67	90.29	100.00	93.06	47.70
96.52	96.52	95.66	93.06	100.00	50.65
50.90	50.90	52.25	47.70	50.65	100.00

Table S11: Sequence identities (%) of DpfgD and homologues.

**FPSE_10841**	**dpfgD(FGSG_04591)**	**jgi.p_Fustri1_542810**	**BKA55DRAFT_506145**
100	93.23	87.38	89.73
93.23	100	86.24	87.92
87.38	86.24	100	93.88
89.73	87.92	93.88	100

Table S12: Sequence identities (%) of GGS and homologues.

FDECE_12973	NCS57_0750400	NCS54_0710300	LCI18_011732	B0J15DRAFT_391762	NECHADRAFT_69530	FSARC_14424	FGRMN_4462	jgi.p_Fustri1_13322	BKA59DRAFT_462499	B0J16DRAFT_67742	FVRRES_03520	FPOAC1_003180	HYE67_010879	FPSE_00459	FGSG_10097	BKA55DRAFT_508582	FOIG_07211	FOYG_08127	FACUT_3379	FMEXI_10918	FANTH_4007	FSUBG_9424	FPCIR_9600	FGLOB1_11967	FPRO_07922	FFUJ_07352	GGS	FMAN_07528	FDENT_13338	FMUND_9065	J7337_005732	FVEG_02819	FPANT_8144	FNAPI_13184
100	88.7	88.7	88.46	88.46	87.98	86.47	83.09	83.33	83.57	82.61	82.61	82.61	81.64	81.4	82.13	84.62	84.13	84.13	83.41	83.41	85.28	83.41	83.17	83.41	83.41	83.41	82.93	83.17	83.41	83.41	83.17	83.41	83.65	82.93
88.7	100	99.52	99.04	98.81	98.33	90.1	86.71	87.2	87.68	85.51	86.47	86.23	85.27	85.02	85.75	87.26	87.5	87.5	86.54	86.78	87.82	86.78	86.54	87.02	87.02	87.02	86.54	87.02	86.78	86.78	86.3	86.78	87.02	86.3
88.7	99.52	100	99.04	99.29	98.33	89.86	86.47	86.96	87.44	85.27	86.47	86.23	85.51	85.27	85.99	87.26	87.5	87.5	86.54	86.78	87.82	86.78	86.54	87.02	87.02	87.02	86.54	87.02	86.78	86.78	86.3	86.78	87.02	86.3
88.46	99.04	99.04	100	98.8	98.33	90.1	86.23	86.71	87.2	85.02	86.47	86.23	85.51	85.27	85.99	87.02	87.26	87.26	86.3	86.54	87.56	86.54	86.3	86.78	86.78	86.78	86.3	86.78	86.54	86.54	86.06	86.54	86.78	86.06
88.46	98.81	99.29	98.8	100	98.57	89.61	85.75	86.23	86.71	84.54	85.75	85.51	84.78	84.54	85.27	86.54	86.78	86.78	85.82	86.06	87.31	86.06	85.82	86.3	86.3	86.3	85.82	86.3	86.06	86.06	85.58	86.06	86.3	85.58
87.98	98.33	98.33	98.33	98.57	100	88.89	85.02	85.51	85.99	84.3	85.02	84.78	84.06	83.82	84.54	86.3	86.54	86.54	85.58	85.82	86.8	85.82	85.58	86.06	86.06	86.06	85.58	86.06	85.82	85.82	85.34	85.82	86.06	85.34
86.47	90.1	89.86	90.1	89.61	88.89	100	89.9	89.9	90.38	89.18	88.7	88.94	88.22	87.98	88.22	91.11	91.59	91.59	91.11	90.87	91.62	91.11	90.87	91.11	91.11	91.11	90.62	90.87	91.11	91.11	90.87	91.11	91.11	90.62
83.09	86.71	86.47	86.23	85.75	85.02	89.9	100	97.12	97.6	89.66	90.38	90.62	89.42	89.42	89.18	91.59	92.07	92.07	91.59	91.83	92.39	91.59	91.35	90.87	90.87	90.87	90.38	90.62	91.35	91.11	91.11	91.35	91.59	90.87
83.33	87.2	86.96	86.71	86.23	85.51	89.9	97.12	100	99.52	89.9	90.62	90.87	89.66	89.66	89.42	92.07	92.07	92.07	91.59	92.07	92.39	91.83	91.35	91.35	91.35	91.35	90.87	91.11	91.35	91.11	91.11	91.35	91.59	90.87
83.57	87.68	87.44	87.2	86.71	85.99	90.38	97.6	99.52	100	90.14	90.87	91.11	89.9	89.9	89.66	92.07	92.55	92.55	92.07	92.55	92.89	92.31	91.83	91.83	91.83	91.83	91.35	91.59	91.83	91.59	91.59	91.83	92.07	91.35
82.61	85.51	85.27	85.02	84.54	84.3	89.18	89.66	89.9	90.14	100	94.23	93.75	93.27	93.03	93.03	92.07	92.07	92.07	90.87	91.11	91.37	91.35	90.87	90.87	90.87	90.87	90.38	90.62	90.87	90.62	90.38	90.87	90.87	90.38
82.61	86.47	86.47	86.47	85.75	85.02	88.7	90.38	90.62	90.87	94.23	100	98.32	97.12	96.88	96.88	92.79	93.03	93.03	91.59	92.07	92.13	92.07	91.59	92.31	92.07	92.07	91.59	91.83	91.59	91.35	91.35	91.59	91.59	91.11
82.61	86.23	86.23	86.23	85.51	84.78	88.94	90.62	90.87	91.11	93.75	98.32	100	97.6	97.36	97.36	93.03	93.27	93.27	91.83	92.31	92.39	92.31	91.83	92.07	91.83	91.83	91.35	91.59	91.83	91.59	91.59	91.83	91.83	91.35
81.64	85.27	85.51	85.51	84.78	84.06	88.22	89.42	89.66	89.9	93.27	97.12	97.6	100	99.28	99.28	92.07	92.07	92.07	90.87	91.35	91.37	91.35	90.87	90.87	90.87	90.87	90.38	90.62	90.87	90.62	90.38	90.87	90.87	90.38
81.4	85.02	85.27	85.27	84.54	83.82	87.98	89.42	89.66	89.9	93.03	96.88	97.36	99.28	100	99.04	91.83	91.83	91.83	90.62	91.11	91.12	91.11	90.62	90.62	90.62	90.62	90.14	90.38	90.62	90.38	90.14	90.62	90.62	90.14
82.13	85.75	85.99	85.99	85.27	84.54	88.22	89.18	89.42	89.66	93.03	96.88	97.36	99.28	99.04	100	91.83	91.83	91.83	90.62	91.11	91.62	91.11	90.62	90.62	90.62	90.62	90.14	90.38	90.62	90.62	90.38	90.87	84.09	90.38
84.62	87.26	87.26	87.02	86.54	86.3	91.11	91.59	92.07	92.07	92.07	92.79	93.03	92.07	91.83	91.83	100	98.8	98.8	97.61	97.37	97.73	97.61	97.61	97.13	97.13	97.13	96.65	96.89	97.61	97.37	97.13	97.61	97.13	97.13
84.13	87.5	87.5	87.26	86.78	86.54	91.59	92.07	92.07	92.55	92.07	93.03	93.27	92.07	91.83	91.83	98.8	100	100	98.56	98.33	98.74	98.56	98.56	98.33	98.09	98.09	97.61	97.85	98.56	98.33	98.33	98.56	98.09	98.09
84.13	87.5	87.5	87.26	86.78	86.54	91.59	92.07	92.07	92.55	92.07	93.03	93.27	92.07	91.83	91.83	98.8	100	100	98.56	98.33	98.74	98.56	98.56	98.33	98.09	98.09	97.61	97.85	98.56	98.33	98.33	98.56	98.09	98.09

83.41	86.54	86.54	86.3	85.82	85.58	91.11	91.59	91.59	92.07	90.87	91.59	91.83	90.87	90.62	90.62	97.61	98.56	98.56	100	99.28	99.75	99.52	99.52	98.8	99.04	99.04	98.56	98.8	99.52	99.28	99.04	99.52	99.04	99.04
83.41	86.78	86.78	86.54	86.06	85.82	90.87	91.83	92.07	92.55	91.11	92.07	92.31	91.35	91.11	91.11	97.37	98.33	98.33	99.28	100	99.75	99.76	99.28	98.56	98.8	98.8	98.33	98.56	99.28	99.04	98.56	99.28	98.8	98.8
85.28	87.82	87.82	87.56	87.31	86.8	91.62	92.39	92.39	92.89	91.37	92.13	92.39	91.37	91.12	91.62	97.73	98.74	98.74	99.75	99.75	100	100	99.49	98.74	98.99	98.99	98.48	98.74	99.49	99.49	99.24	99.75	99.24	99.24
83.41	86.78	86.78	86.54	86.06	85.82	91.11	91.59	91.83	92.31	91.35	92.07	92.31	91.35	91.11	91.11	97.61	98.56	98.56	99.52	99.76	100	100	99.52	98.8	99.04	99.04	98.56	98.8	99.52	99.28	98.8	99.52	99.04	99.04
83.17	86.54	86.54	86.3	85.82	85.58	90.87	91.35	91.35	91.83	90.87	91.59	91.83	90.87	90.62	90.62	97.61	98.56	98.56	99.52	99.28	99.49	99.52	100	98.8	99.04	99.04	98.56	98.8	99.52	99.28	98.8	99.52	99.04	99.04
83.41	87.02	87.02	86.78	86.3	86.06	91.11	90.87	91.35	91.83	90.87	92.31	92.07	90.87	90.62	90.62	97.13	98.33	98.33	98.8	98.56	98.74	98.8	98.8	100	99.76	99.76	99.28	99.52	98.8	98.56	98.56	98.8	98.33	98.33
83.41	87.02	87.02	86.78	86.3	86.06	91.11	90.87	91.35	91.83	90.87	92.07	91.83	90.87	90.62	90.62	97.13	98.09	98.09	99.04	98.8	98.99	99.04	99.04	99.76	100	100	99.52	99.76	99.04	98.8	98.33	99.04	98.56	98.56
83.41	87.02	87.02	86.78	86.3	86.06	91.11	90.87	91.35	91.83	90.87	92.07	91.83	90.87	90.62	90.62	97.13	98.09	98.09	99.04	98.8	98.99	99.04	99.04	99.76	100	100	99.52	99.76	99.04	98.8	98.33	99.04	98.56	98.56
82.93	86.54	86.54	86.3	85.82	85.58	90.62	90.38	90.87	91.35	90.38	91.59	91.35	90.38	90.14	90.14	96.65	97.61	97.61	98.56	98.33	98.48	98.56	98.56	99.28	99.52	99.52	100	99.28	98.56	98.33	97.85	98.56	98.09	98.09
83.17	87.02	87.02	86.78	86.3	86.06	90.87	90.62	91.11	91.59	90.62	91.83	91.59	90.62	90.38	90.38	96.89	97.85	97.85	98.8	98.56	98.74	98.8	98.8	99.52	99.76	99.76	99.28	100	98.8	98.56	98.09	98.8	98.33	98.33
83.41	86.78	86.78	86.54	86.06	85.82	91.11	91.35	91.35	91.83	90.87	91.59	91.83	90.87	90.62	90.62	97.61	98.56	98.56	99.52	99.28	99.49	99.52	99.52	98.8	99.04	99.04	98.56	98.8	100	99.76	98.8	99.52	99.04	99.04
83.41	86.78	86.78	86.54	86.06	85.82	91.11	91.11	91.11	91.59	90.62	91.35	91.59	90.62	90.38	90.62	97.37	98.33	98.33	99.28	99.04	99.49	99.28	99.28	98.56	98.8	98.8	98.33	98.56	99.76	100	99.04	99.76	99.28	99.28
83.17	86.3	86.3	86.06	85.58	85.34	90.87	91.11	91.11	91.59	90.38	91.35	91.59	90.38	90.14	90.38	97.13	98.33	98.33	99.04	98.56	99.24	98.8	98.8	98.56	98.33	98.33	97.85	98.09	98.8	99.04	100	99.28	98.8	99.04
83.41	86.78	86.78	86.54	86.06	85.82	91.11	91.35	91.35	91.83	90.87	91.59	91.83	90.87	90.62	90.87	97.61	98.56	98.56	99.52	99.28	99.75	99.52	99.52	98.8	99.04	99.04	98.56	98.8	99.52	99.76	99.28	100	99.52	99.52
83.65	87.02	87.02	86.78	86.3	86.06	91.11	91.59	91.59	92.07	90.87	91.59	91.83	90.87	90.62	84.09	97.13	98.09	98.09	99.04	98.8	99.24	99.04	99.04	98.33	98.56	98.56	98.09	98.33	99.04	99.28	98.8	99.52	100	99.04
82.93	86.3	86.3	86.06	85.58	85.34	90.62	90.87	90.87	91.35	90.38	91.11	91.35	90.38	90.14	90.38	97.13	98.09	98.09	99.04	98.8	99.24	99.04	99.04	98.33	98.56	98.56	98.09	98.33	99.04	99.28	99.04	99.52	99.04	100

Table S13: Sequence identities (%) of CPS/KS and homologues.

**FSARC_13126**	**jgi.p_Fustri1_538881**	**FDECE_7075**	**LCI18_013895**	**FNAPI_4141**	**FOIG_09083**	**FGLOB1_6186**	**FPRO_08705**	**CPS/KS(FFUJ_14336)**	**FMAN_08250**	**FOYG_08942**	**FACUT_8682**	**FPRO_16062**	**FANTH_6278**	**FSUBG_10000**	**FMEXI_710**	**FDENT_14086**	**FMUND_15697**	**J7337_005030**	**FPANT_11050**
100	54.24	57.43	49.33	41.67	43.54	53.77	53.7	52.91	52.28	52.7	52.54	52.54	54.07	53.62	53.33	52.28	52.7	52.12	53.02
54.24	100	70.04	65.15	51.98	55.8	64.76	65.01	65.78	65.89	65.89	65.64	66.17	66.63	65.31	64.83	65.36	66.31	65.85	65.15
57.43	70.04	100	77.15	54.56	59.08	66.38	66.6	66.74	66.53	66.53	66.6	65.33	66.63	65.22	65.26	65.58	66.63	66.63	66.53
49.33	65.15	77.15	100	64.4	57.62	60.6	61.97	61.63	61.43	63.57	62.14	61.75	63.18	60.85	61.05	61.43	62.21	59.88	60.85
41.67	51.98	54.56	64.4	100	75.72	62.55	63.29	62.9	63.1	63.29	65.28	64.09	62.9	60.97	61.51	66.27	65.87	67.66	68.25
43.54	55.8	59.08	57.62	75.72	100	76.19	77.34	79.08	78.43	80.83	78.38	78	78.43	75.82	76.91	78.87	79.74	77.56	78.65
53.77	64.76	66.38	60.6	62.55	76.19	100	96.9	85.55	85.01	85.12	84.57	83.71	84.26	82.85	83.94	83.4	85.97	83.3	84.15
53.7	65.01	66.6	61.97	63.29	77.34	96.9	100	85.61	84.87	84.98	84.54	83.49	84.14	82.54	83.61	83.19	85.92	82.85	83.93
52.91	65.78	66.74	61.63	62.9	79.08	85.55	85.61	100	89.08	86.13	85.91	85.28	84.66	83.07	84.14	84.35	86.97	83.74	85.08
52.28	65.89	66.53	61.43	63.1	78.43	85.01	84.87	89.08	100	85.71	86.44	86.12	85.92	84.44	85.61	84.77	86.34	83.52	85.5
52.7	65.89	66.53	63.57	63.29	80.83	85.12	84.98	86.13	85.71	100	85.17	85.28	85.4	83.81	84.45	83.82	86.24	82.63	84.24
52.54	65.64	66.6	62.14	65.28	78.38	84.57	84.54	85.91	86.44	85.17	100	85.47	85.49	84.22	84.75	85.28	87.07	86.62	86.86
52.54	66.17	65.33	61.75	64.09	78	83.71	83.49	85.28	86.12	85.28	85.47	100	89.38	89.19	89.48	88.01	86.65	84.06	84.23
54.07	66.63	66.63	63.18	62.9	78.43	84.26	84.14	84.66	85.92	85.4	85.49	89.38	100	91.96	91.7	87.39	86.45	83.96	84.45
53.62	65.31	65.22	60.85	60.97	75.82	82.85	82.54	83.07	84.44	83.81	84.22	89.19	91.96	100	92.17	86.46	84.76	81.93	83.07
53.33	64.83	65.26	61.05	61.51	76.91	83.94	83.61	84.14	85.61	84.45	84.75	89.48	91.7	92.17	100	87.18	85.71	83.52	84.35
52.28	65.36	65.58	61.43	66.27	78.87	83.4	83.19	84.35	84.77	83.82	85.28	88.01	87.39	86.46	87.18	100	88.55	84.97	86.03
52.7	66.31	66.63	62.21	65.87	79.74	85.97	85.92	86.97	86.34	86.24	87.07	86.65	86.45	84.76	85.71	88.55	100	86.97	89.39
52.12	65.85	66.63	59.88	67.66	77.56	83.3	82.85	83.74	83.52	82.63	86.62	84.06	83.96	81.93	83.52	84.97	86.97	100	90.98
53.02	65.15	66.53	60.85	68.25	78.65	84.15	83.93	85.08	85.5	84.24	86.86	84.23	84.45	83.07	84.35	86.03	89.39	90.98	100

Table S14: Sequence identities (%) of DpfgB and homologues.

DER45DRAFT_618020	KAF25_003423	HG530_013942	FAVG1_12698	H9Q72_003567	H9Q73_003817	H9Q70_014089	TPAR_03748	XA68_13016	FNYG_13150	F53441_8896	FOMA001_g18868	LZL87_013916	DER46DRAFT_690367	Forpe1208_v011544	FCULG_00007167	FPSE5266_10837	MDCFG8_LOCUS587785	FPSE_10837	MDCFG202_LOCUS518736	FAUST_11421	MDCFU1_LOCUS567934	dpfgB_FGSG_12222_	FGRAMCML3066_01t15717.1	MDCFG5_LOCUS178956	FGRAMPH1_01T15659	NXS19_008005
100	98.34	98.76	98.35	91.32	91.86	90.78	80.58	76.57	90.4	87.19	93.14	90.99	92.61	93.04	91.21	91.63	91.21	88.78	91.21	91.21	91.21	92.35	90.79	90.38	91.56	65.35
98.34	100	99.17	98.76	92.12	93.02	91.71	81.74	76.99	90.96	88.38	94.29	92.24	93.48	93.91	90.04	90.46	90.46	87.98	90.46	90.46	90.46	91.33	90.04	89.63	90.79	64.22
98.76	99.17	100	99.59	92.15	93.02	91.75	80.99	76.57	90.96	88.02	94.29	91.85	93.48	93.91	91.21	91.63	91.21	88.78	91.21	91.21	91.21	92.35	90.79	90.38	91.56	65.35
98.35	98.76	99.59	100	92.15	93.02	91.75	80.99	76.57	90.96	88.02	93.71	91.42	93.04	93.48	91.21	91.63	91.21	88.78	91.21	91.21	91.21	92.35	90.79	90.38	91.56	65.35
91.32	92.12	92.15	92.15	100	99.42	99.51	80.58	75.73	89.27	84.3	91.43	88.41	90	89.57	89.54	89.96	89.96	86.83	89.96	89.96	89.96	91.18	89.54	89.12	89.33	63.6
91.86	93.02	93.02	93.02	99.42	100	100	81.4	78.49	90.7	90.7	91.86	91.86	91.86	91.86	90	90.59	90.59	89.12	90.59	90.59	90.59	90.59	90.59	90	90.59	80.81
90.78	91.71	91.75	91.75	99.51	100	100	79.61	72.41	86.52	81.55	90.65	87.31	89.18	88.66	87.86	88.35	88.35	84.3	88.35	88.35	88.35	89.05	87.86	87.38	87.5	65.62
80.58	81.74	80.99	80.99	80.58	81.4	79.61	100	77.82	77.97	77.37	80.57	78.63	80.43	80.43	82.01	82.43	82.85	80.98	82.85	82.85	82.85	81.76	82.43	82.43	82.67	58.77
76.57	76.99	76.57	76.57	75.73	78.49	72.41	77.82	100	75.71	74.48	76.57	75.22	75.65	76.09	77.54	77.54	77.54	76.59	77.54	77.54	77.54	78.82	77.54	77.12	78.22	57.02
90.4	90.96	90.96	90.96	89.27	90.7	86.52	77.97	75.71	100	92.09	93.71	91.53	91.53	91.53	88.51	90.23	90.23	88.08	90.23	90.23	90.23	92.35	90.23	90.23	90.23	81.36
87.19	88.38	88.02	88.02	84.3	90.7	81.55	77.37	74.48	92.09	100	93.71	91.14	88.02	88.43	85.77	87.03	87.03	89.76	87.03	87.03	87.03	92.94	86.61	86.19	92.44	66.23
93.14	94.29	94.29	93.71	91.43	91.86	90.65	80.57	76.57	93.71	93.71	100	98.86	98.86	98.86	93.02	94.77	94.77	93.29	94.77	94.77	94.77	95.88	94.77	94.19	94.77	85.14
90.99	92.24	91.85	91.42	88.41	91.86	87.31	78.63	75.22	91.53	91.14	98.86	100	93.39	92.98	90	91.3	91.3	93.97	91.3	91.3	91.3	95.88	90.87	90.43	94.14	69.82
92.61	93.48	93.48	93.04	90	91.86	89.18	80.43	75.65	91.53	88.02	98.86	93.39	100	99.17	92.07	93.39	93.39	93.47	93.39	93.39	93.39	95.88	92.95	92.51	94.59	69.82
93.04	93.91	93.91	93.48	89.57	91.86	88.66	80.43	76.09	91.53	88.43	98.86	92.98	99.17	100	92.51	93.83	93.83	93.97	93.83	93.83	93.83	95.88	93.39	92.95	95.05	69.82
91.21	90.04	91.21	91.21	89.54	90	87.86	82.01	77.54	88.51	85.77	93.02	90	92.07	92.51	100	98.76	98.35	94.71	98.35	98.35	98.35	98.27	97.93	97.52	96.93	70.18
91.63	90.46	91.63	91.63	89.96	90.59	88.35	82.43	77.54	90.23	87.03	94.77	91.3	93.39	93.83	98.76	100	99.59	96.15	99.59	99.59	99.59	100	99.17	98.76	98.25	71.49
91.21	90.46	91.21	91.21	89.96	90.59	88.35	82.85	77.54	90.23	87.03	94.77	91.3	93.39	93.83	98.35	99.59	100	96.15	99.81	100	99.63	100	99.44	99.26	98.86	71.49
88.78	87.98	88.78	88.78	86.83	89.12	84.3	80.98	76.59	88.08	89.76	93.29	93.97	93.47	93.97	94.71	96.15	96.15	100	96.15	96.15	96.15	99.33	95.67	95.19	97.07	68.29
91.21	90.46	91.21	91.21	89.96	90.59	88.35	82.85	77.54	90.23	87.03	94.77	91.3	93.39	93.83	98.35	99.59	99.81	96.15	100	100	99.9	100	99.63	97.86	99.05	71.49
91.21	90.46	91.21	91.21	89.96	90.59	88.35	82.85	77.54	90.23	87.03	94.77	91.3	93.39	93.83	98.35	99.59	100	96.15	100	100	100	100	99.59	99.17	98.25	71.49
91.21	90.46	91.21	91.21	89.96	90.59	88.35	82.85	77.54	90.23	87.03	94.77	91.3	93.39	93.83	98.35	99.59	99.63	96.15	99.9	100	100	100	99.81	97.97	99.24	71.49
92.35	91.33	92.35	92.35	91.18	90.59	89.05	81.76	78.82	92.35	92.94	95.88	95.88	95.88	95.88	98.27	100	100	99.33	100	100	100	100	100	99.42	100	90.75
90.79	90.04	90.79	90.79	89.54	90.59	87.86	82.43	77.54	90.23	86.61	94.77	90.87	92.95	93.39	97.93	99.17	99.44	95.67	99.63	99.59	99.81	100	100	99.44	99.05	71.49
90.38	89.63	90.38	90.38	89.12	90	87.38	82.43	77.12	90.23	86.19	94.19	90.43	92.51	92.95	97.52	98.76	99.26	95.19	97.86	99.17	97.97	99.42	99.44	100	99.24	71.05
91.56	90.79	91.56	91.56	89.33	90.59	87.5	82.67	78.22	90.23	92.44	94.77	94.14	94.59	95.05	96.93	98.25	98.86	97.07	99.05	98.25	99.24	100	99.05	99.24	100	72.44
65.35	64.22	65.35	65.35	63.6	80.81	65.62	58.77	57.02	81.36	66.23	85.14	69.82	69.82	69.82	70.18	71.49	71.49	68.29	71.49	71.49	71.49	90.75	71.49	71.05	72.44	100

Table S15: Sequence identities (%) of FgMS, FoFs and homologues.

**FgMS**	**FGSG_01738**	**FoFs**	**FGRMN_7913**	**FPCIR_12113**	**FPANT_13888**
100	98.65	32.23	29.12	29.35	29.59
98.65	100	31.25	27.39	27.87	28.25
32.23	31.25	100	28.78	28.8	29.67
29.12	27.39	28.78	100	79.33	80.05
29.35	27.87	28.8	79.33	100	96.94
29.59	28.25	29.67	80.05	96.94	100

Table S16: Sequence identities (%) of ERG7 and homologues.

**ERG7(A0A0E0SP71)**	**LCI18_004508**	**NECHADRAFT_72055**	**B0J15DRAFT_490730**	**NCS57_01162100**	**B0J15DRAFT_467131**	**NCS54_01418400**	**NCS57_01400300**	**FDECE_3328**	**BKA59DRAFT_535453**	**FSARC_2617**
100	80.45	80.1	80.45	80.08	56.64	56.58	56.72	58.39	49.68	58.59
80.45	100	98.04	98.82	97.91	58.13	58.89	59.02	60.85	52.44	59.95
80.1	98.04	100	98.3	97.65	57.72	58.48	58.62	60.44	51.94	59.81
80.45	98.82	98.3	100	98.43	57.86	58.62	58.75	60.85	52.6	60.22
80.08	97.91	97.65	98.43	100	57.99	58.75	58.89	60.71	52.44	60.08
56.64	58.13	57.72	57.86	57.99	100	96.34	96.75	74.52	59.15	71.45
56.58	58.89	58.48	58.62	58.75	96.34	100	98.37	75.72	59.08	71.27
56.72	59.02	58.62	58.75	58.89	96.75	98.37	100	75.85	59.57	71.68
58.39	60.85	60.44	60.85	60.71	74.52	75.72	75.85	100	63.59	77.66
49.68	52.44	51.94	52.6	52.44	59.15	59.08	59.57	63.59	100	68.35
58.59	59.95	59.81	60.22	60.08	71.45	71.27	71.68	77.66	68.35	100

Table S17: Sequence identities (%) of CarRA and homologues.

**BKA55DRAFT_652263**	**FOYG_15802**	**FOIG_10085**	**FNAPI_9157**	**J7337_013307**	**FVEG_10718**	**FMUND_1581**	**FPANT_4577**	**FPCIR_12421**	**FDENT_7020**	**FACUT_1095**	**FPRO_15512**	**FGLOB1_6742**	**carRA (FFUJ_11802)**	**FMAN_14945**	**FMEXI_3381**	**FSUBG_7850**	**FANTH_6971**
100	96.72	97.59	95.69	95.69	95.69	94.66	96.38	96.03	93.45	95.69	95.17	94.48	95.52	94.83	95.17	95.17	94.07
96.72	100	98.79	96.38	96.03	96.03	95.34	96.72	96.03	93.79	96.72	96.55	95.86	96.9	96.21	96.55	96.21	95.56
97.59	98.79	100	97.24	96.9	96.9	95.86	97.59	96.9	94.83	97.24	97.07	96.38	97.41	96.72	97.07	96.72	95.74
95.69	96.38	97.24	100	97.93	97.93	97.24	98.62	97.93	95.69	97.93	97.41	97.07	98.1	97.76	98.45	97.76	97.04
95.69	96.03	96.9	97.93	100	99.14	97.24	98.62	97.93	95.17	98.1	97.07	96.72	97.76	97.07	97.41	97.41	96.48
95.69	96.03	96.9	97.93	99.14	100	96.72	98.45	97.76	95	97.93	96.9	96.55	97.59	97.07	97.41	97.24	96.48
94.66	95.34	95.86	97.24	97.24	96.72	100	98.28	97.24	94.83	97.41	96.38	96.38	97.07	96.72	97.07	97.41	96.48
96.38	96.72	97.59	98.62	98.62	98.45	98.28	100	98.97	96.21	98.79	97.76	97.41	98.45	97.76	98.1	98.45	97.59
96.03	96.03	96.9	97.93	97.93	97.76	97.24	98.97	100	96.03	98.28	97.59	97.24	98.28	97.59	97.93	98.28	97.41
93.45	93.79	94.83	95.69	95.17	95	94.83	96.21	96.03	100	95.52	95.17	94.48	95.52	95.17	95.52	95.69	94.63
95.69	96.72	97.24	97.93	98.1	97.93	97.41	98.79	98.28	95.52	100	98.1	97.76	98.79	98.1	98.45	98.45	97.59
95.17	96.55	97.07	97.41	97.07	96.9	96.38	97.76	97.59	95.17	98.1	100	98.79	98.45	97.76	98.1	97.76	96.85
94.48	95.86	96.38	97.07	96.72	96.55	96.38	97.41	97.24	94.48	97.76	98.79	100	98.1	97.41	98.1	97.76	96.85
95.52	96.9	97.41	98.1	97.76	97.59	97.07	98.45	98.28	95.52	98.79	98.45	98.1	100	98.45	98.79	98.45	97.59
94.83	96.21	96.72	97.76	97.07	97.07	96.72	97.76	97.59	95.17	98.1	97.76	97.41	98.45	100	98.97	98.62	97.59
95.17	96.55	97.07	98.45	97.41	97.41	97.07	98.1	97.93	95.52	98.45	98.1	98.1	98.79	98.97	100	99.31	98.15
95.17	96.21	96.72	97.76	97.41	97.24	97.41	98.45	98.28	95.69	98.45	97.76	97.76	98.45	98.62	99.31	100	98.7
94.07	95.56	95.74	97.04	96.48	96.48	96.48	97.59	97.41	94.63	97.59	96.85	96.85	97.59	97.59	98.15	98.7	100

Table S18: Sequence identities (%) of DMATS1 and homologues.

FDECE_2611	BKA55DRAFT_596100	FOIG_15822	FOYG_12928	FGLOB1_12591	DMATS1(FFUJ_09179)	FACUT_4333	FMEXI_13958	FSUBG_13334	FANTH_2382	FMUND_7075	FNAPI_658	FPANT_11676	FDENT_259	J7337_011243	FVEG_09966
100	65.66	67.93	67.7	64.45	66.67	64.85	65.64	65.88	65.94	65.88	65.17	65.88	65.64	65	65.94
65.66	100	78.61	77.11	73.32	74.31	73.07	72.07	73.07	74.06	75.12	74.13	72.89	73.88	79.39	74.38
67.93	78.61	100	92.74	86.38	86.36	84.94	83.33	84.04	84.93	88.29	84.07	82.9	85.95	86.67	85.2
67.7	77.11	92.74	100	83.8	87.08	85.88	83.8	84.27	85.17	88.06	84.78	84.07	86.18	84.93	85.68
64.45	73.32	86.38	83.8	100	91.17	82.39	80.33	81.5	82.34	84.31	81.03	80.56	81.97	85.22	83.53
66.67	74.31	86.36	87.08	91.17	100	86.87	83.05	83.53	84.25	87.59	84.96	84.25	85.44	86.09	86.16
64.85	73.07	84.94	85.88	82.39	86.87	100	84.04	84.74	85.68	87.79	86.15	84.51	86.62	84.64	86.16
65.64	72.07	83.33	83.8	80.33	83.05	84.04	100	94.38	95.7	86.18	82.44	82.2	83.61	82.32	83.29
65.88	73.07	84.04	84.27	81.5	83.53	84.74	94.38	100	96.42	86.89	83.37	82.67	83.84	84.06	83.77
65.94	74.06	84.93	85.17	82.34	84.25	85.68	95.7	96.42	100	86.87	83.77	83.77	84.25	84.06	84.01
65.88	75.12	88.29	88.06	84.31	87.59	87.79	86.18	86.89	86.87	100	89.98	89.04	91.38	91.07	90.97
65.17	74.13	84.07	84.78	81.03	84.96	86.15	82.44	83.37	83.77	89.98	100	93.71	91.61	89.63	90.5
65.88	72.89	82.9	84.07	80.56	84.25	84.51	82.2	82.67	83.77	89.04	93.71	100	89.98	88.18	89.31
65.64	73.88	85.95	86.18	81.97	85.44	86.62	83.61	83.84	84.25	91.38	91.61	89.98	100	92.22	93.59
65	79.39	86.67	84.93	85.22	86.09	84.64	82.32	84.06	84.06	91.07	89.63	88.18	92.22	100	98.56
65.94	74.38	85.2	85.68	83.53	86.16	86.16	83.29	83.77	84.01	90.97	90.5	89.31	93.59	98.56	100

Table S19: Sequence identities (%) of BEA1 and homologues.

**FVRRES_12556**	**FPOAC1_011500**	**BKA55DRAFT_677902**	**FOYG_12900**	**FOIG_15793**	**FMAN_12971**	**FGLOB1_2217**	**BEA1 (FFUJ_09296)**	**FPRO_13541**	**FACUT_8369**	**J7337_011263**	**FVEG_16703**	**FPCIR_12112**	**FDENT_245**	**FMUND_5269**	**FANTH_2399**	**FMEXI_4009**	**FSUBG_13646**
100	89.93	66.16	66.00	66.04	65.60	65.6	65.22	65.41	65.49	56.19	67	65.36	65.21	65.44	65.41	65.4	65.65
89.93	100	66.45	66.55	66.4	66.09	66.18	65.84	65.79	66.01	56.28	67.77	66.01	65.82	66.05	65.76	65.91	65.88
66.16	66.45	100	91.07	91.39	88.55	88.68	89.05	88.39	90.01	70.79	89.19	89.26	89.84	90.08	89.3	89.09	88.82
66.00	66.55	91.07	100	97.55	90.18	90.49	90.62	90.33	91.80	72.70	90.45	90.53	91.15	91.61	90.55	90.87	90.15
66.04	66.40	91.39	97.55	100	90.47	90.66	90.78	90.59	92.38	73.14	90.95	90.79	91.35	92	90.87	90.88	90.45
65.60	66.09	88.55	90.18	90.47	100	95.09	94.83	94.64	90.52	70.9	90.05	89.45	89.64	90.11	89.05	88.96	88.27
65.60	66.18	88.68	90.49	90.66	95.09	100	95.09	95.76	90.42	70.07	89.95	89.13	89.48	89.85	89.12	89.06	88.6
65.22	65.84	89.05	90.62	90.78	94.83	95.09	100	95.76	90.9	70.94	90.22	89.25	89.86	90.23	89.3	89.47	88.78
65.41	65.79	88.39	90.33	90.59	94.64	95.76	95.76	100	90.33	70.07	90.09	88.9	89.26	89.79	88.99	88.8	88.34
65.49	66.01	90.01	91.80	92.38	90.52	90.42	90.9	90.33	100	72.03	91.35	91.13	91.73	91.99	90.8	90.52	90.12
56.19	56.28	70.79	72.70	73.14	70.9	70.07	70.94	70.07	72.03	100	97.68	73.72	74.06	71.88	69.88	71.36	70.69
67.00	67.77	89.19	90.45	90.95	90.05	89.95	90.22	90.09	91.35	97.68	100	92.07	92.56	90.9	89.5	89.73	89.46
65.36	66.01	89.26	90.53	90.79	89.45	89.13	89.25	88.9	91.13	73.72	92.07	100	95.19	90.94	90.01	89.89	89.67
65.21	65.82	89.84	91.15	91.35	89.64	89.48	89.86	89.26	91.73	74.06	92.56	95.19	100	91.54	90.53	90.19	89.94
65.44	66.05	90.08	91.61	92.00	90.11	89.85	90.23	89.79	91.99	71.88	90.9	90.94	91.54	100	90.87	90.78	90.55
65.41	65.76	89.30	90.55	90.87	89.05	89.12	89.3	88.99	90.8	69.88	89.5	90.01	90.53	90.87	100	93.9	93.47
65.40	65.91	89.09	90.87	90.88	88.96	89.06	89.47	88.8	90.52	71.36	89.73	89.89	90.19	90.78	93.9	100	93.73
65.65	65.88	88.82	90.15	90.45	88.27	88.6	88.78	88.34	90.12	70.69	89.46	89.67	89.94	90.55	93.47	93.73	100

Supplementary Figures.

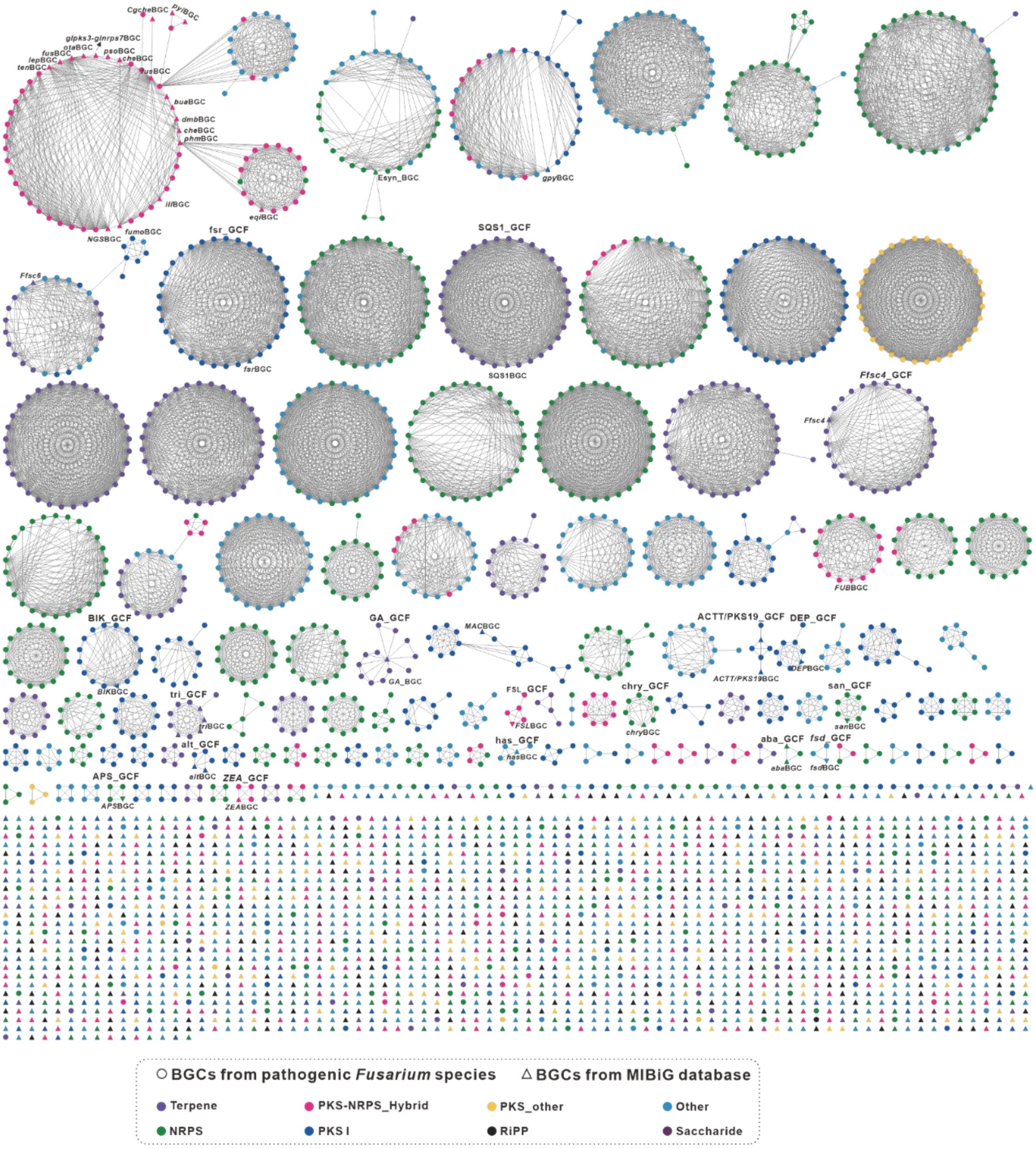

Figure S1: GCF network of 1733 predicted biosyntheic gene cluster (BGC) from 35 pathogenic *Fusarium* species caculated by BiG-SCAPE pipeline and visualised with Cytoscape.

-

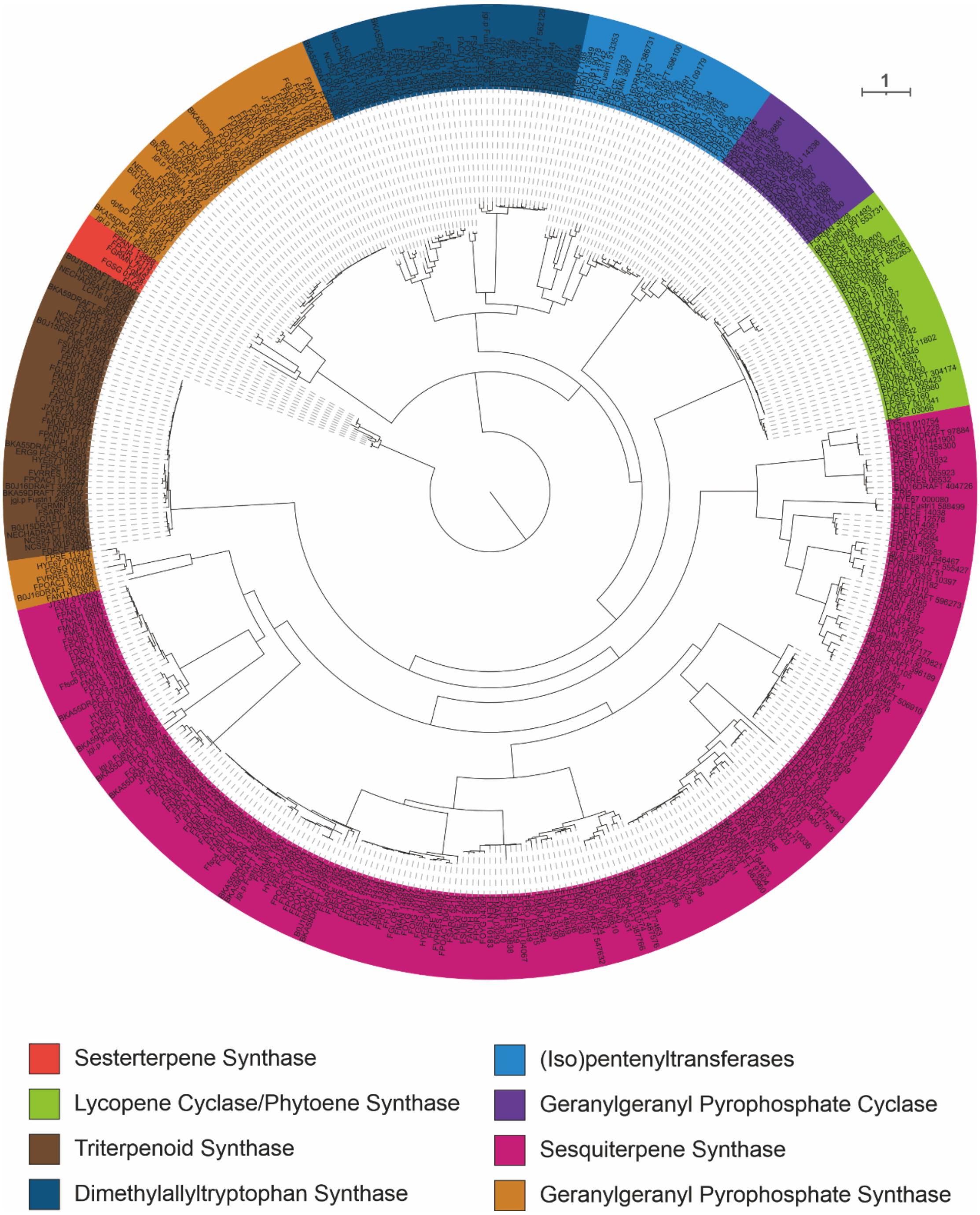

Figure S2: Cluster analysis of terpenoid synthase and their homologues based on phylogenetic tree.

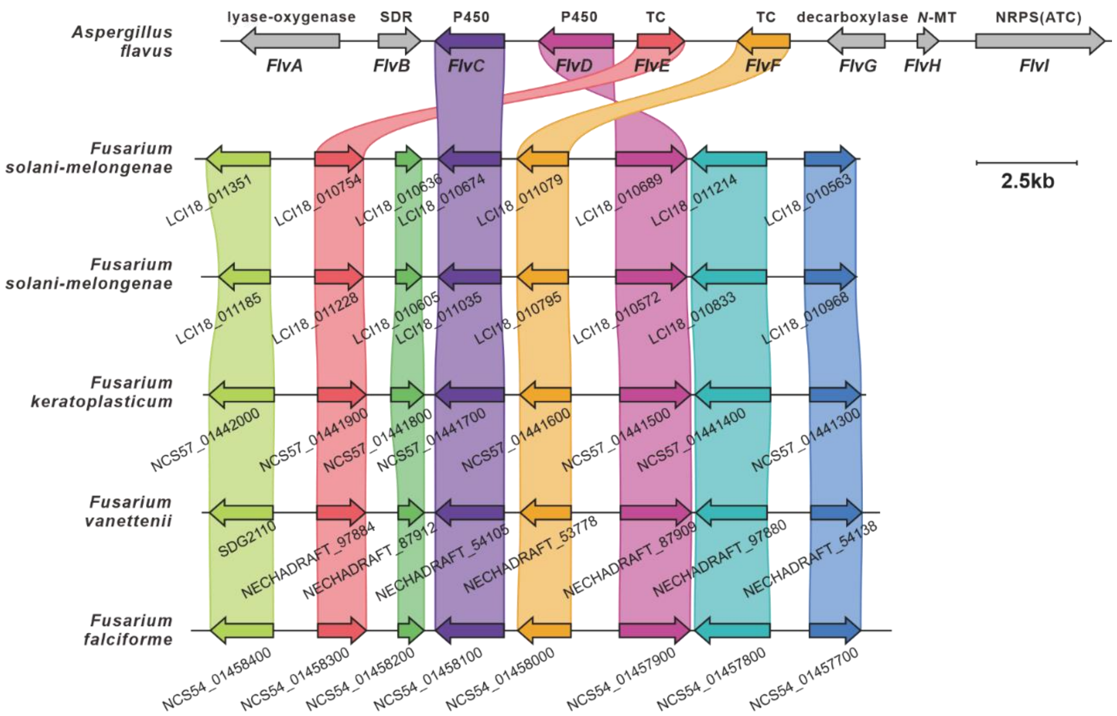

Figure S3: Comparison of *Flv*BGCs from different species.

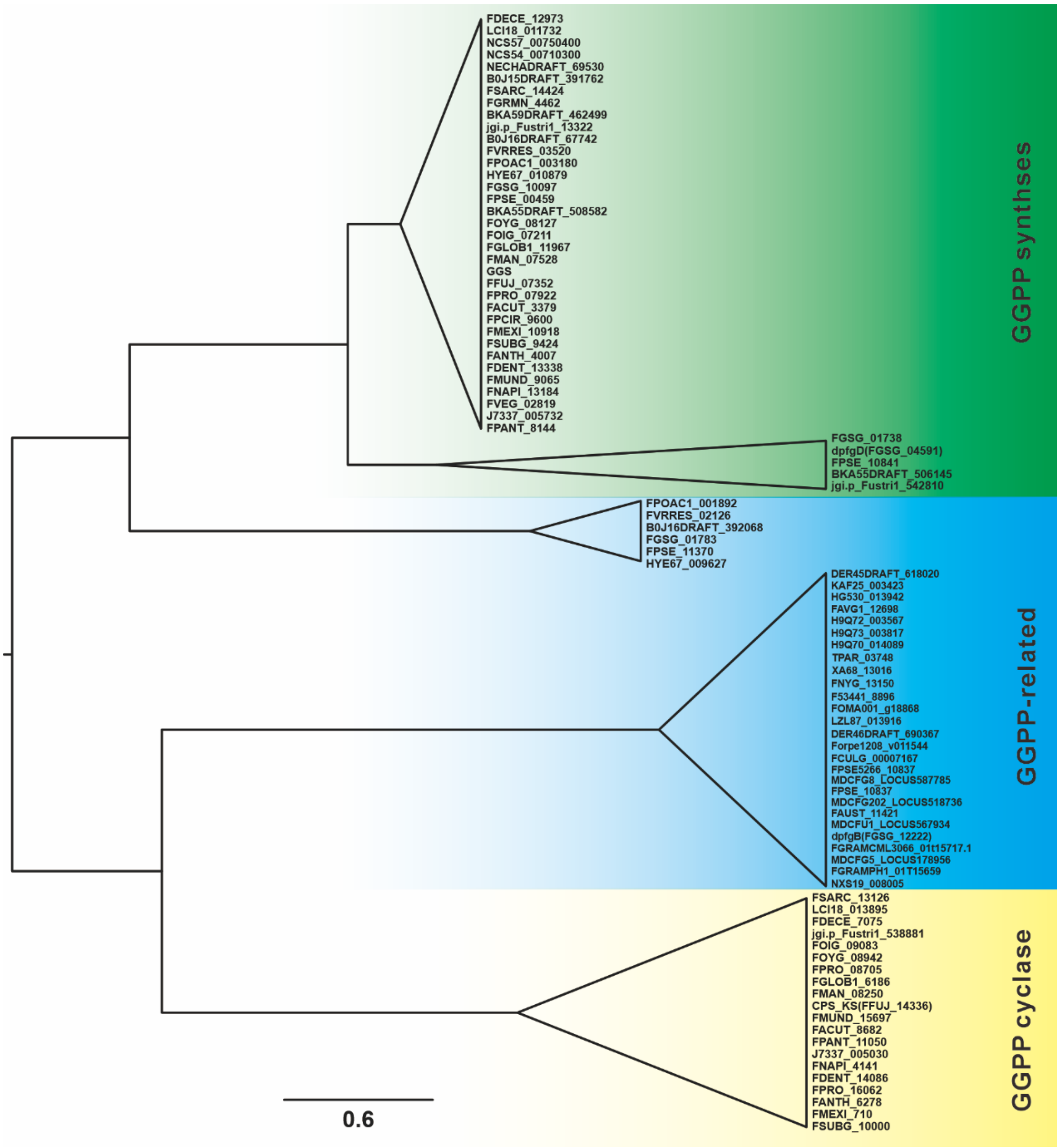

Figure S4: Phylogenetic tree-based cluster analysis of GGPP-related enzymes.

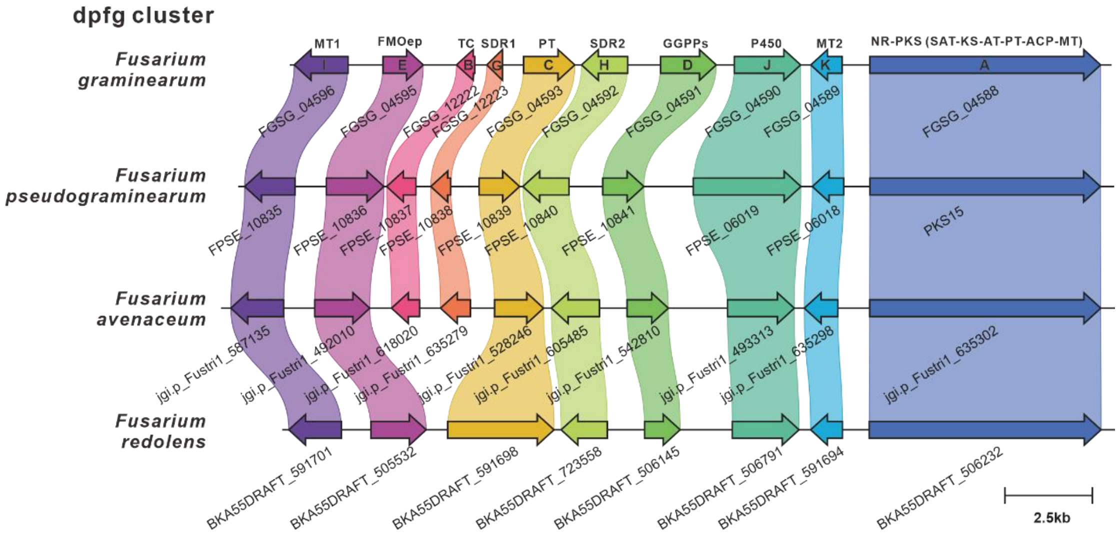

Figure S5: Comparison of *dpfg*BGCs from different pathogenic *Fusarium* species.

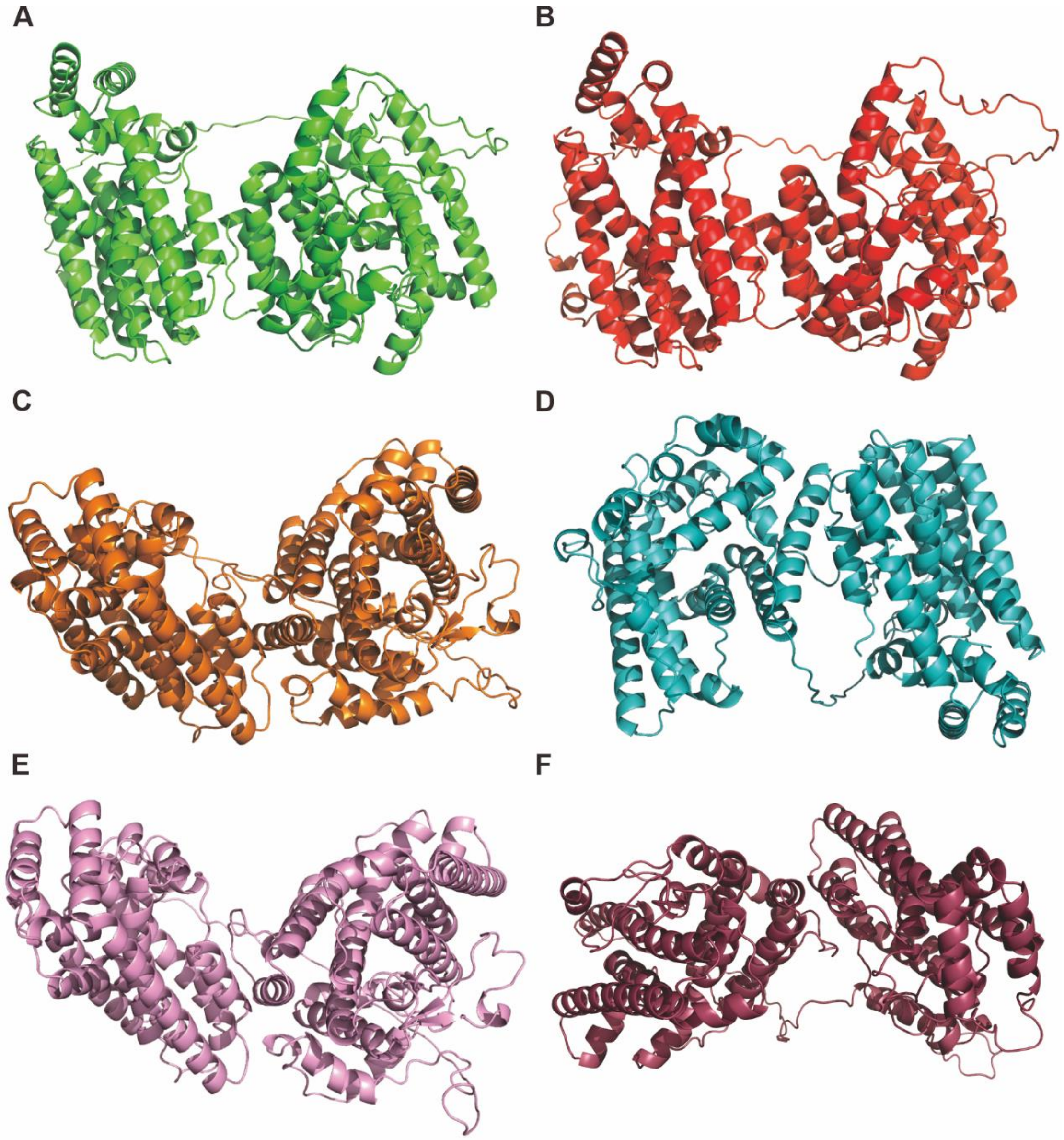

Figure S6: The predicted three-dimensional structure of protein of FgMS (A), FGSG_01738 (B), FGRMN_7913 (C), FPCIR_12113 (D), FPANT_13888 (E) and FoFs(F) predicted by Alphafold.

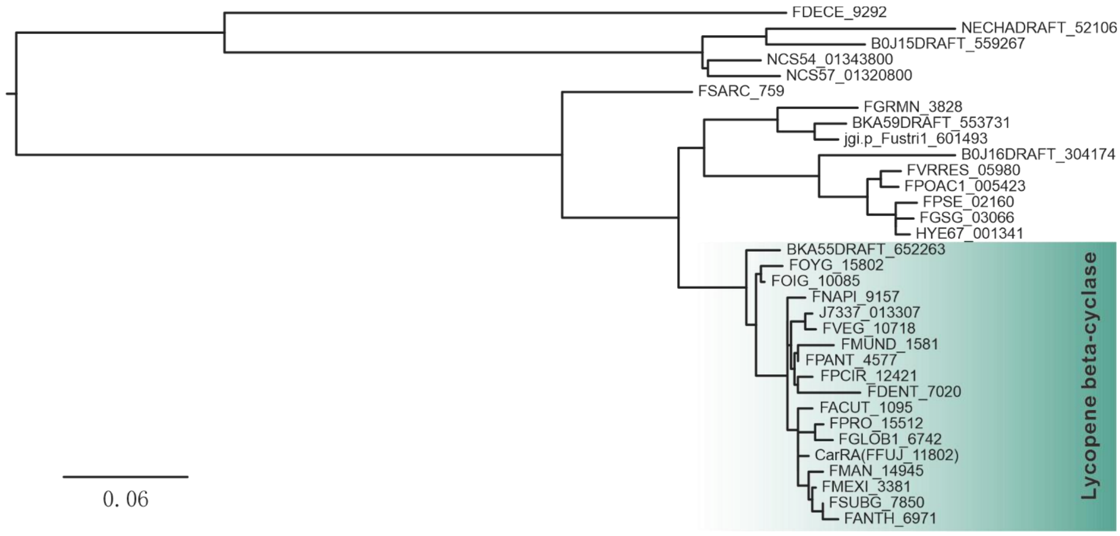

Figure S7: Phylogenetic tree-based cluster analysis of CarRA and its homologues.

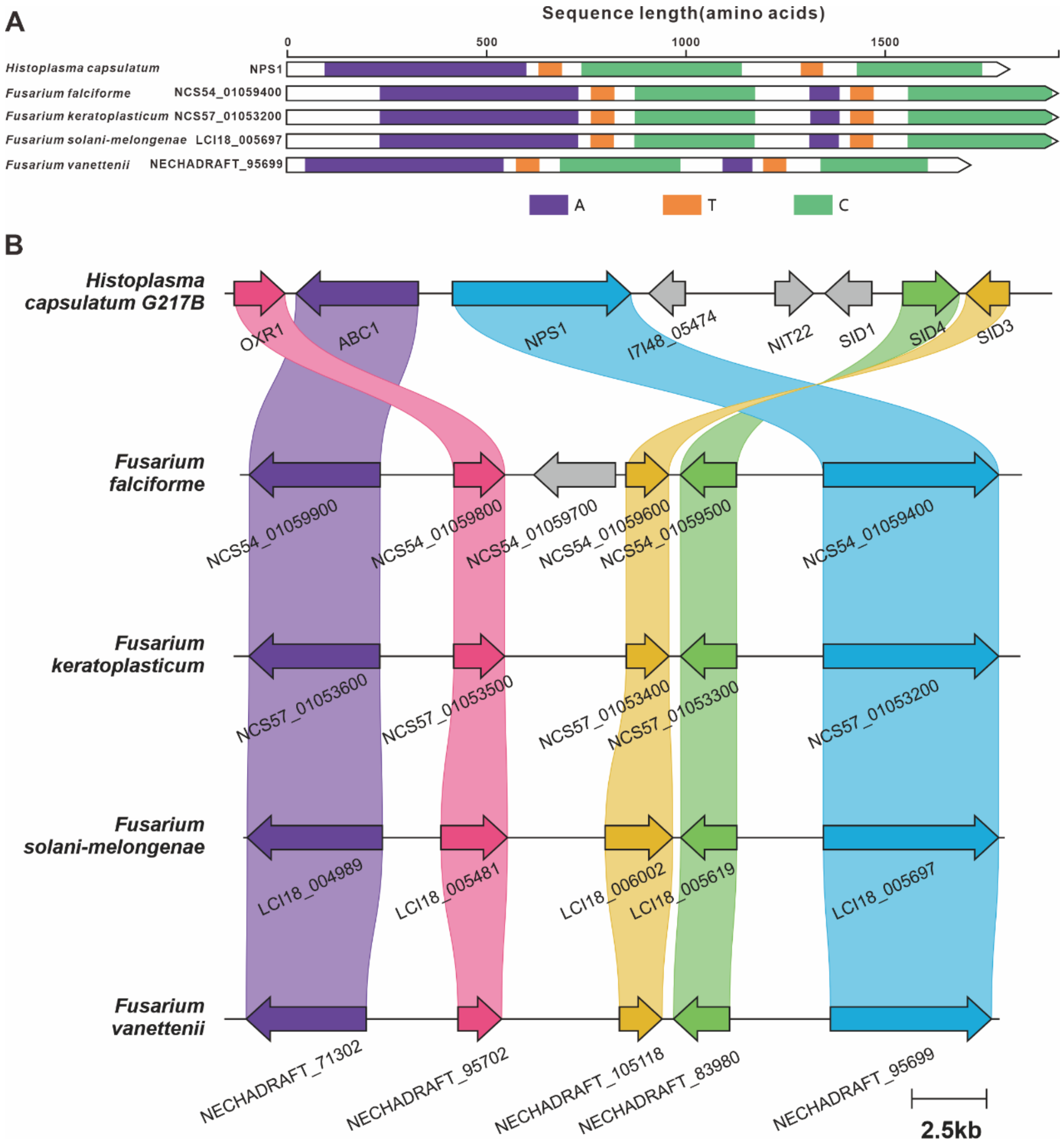

Figure S8: Domain comparison of NPS1 and its homologues(A), comparison of the BGCs containing *nps1* from different species(B).

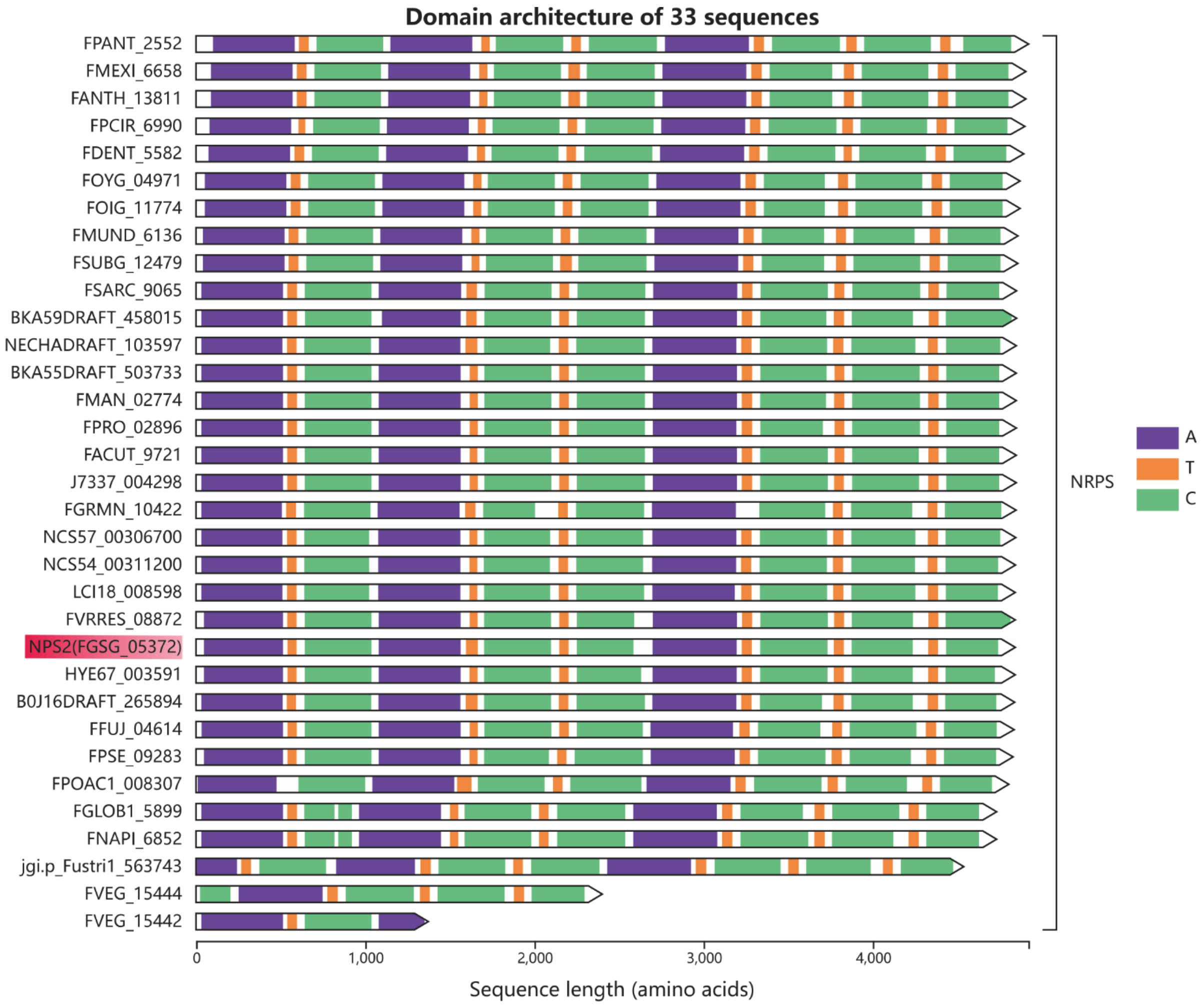

Figure S9: Domain comparison of NPS2 and its homologues.

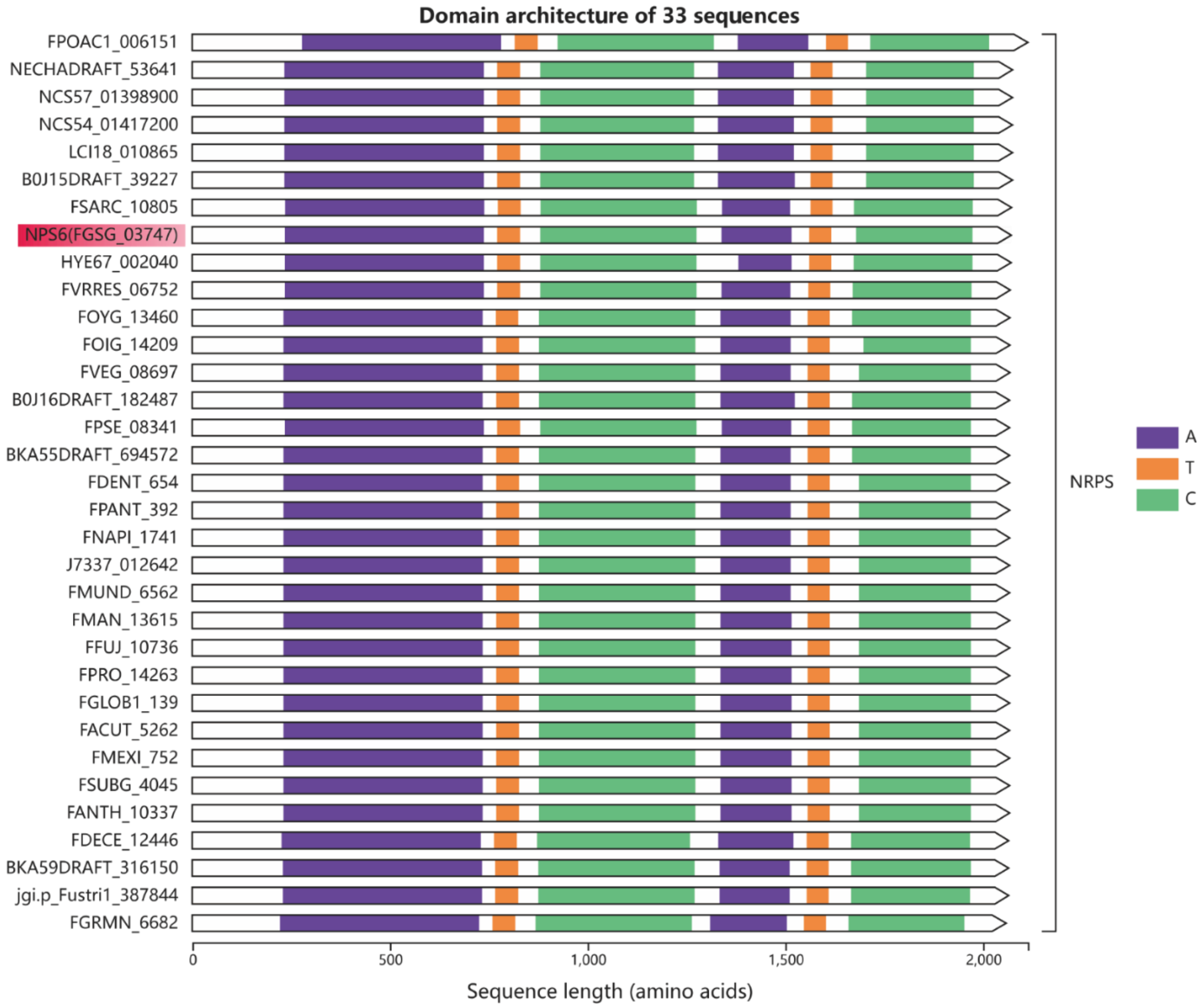

Figure S10: Domain comparison of NPS6 and its homologues.

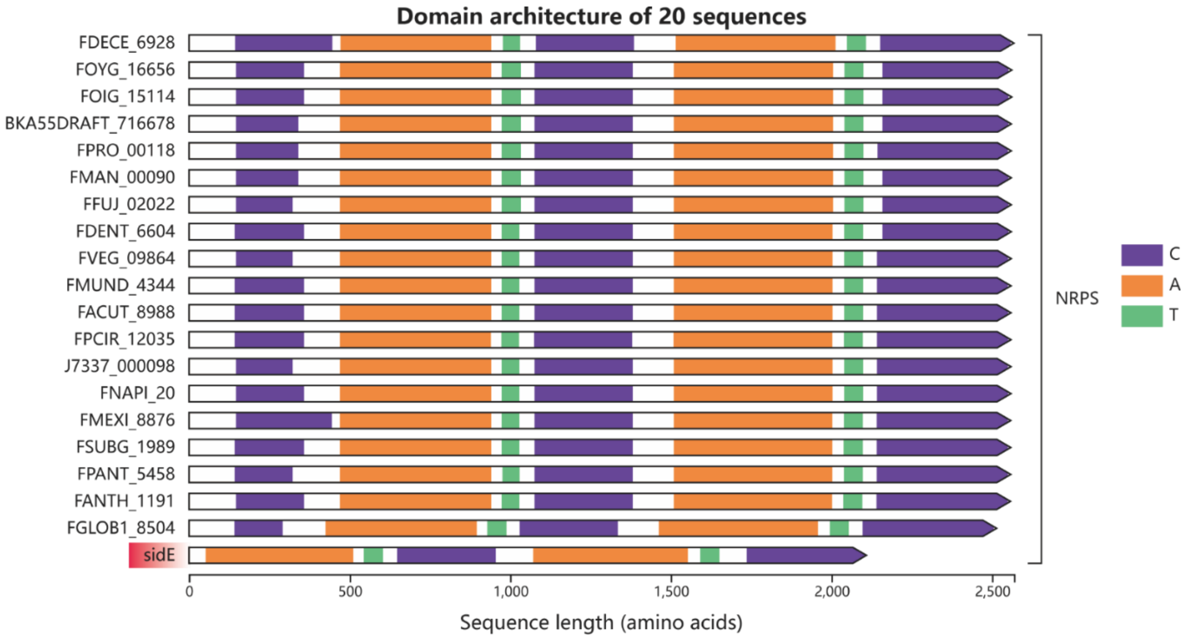

Figure S11: Domain comparison of SidE and its homologues.

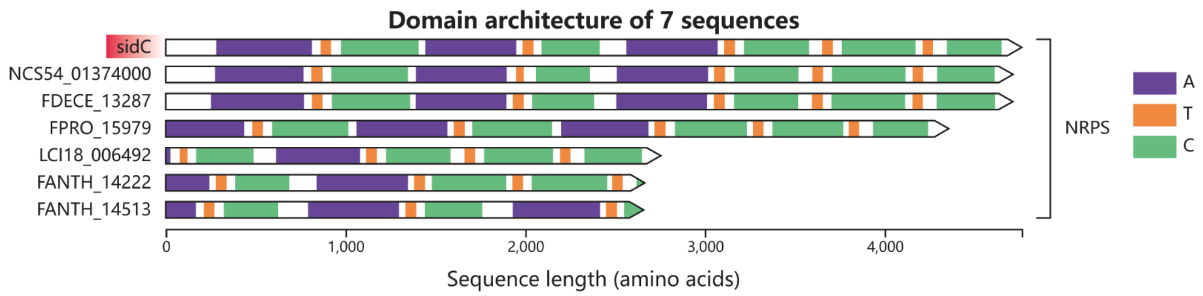

Figure S12: Domain comparison of SidC and its homologues.

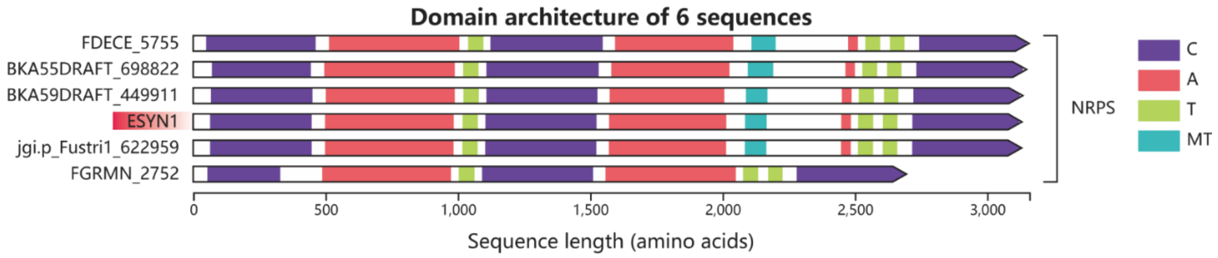

Figure S13: Domain comparison of ESYN1 and its homologues.

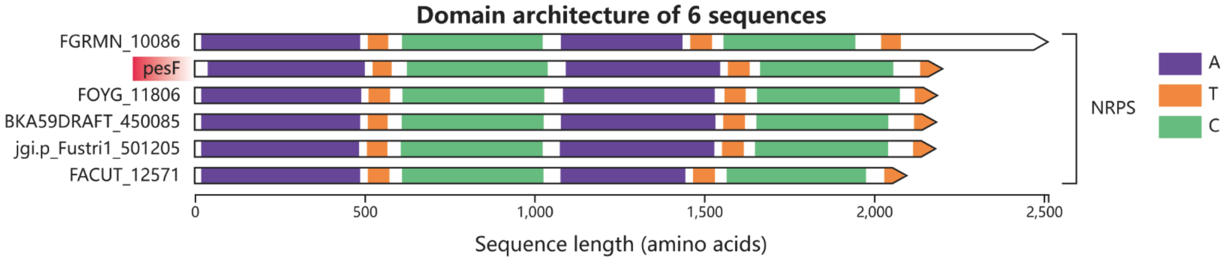

Figure S14: Domain comparison of PesF and its homologues.

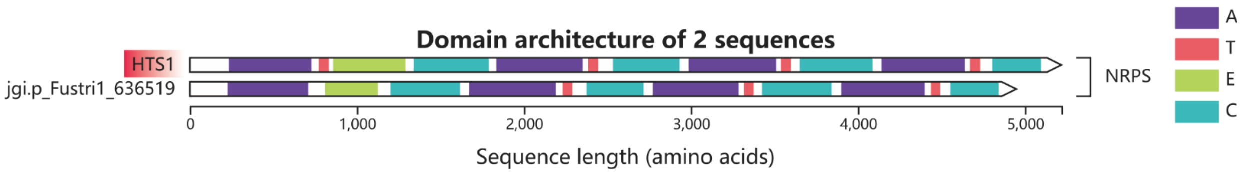

Figure S15: Domain comparison of HTS1 and its homologues.

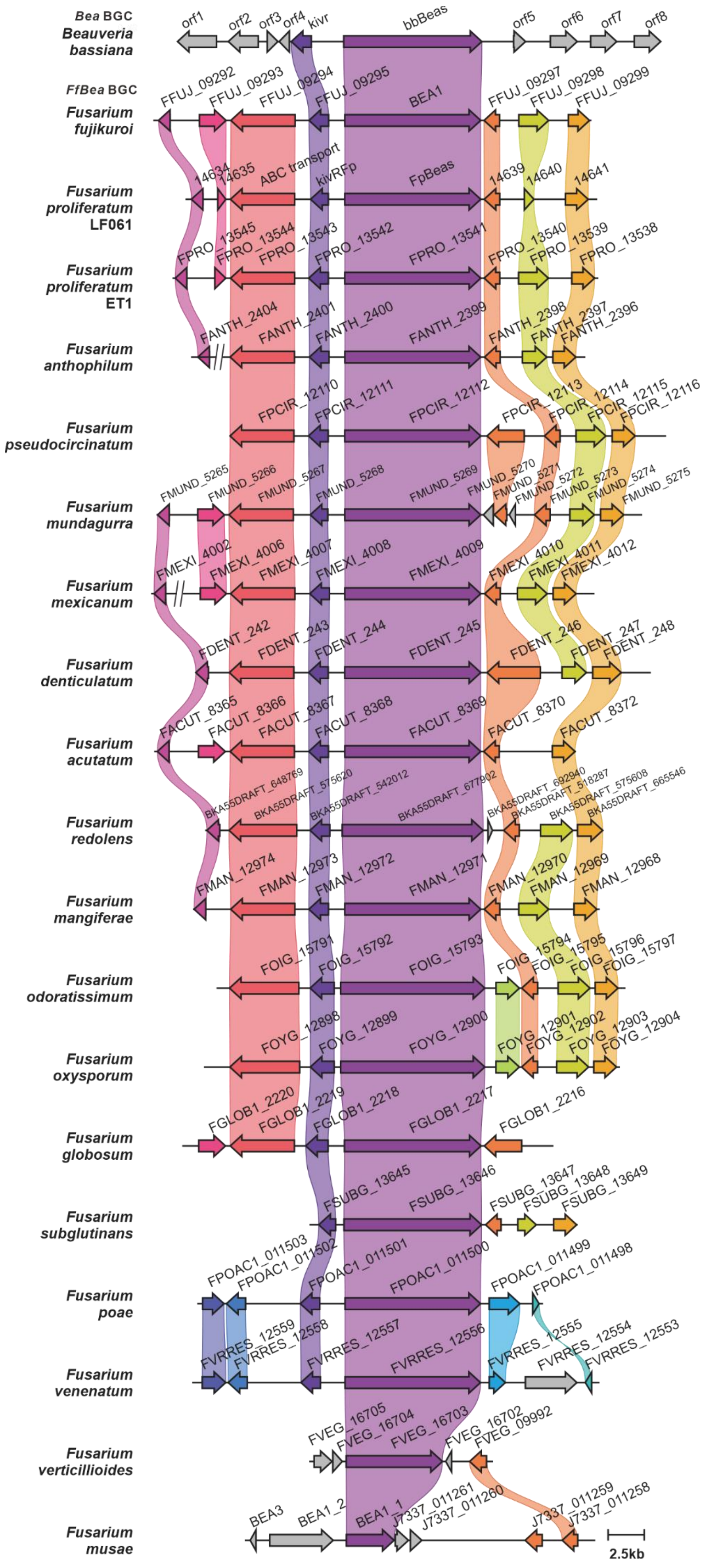

Figure S16: Comparison of *Bea*BGCs from different pathogenic fungi.

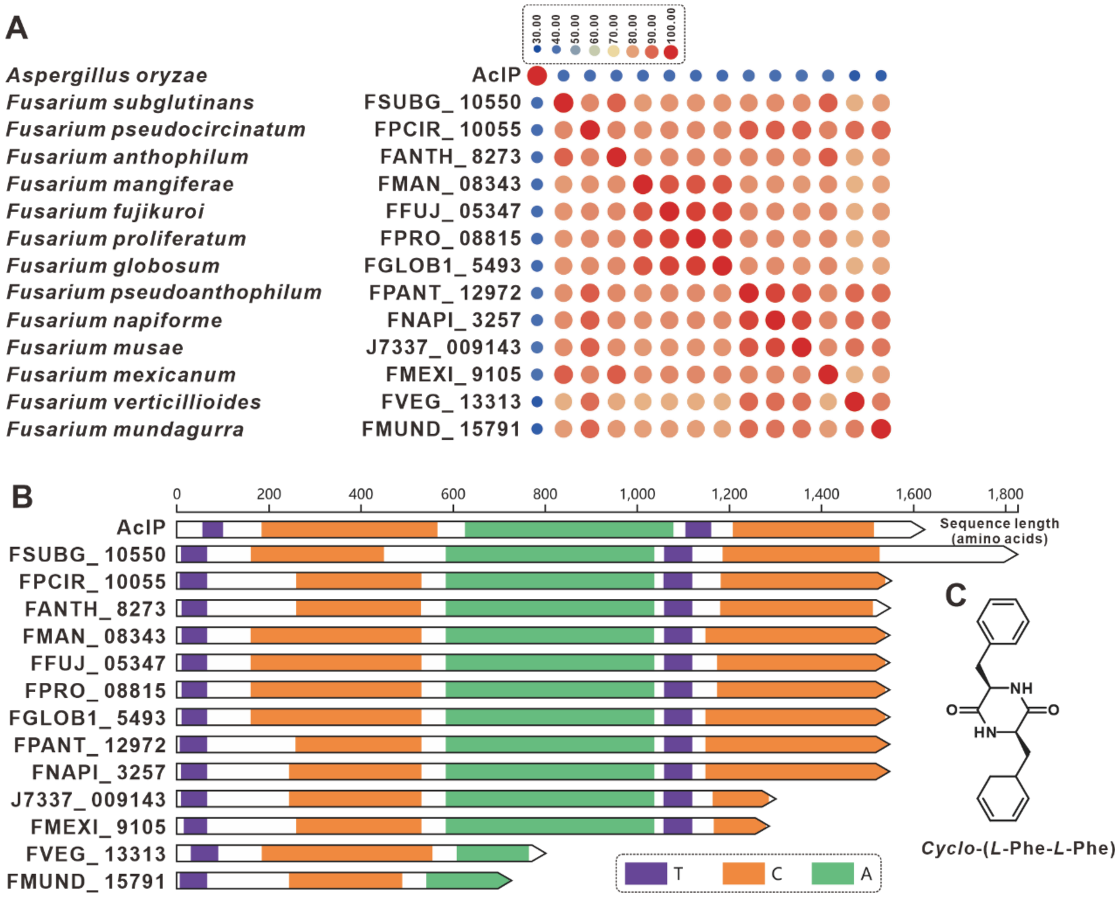

Figure S17: Comparative analysis of AclP and its homologues. Comparison of the amino acid sequence identity of AclP and its homologues(A), comparison of the structural domains of AclP and its homologues(B), structure of cyclo-(L-Phe-L-Phe)(C).

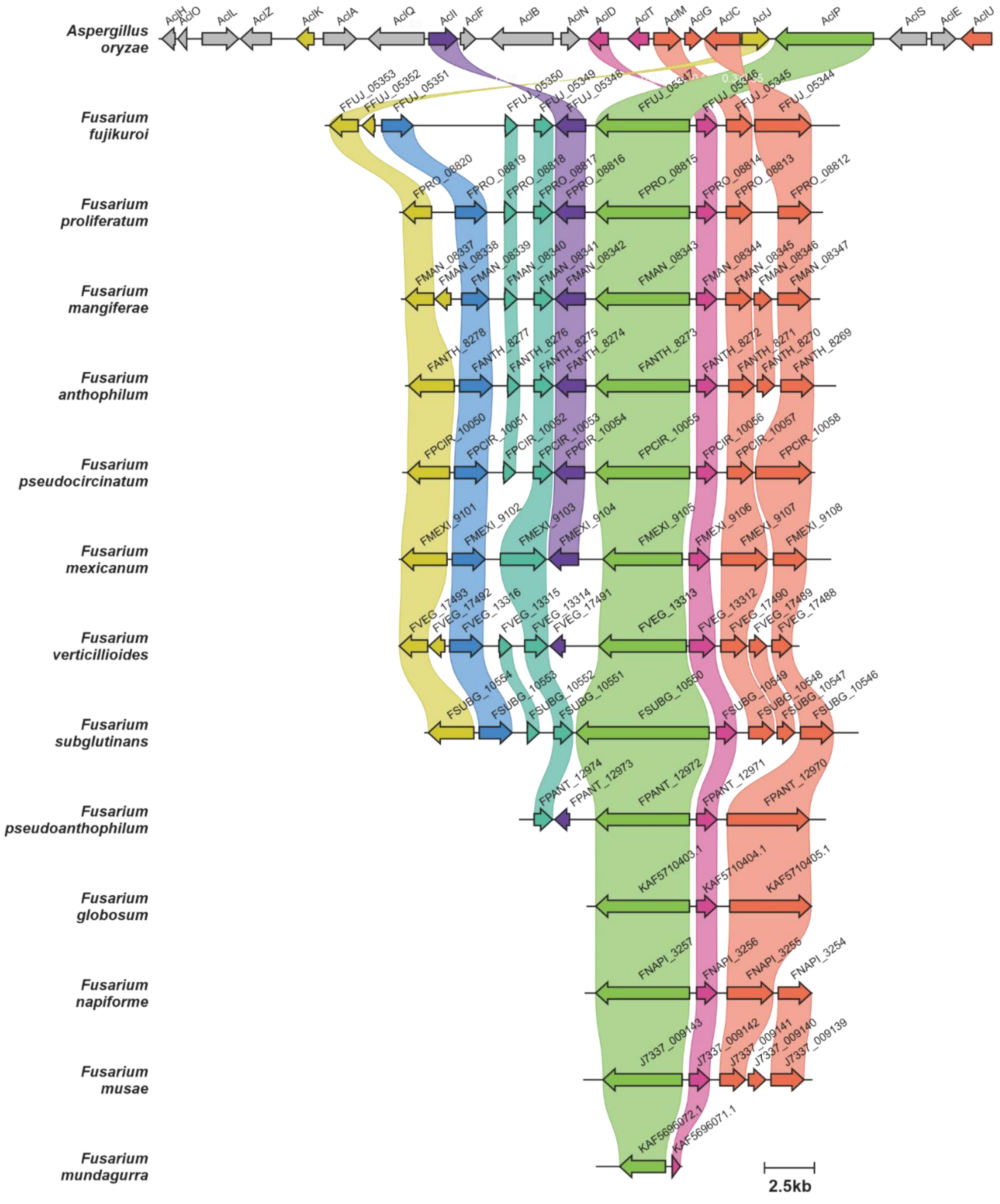

Figure S18: Comparison of *Acl*BGCs from different species.

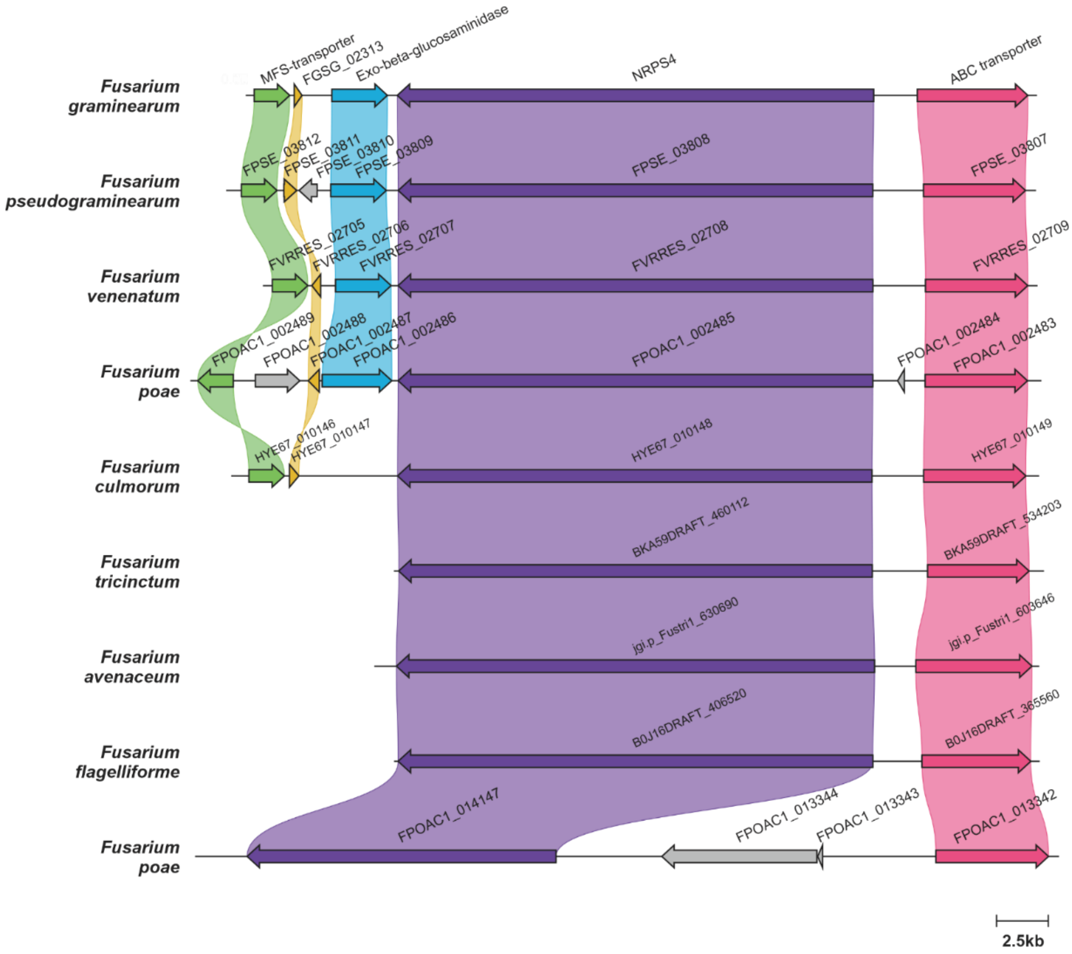

Figure S19: Comparison of the BGCs containing *nrps4* from different *Fusarium* species.

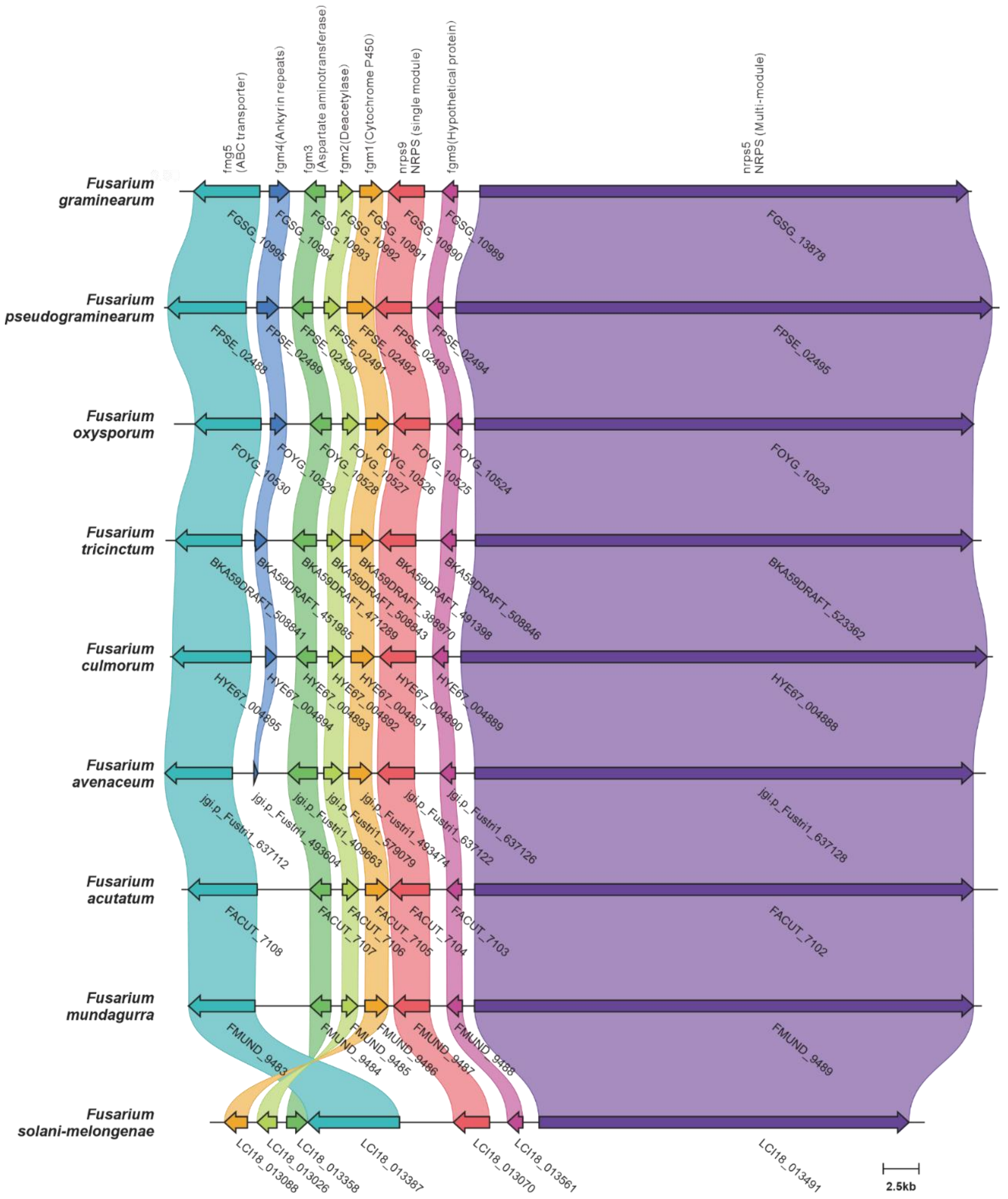

Figure S20: Comparison of the BGCs containing *nrps5* from different *Fusarium* species.

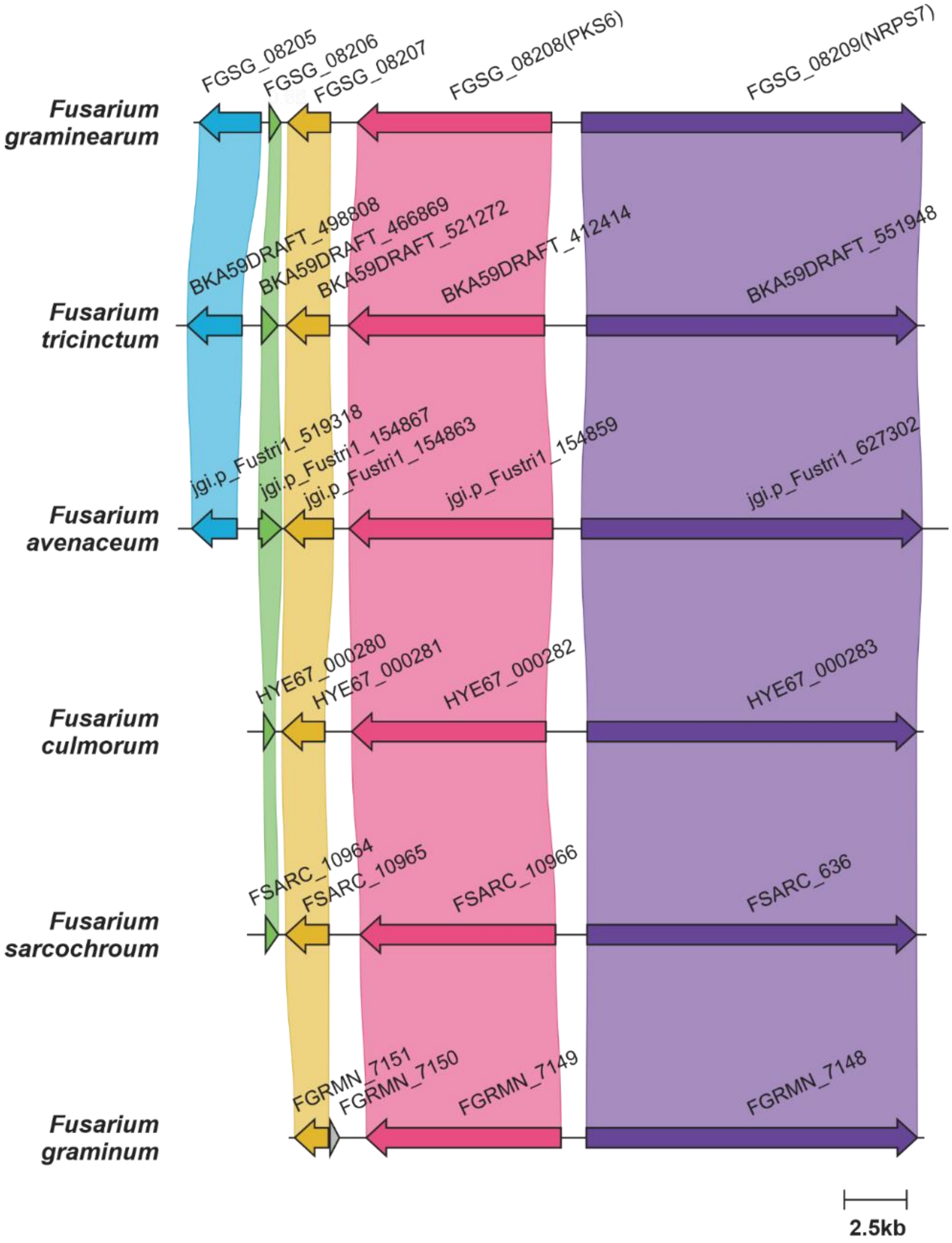

Figure S21: Comparison of the BGCs containing *nrps7* and PKS6 from different *Fusarium* species.

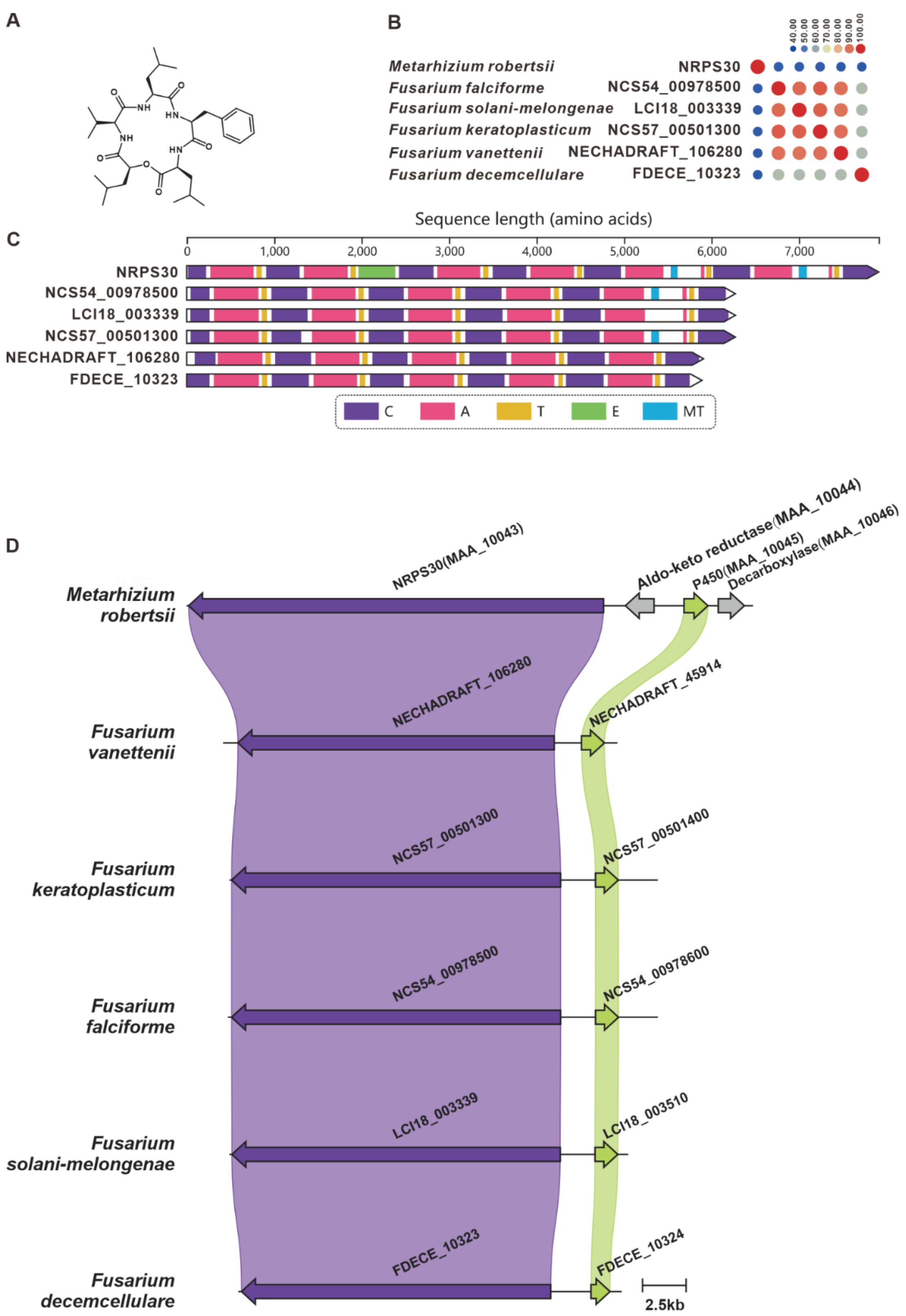

Figure S22: Structures of sansalvamide (A), comparison of the amino acid sequence identity of NRPS30 and its homologues (B), domain comparison of NRPS30 and its homologues (C), comparison of the BGC for aurofusarin and its similar BGCs (D).

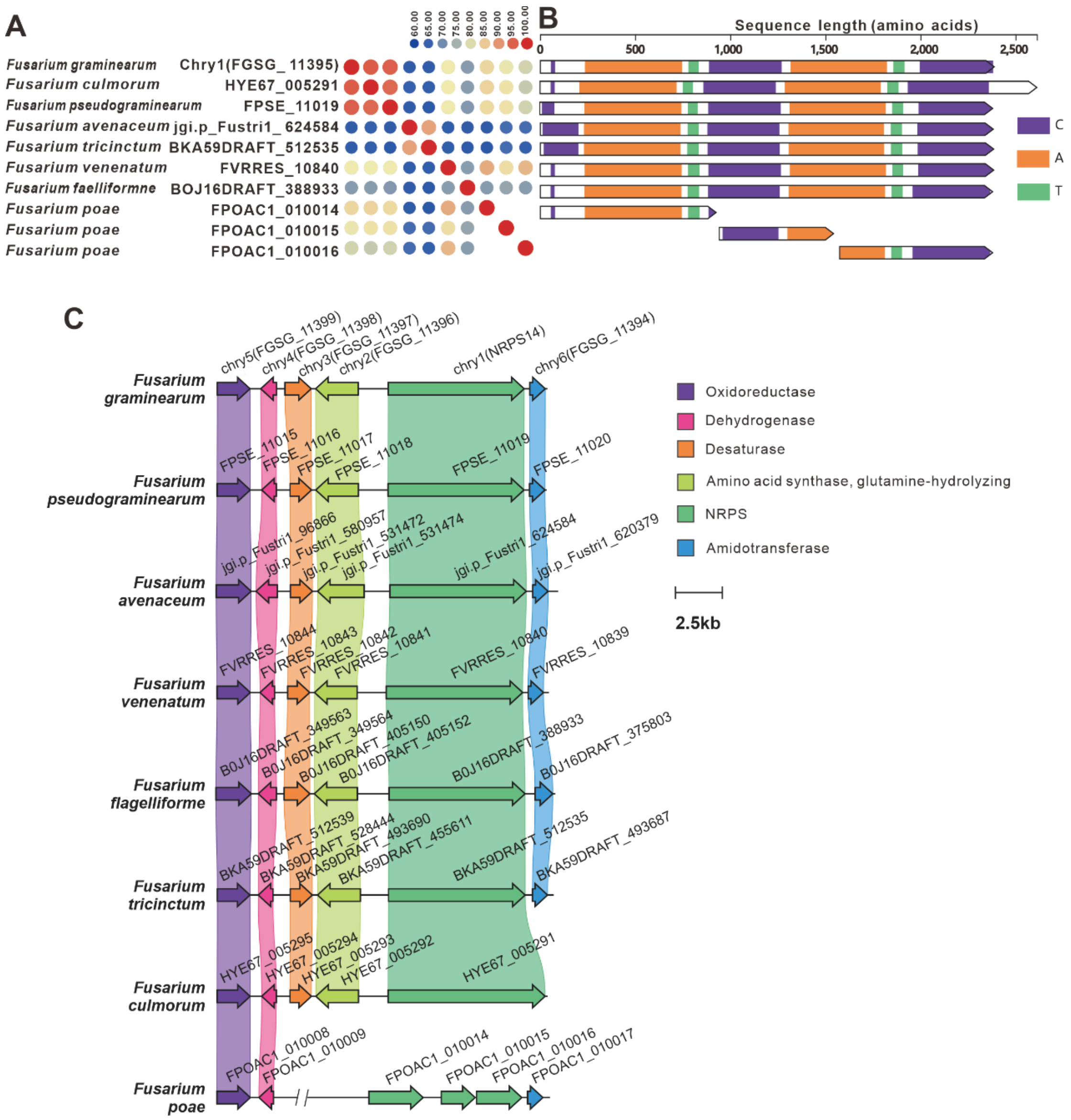

Figure S23: Comparison of the amino acid sequence identity of Chry1 and its homologues (A), domain comparison of Chry1 and its homologues (B), comparison of *chry*BGCs from different *Fusarium* species.

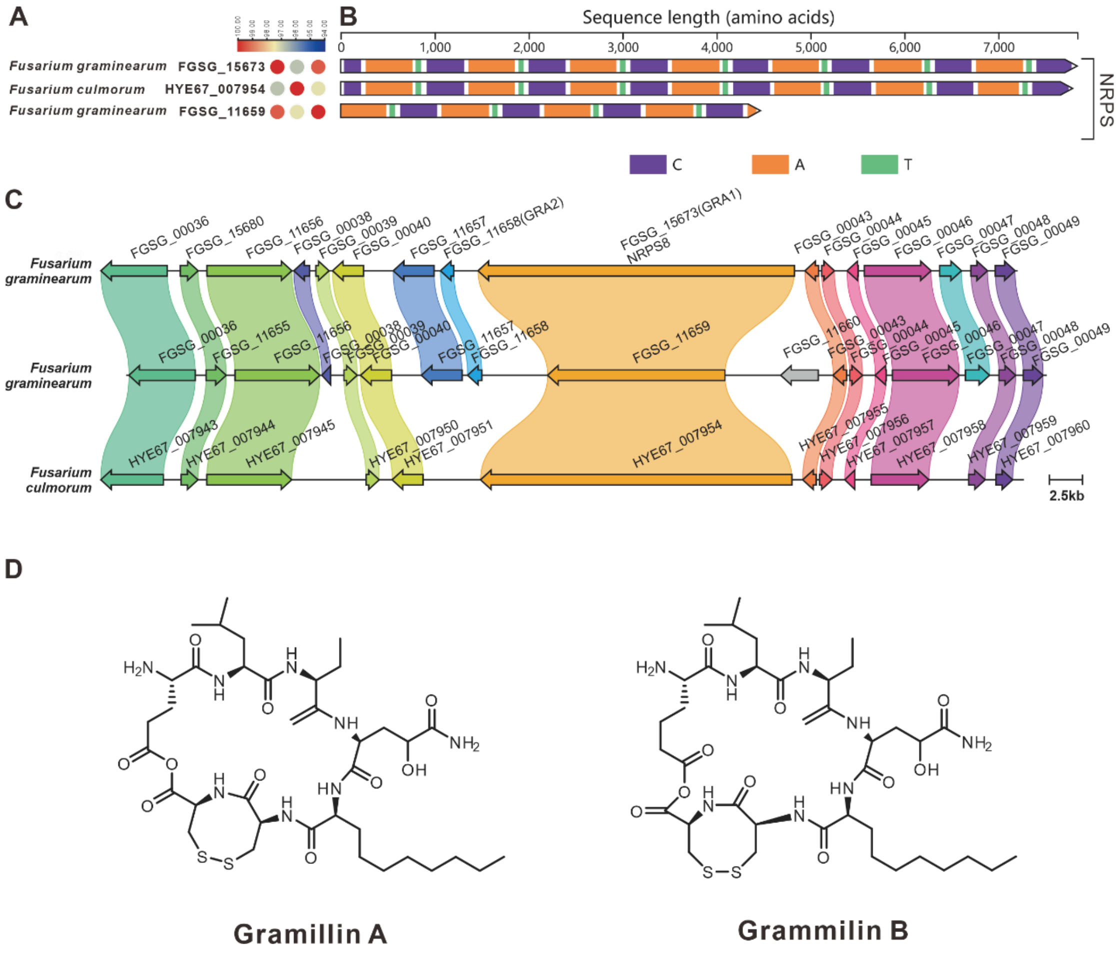

Figure S24: Comparison of the amino acid sequence identity of GRA1 and its homologues (A), domain comparison of GRA1 and its homologues (B), comparison of the BGC for Gramillin A, Gramillin B and its similar BGCs (C), structure of Gramillin A and Gramillin B (D).

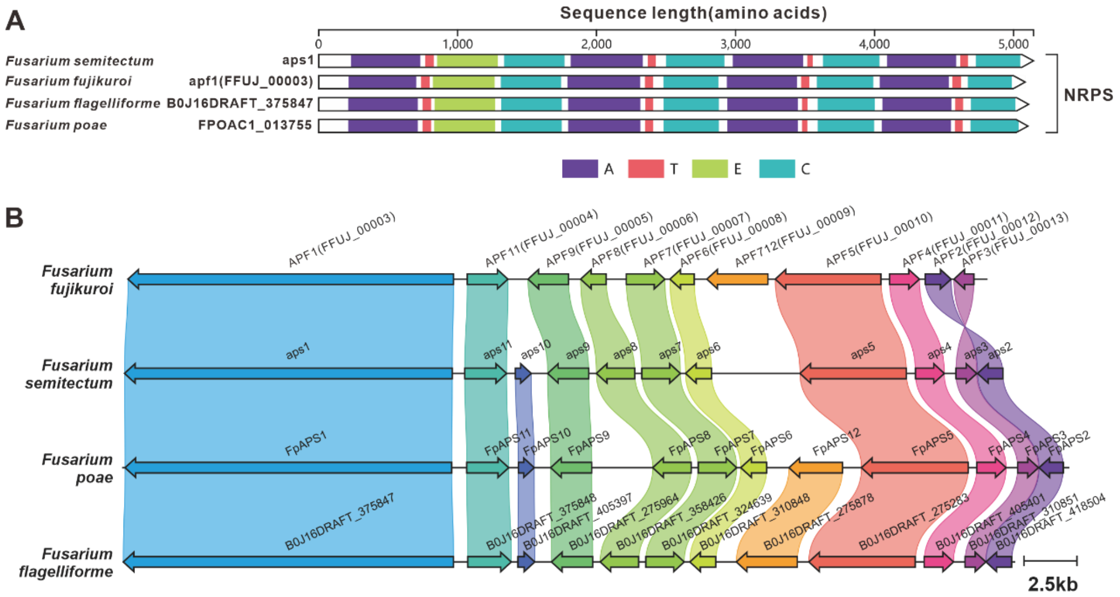

Figure S25: Domain comparison of apf1 and its homologues (A), comparison of *apf*BGCs from different *Fusarium* species (B).

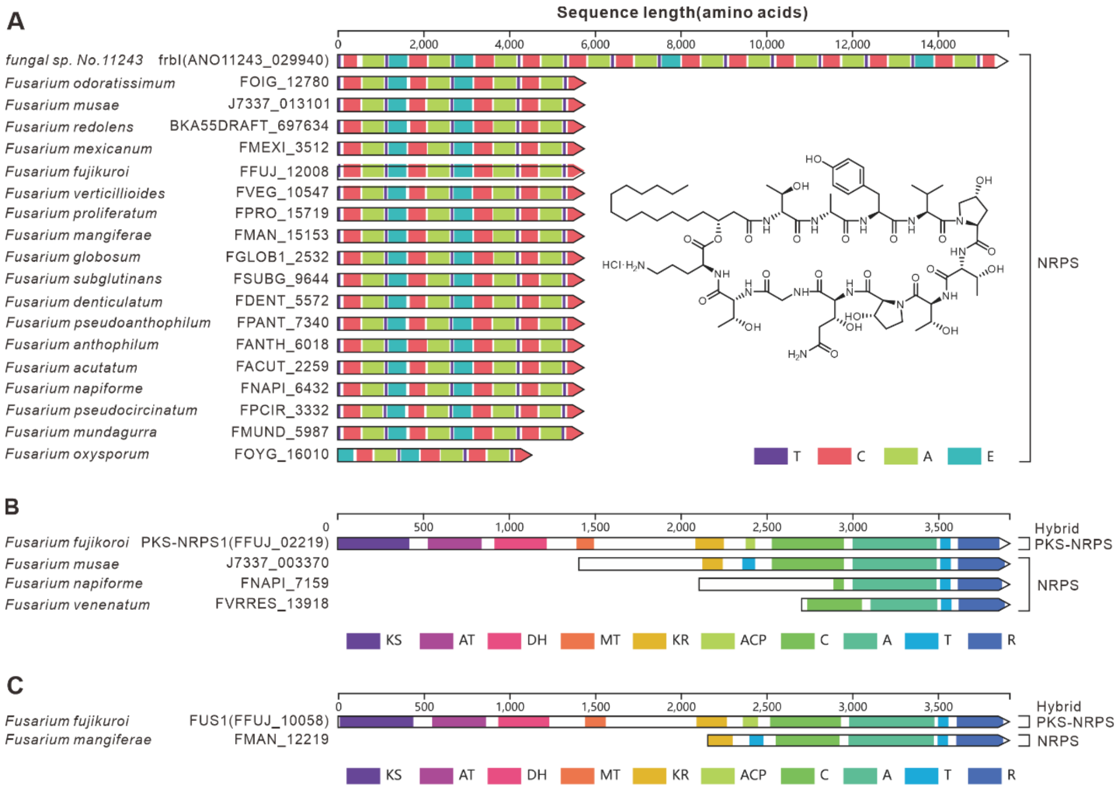

Figure S26: Domain comparison of FrbI and its homologues (A), domain comparison of PKS-NRPS1 and its homologues (B), domain comparison of FUS1 and its homologues (C).

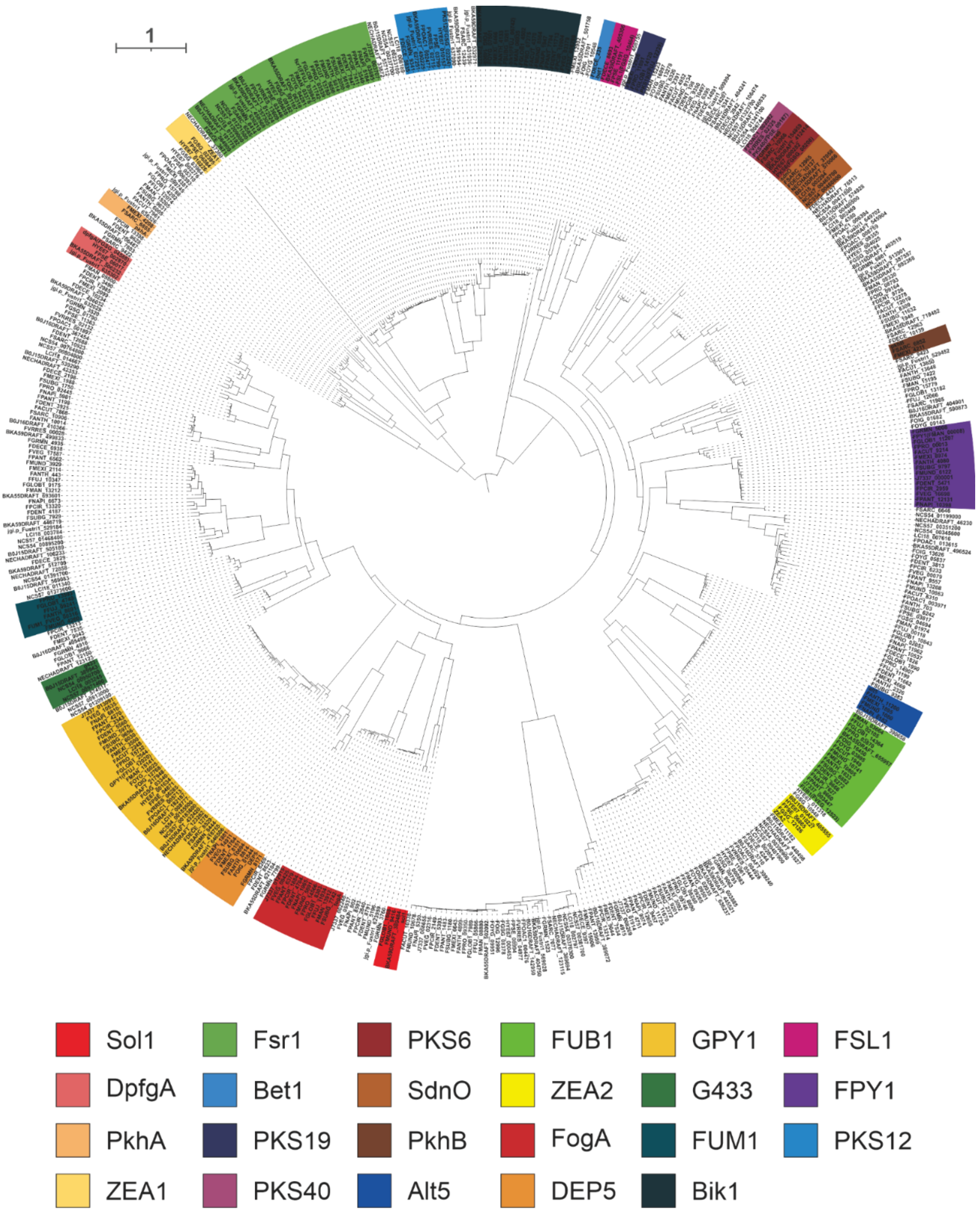

Figure S27: Phylogenetic tree-based cluster analysis of PKS-related enzymes.

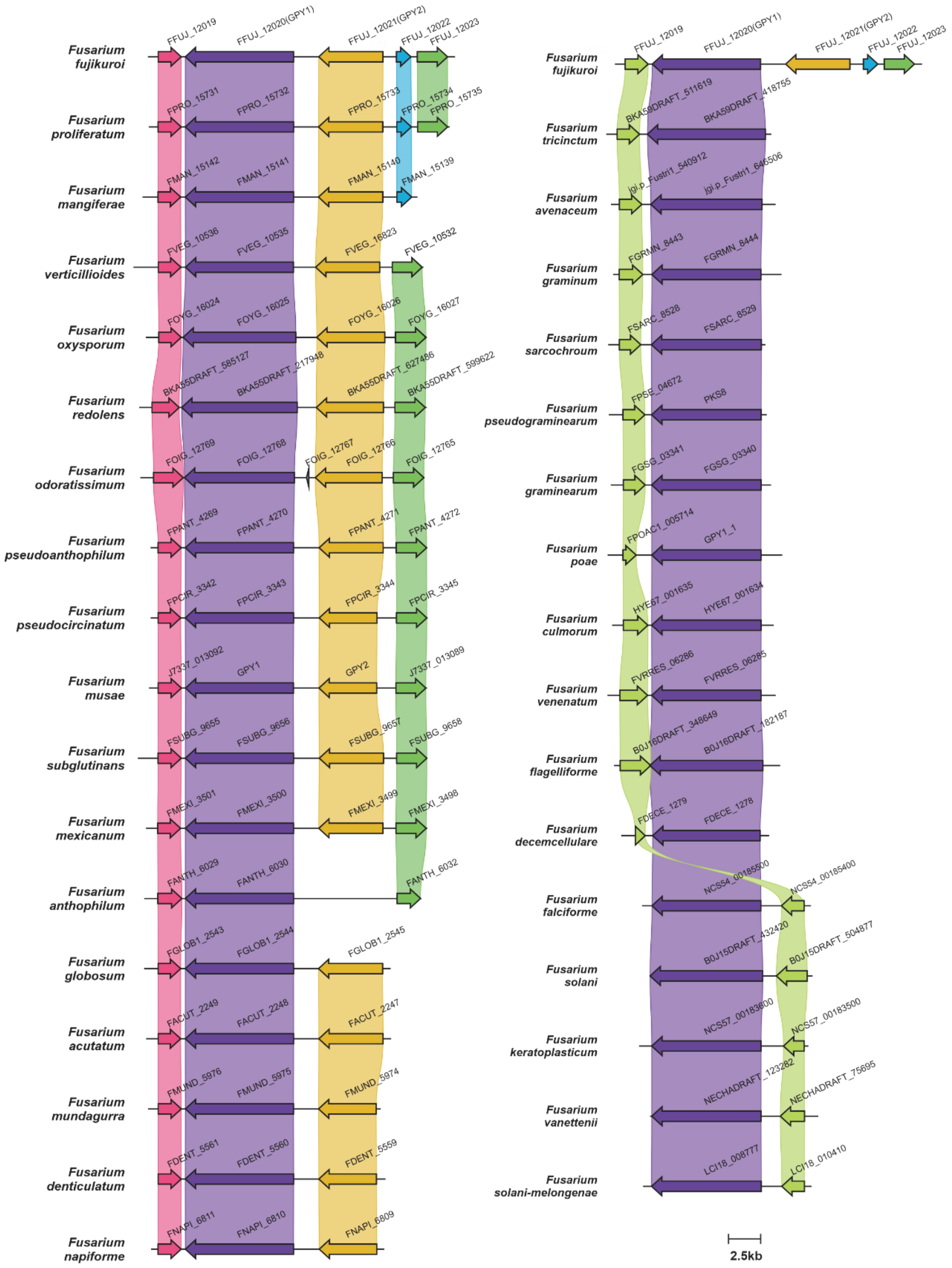

Figure S28: Comparison of *gpy*BGCs from different *Fusarium* species.

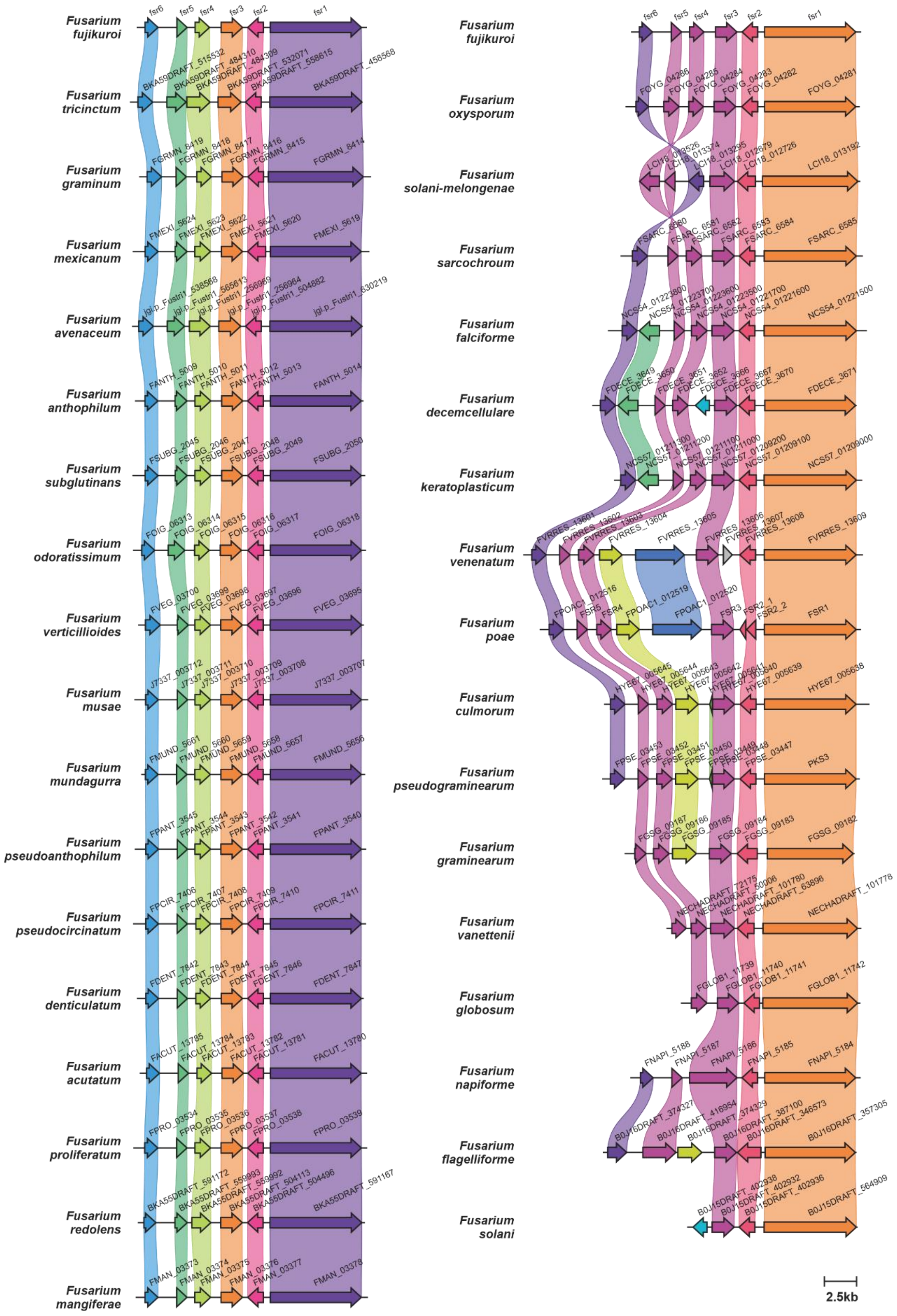

Figure S29: Comparison of *fsr*BGCs from different *Fusarium* species.

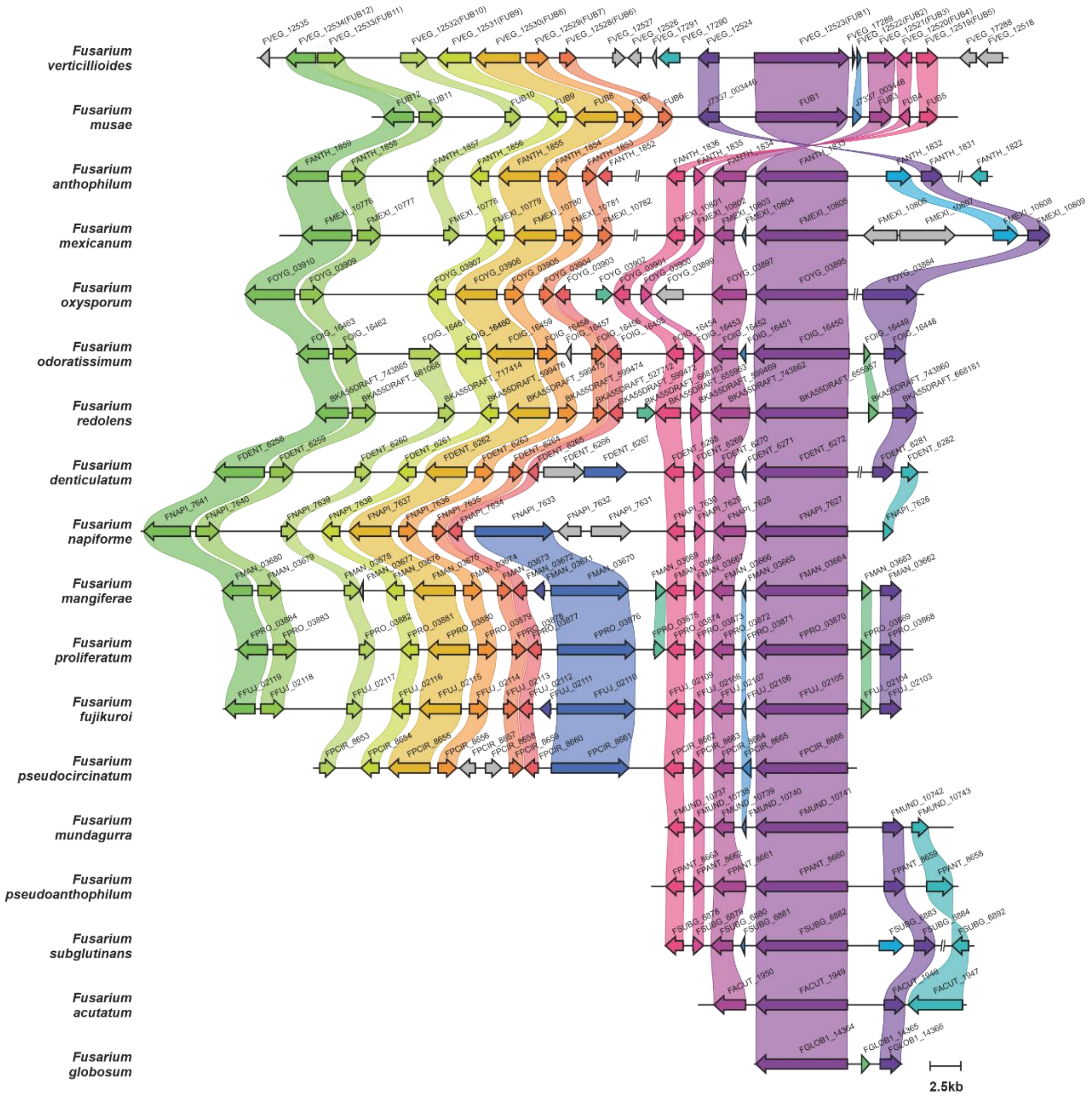

Figure S30: Comparison of *FUB*BGCs from different *Fusarium* species.

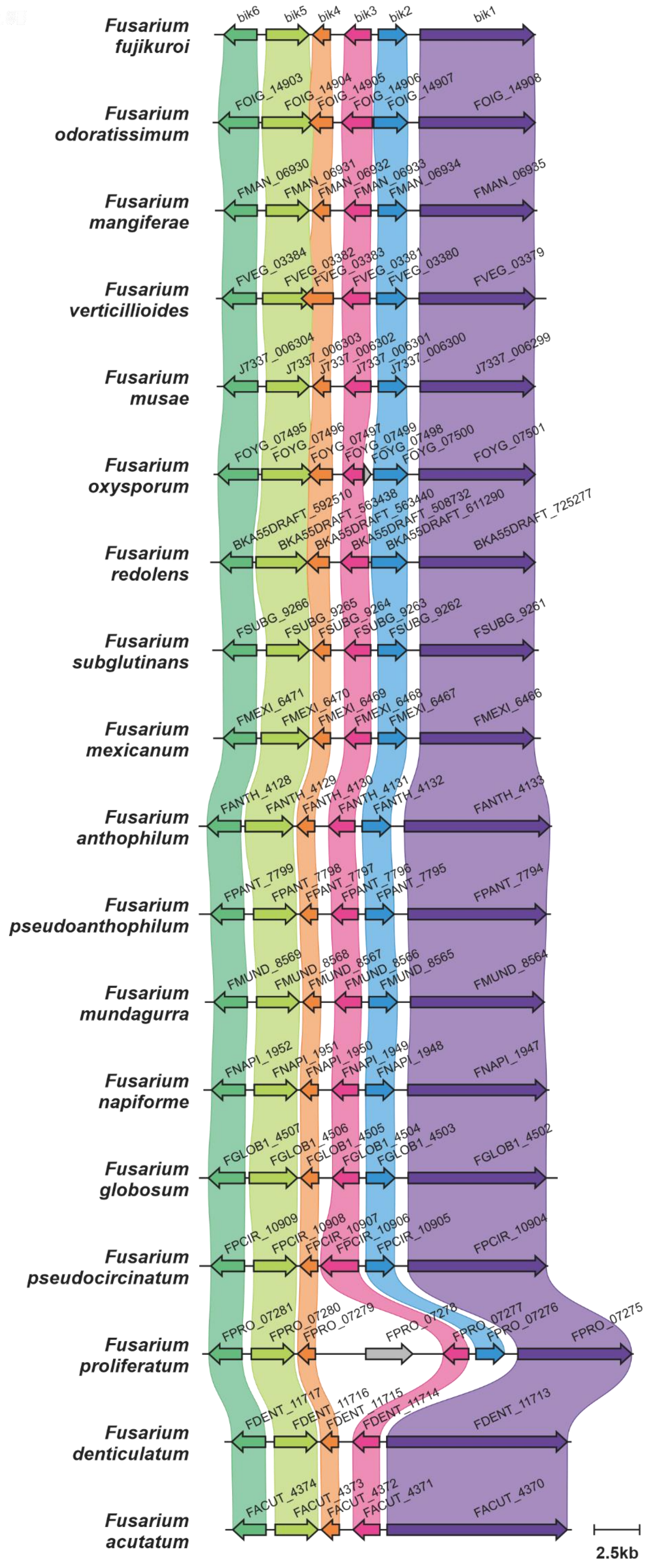

Figure S31: Comparison of *bik*BGCs from different *Fusarium* species.

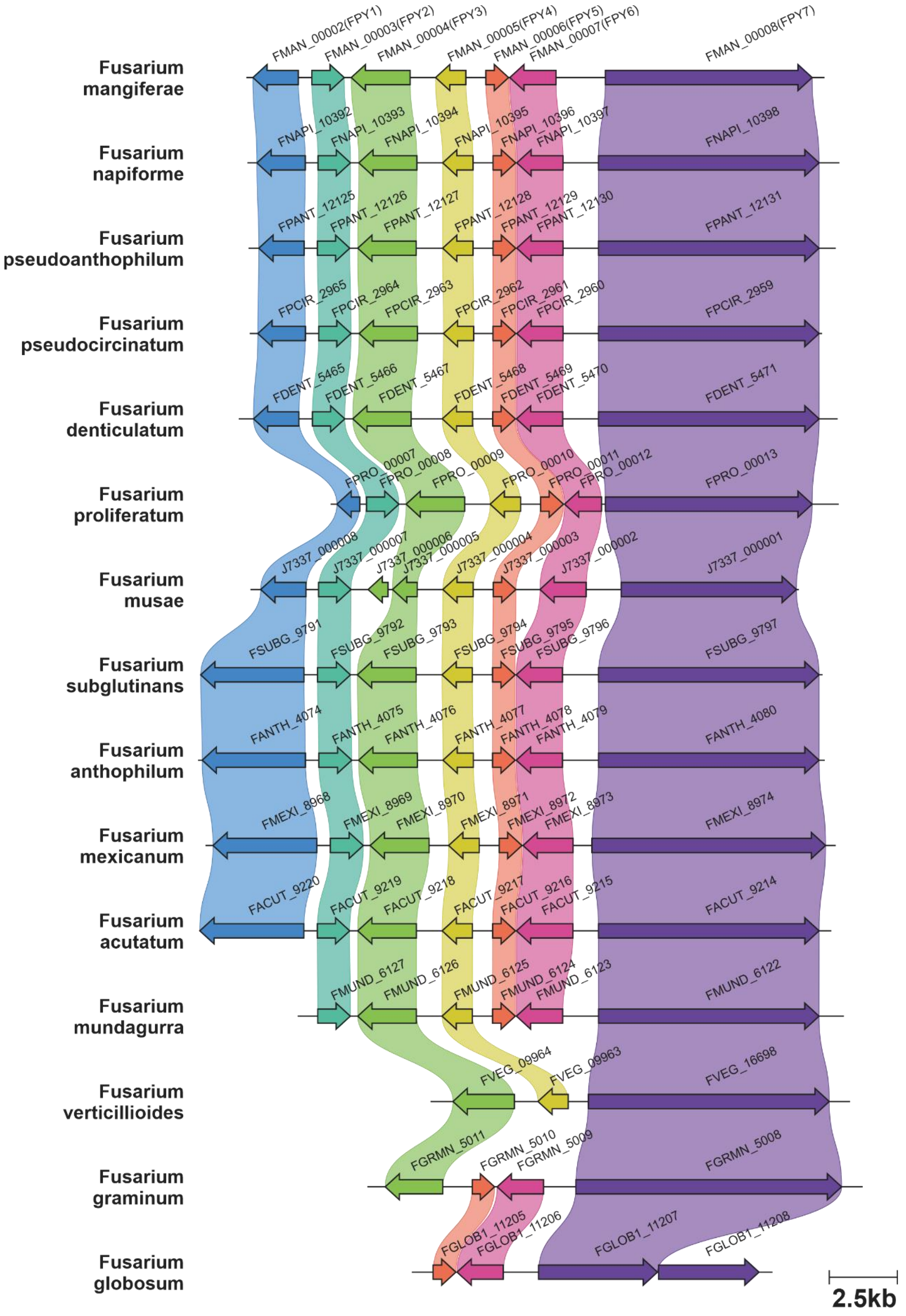

Figure S32: Comparison of *FPY* BGCs from different *Fusarium* species.

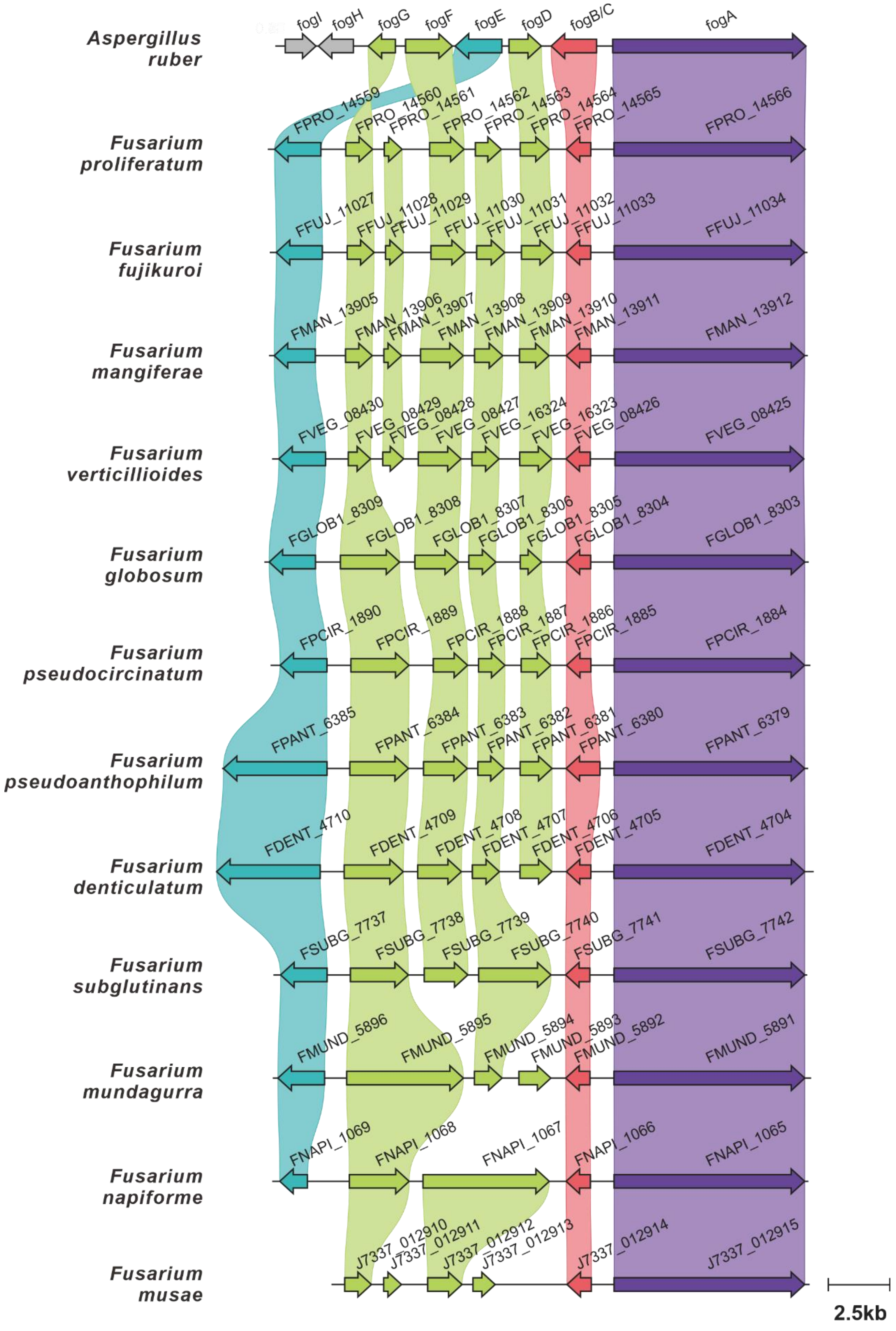

Figure S33: Comparison of *fog*BGCs from different *Fusarium* species.

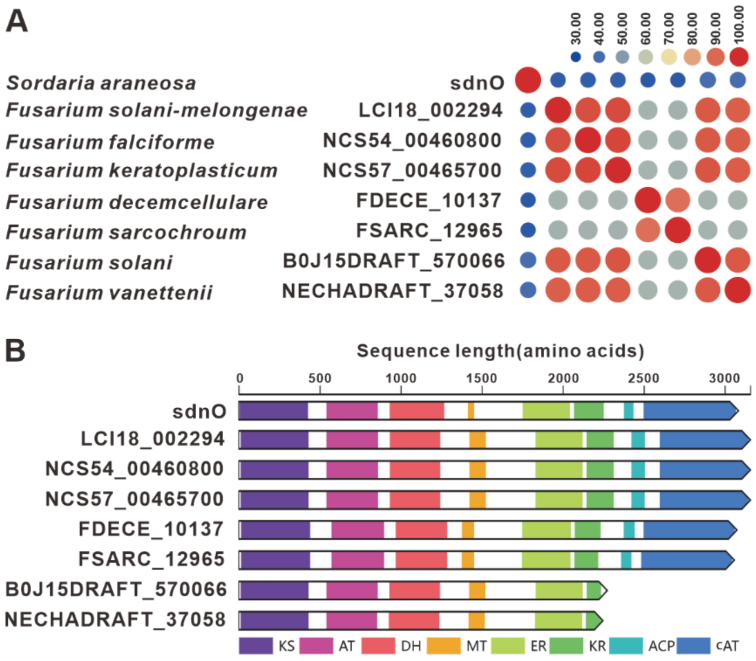

Figure S34: Comparison of the amino acid sequence identity of SdnO and its homologues (A), domain comparison of SdnO and its homologues (B).

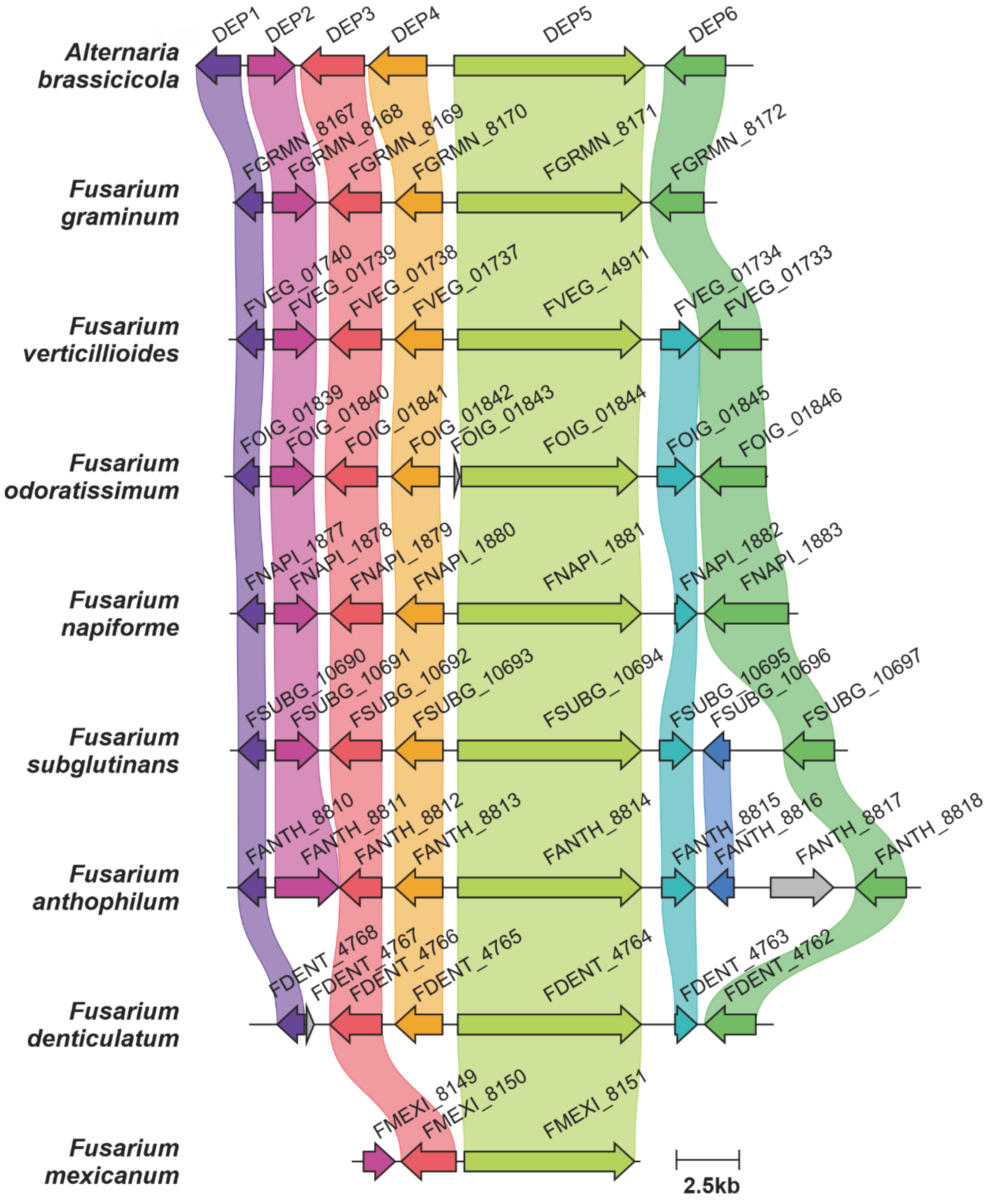

Figure S35: Comparison of *DEP*BGCs from different *Fusarium* species.

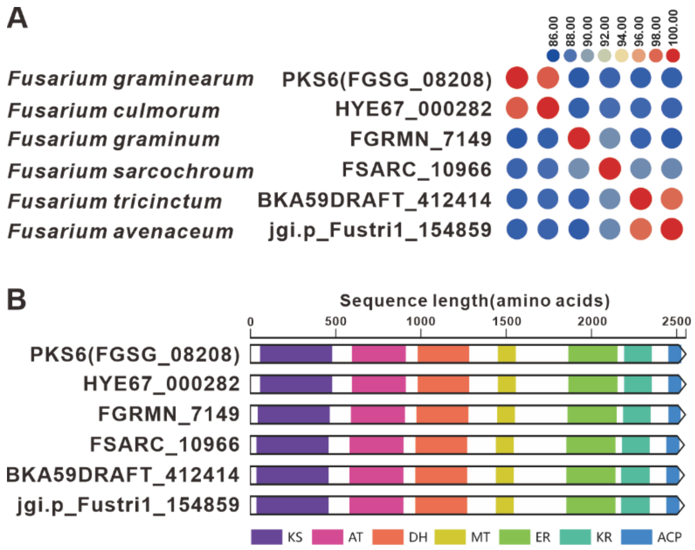

Figure S36: Comparison of the amino acid sequence identity of PKS6 and its homologues (A), domain comparison of PKS6 and its homologues (B).

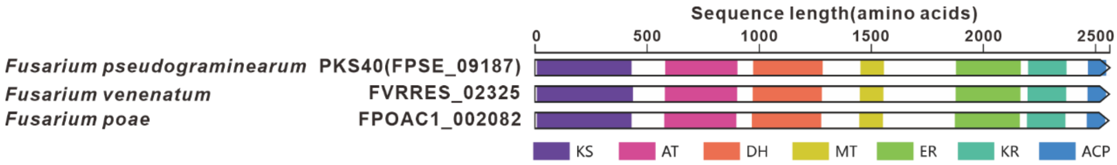

Figure S37: Domain comparison of PKS40 and its homologues.

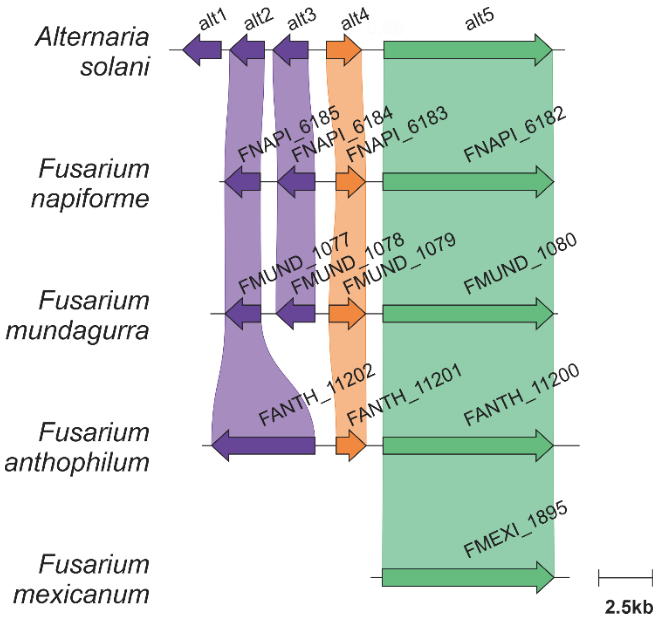

Figure S38: Comparison of *alt*BGCs from different *Fusarium* species.

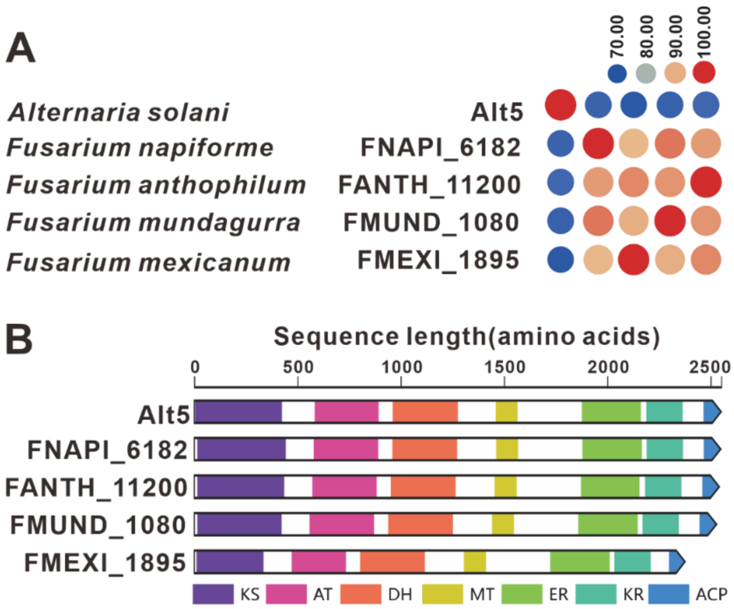

Figure S39: Comparison of the amino acid sequence identity of Alt5 and its homologues (A), domain comparison of Alt5 and its homologues (B).

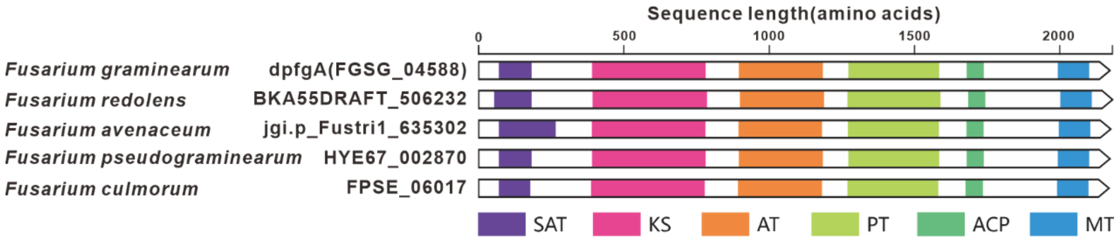

Figure S40: Domain comparison of DpfgA and its homologues.

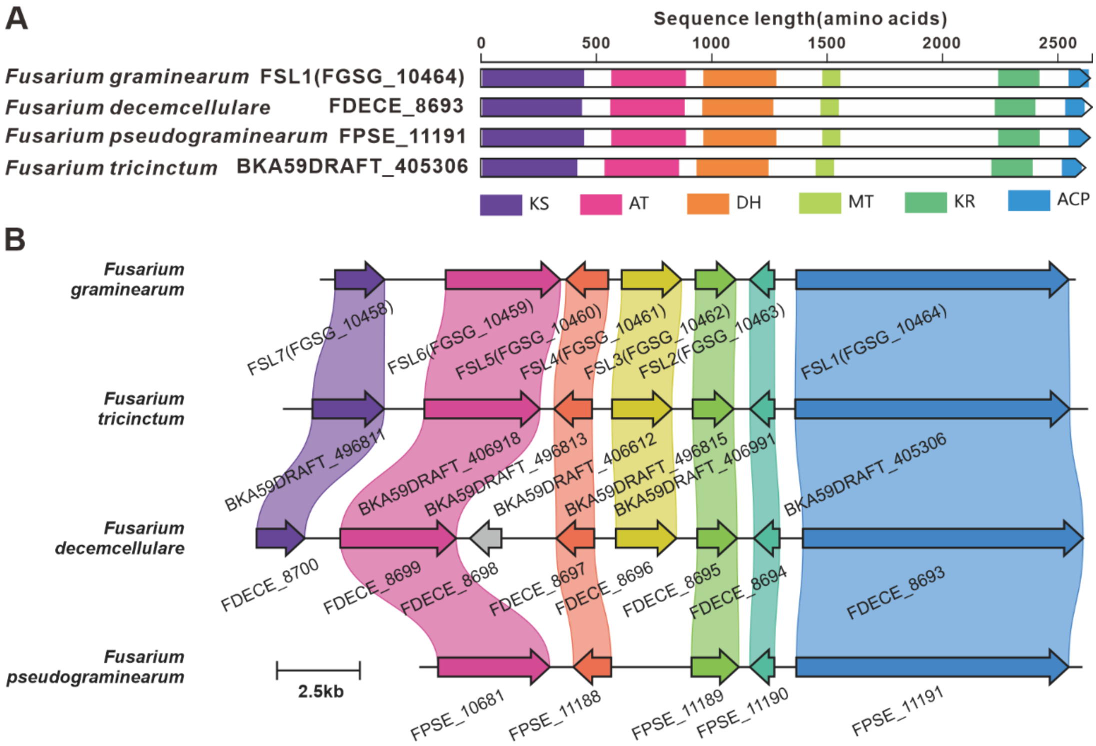

Figure S41: Domain comparison of FSL1 and its homologues (A), comparison of *FSL*BGCs from different *Fusarium* species (B).

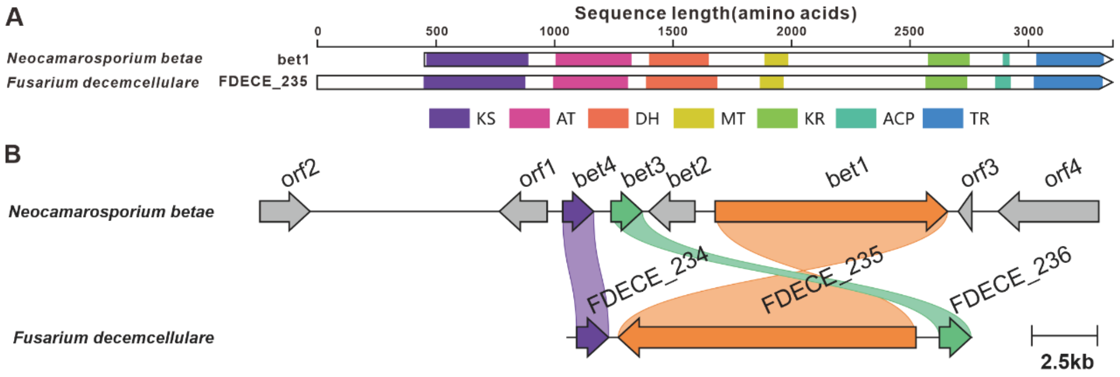

Figure S42: Domain comparison of Bet1 and its homologues (A), comparison of *bet*BGCs from different species (B).

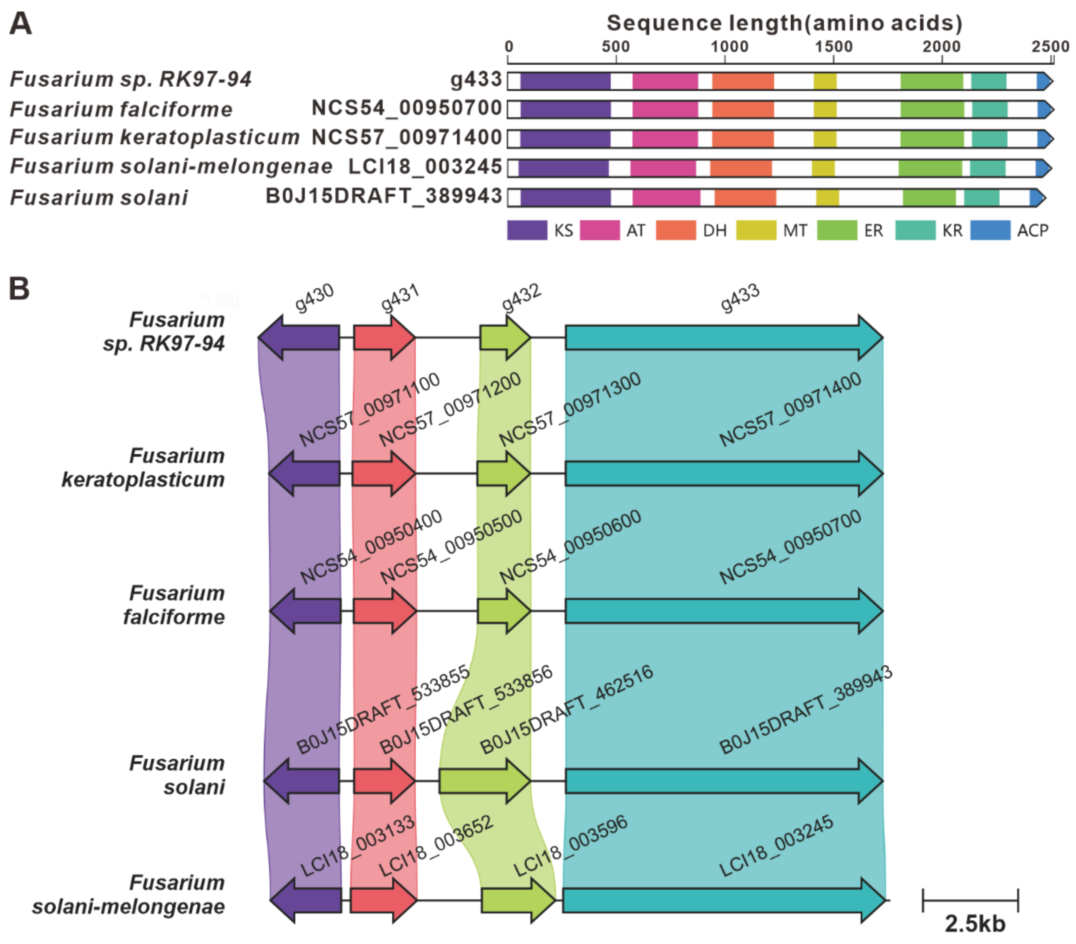

Figure S43: Domain comparison of G433 and its homologues (A), comparison of the BGC for G433 and its similar BGCs (B).

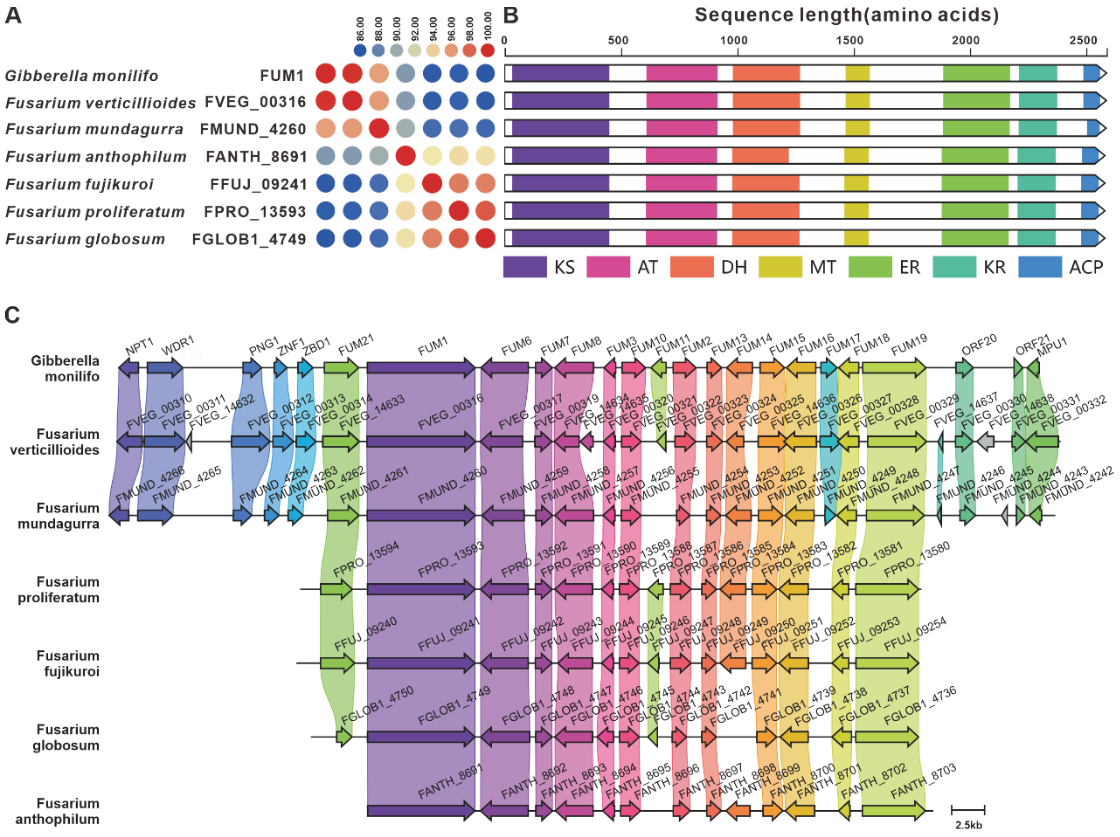

Figure S44: Comparison of the amino acid sequence identity of Alt5 and its homologues (A), domain comparison of FUM1 and its homologues (B), comparison of *FUM*BGCs from different *Fusarium* species (C).

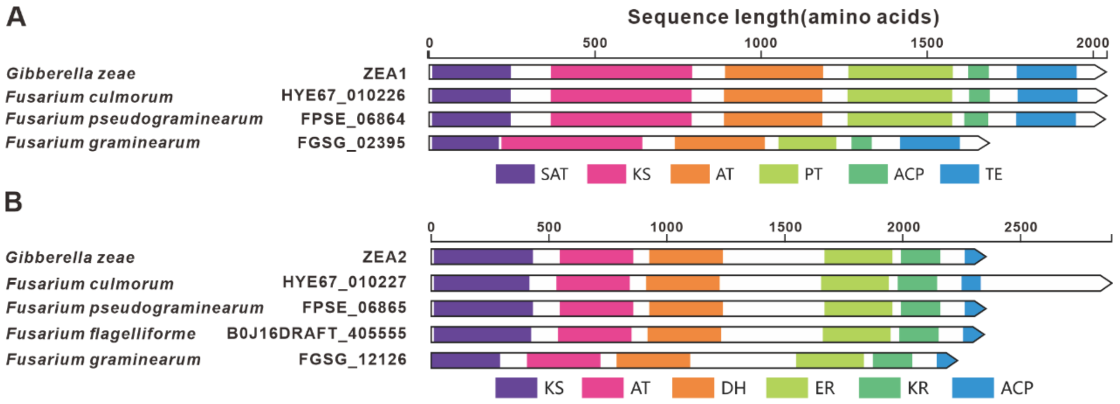

Figure S45: Domain comparison of ZEA1 and its homologues (A), domain comparison of ZEA2 and its homologues (B).

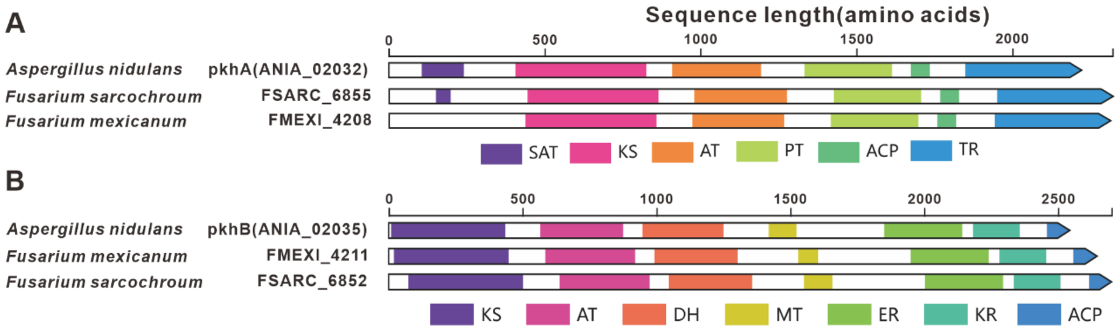

Figure S46: Domain comparison of PkhA and its homologues (A), domain comparison of PkhB and its homologues (B).

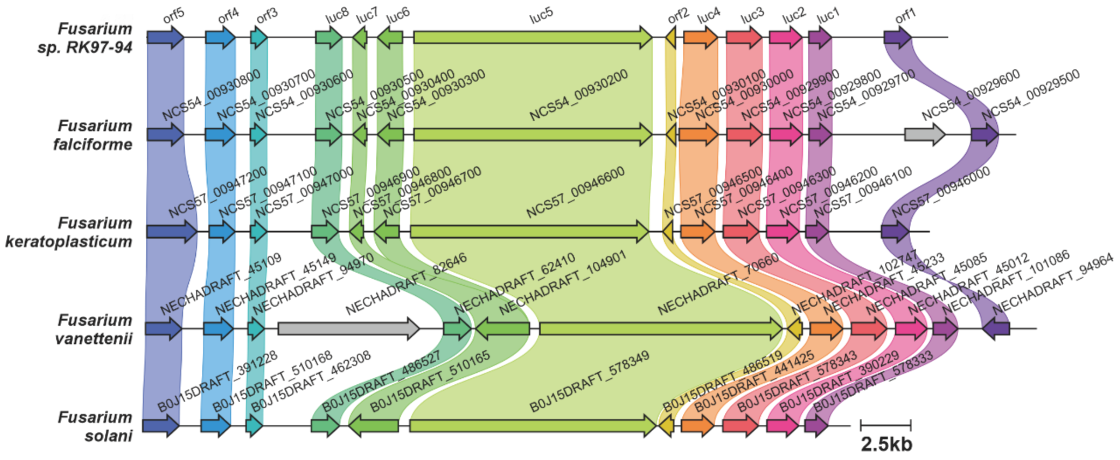

Figure S47: Comparison of *luc*BGCs from different *Fusarium* species.

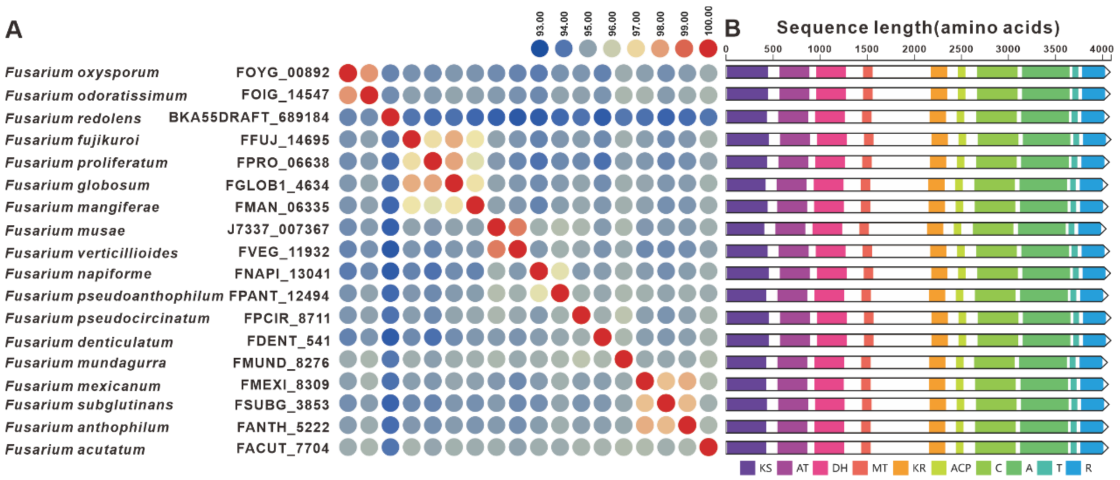

Figure S48: Amino acid sequence identity comparison (A) and domain comparison (B) of newly discoveried PKS-NRPSs in 35 pathogenic *Fusarium* species.

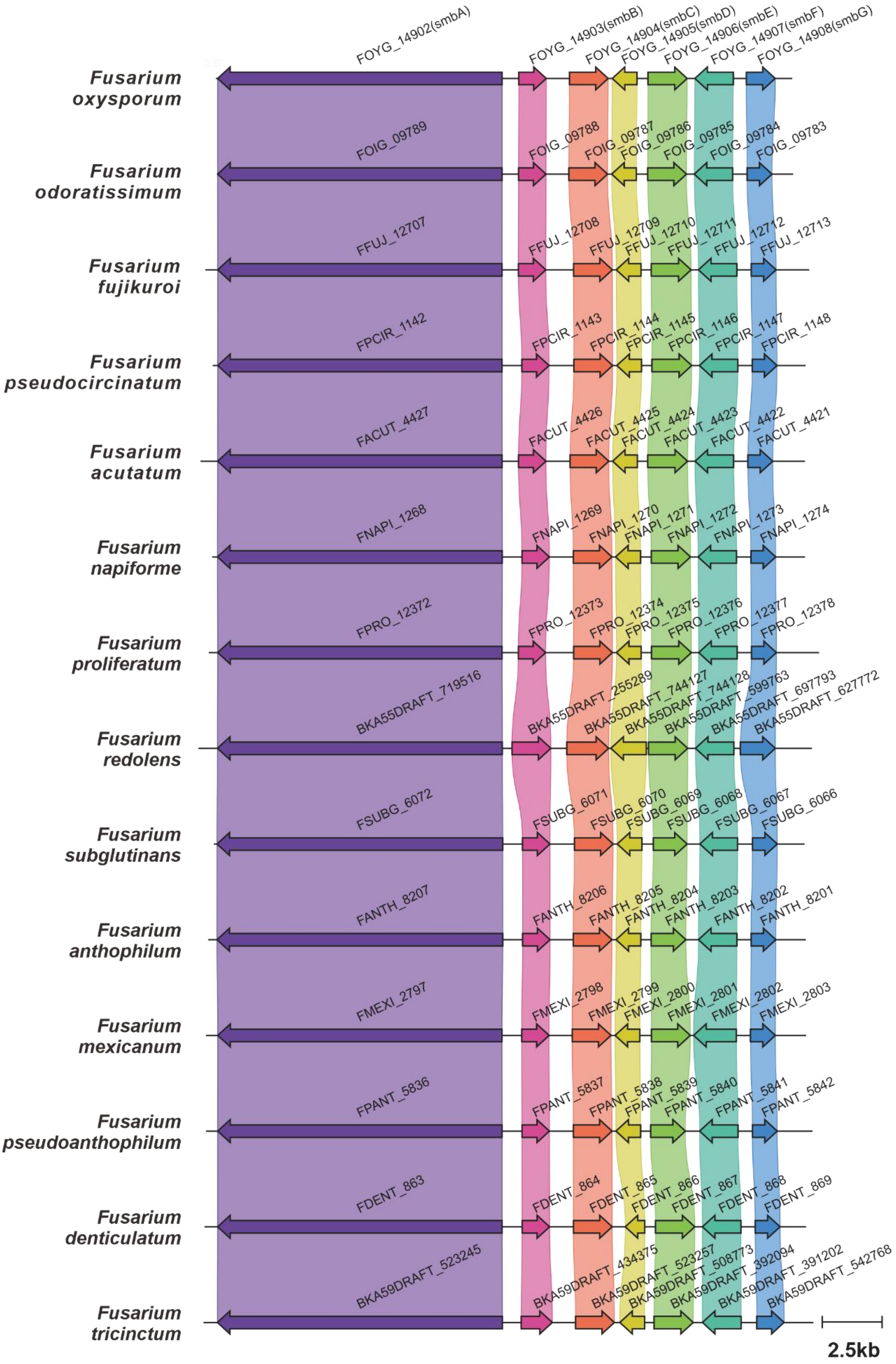

Figure S49: Comparison of *smb*BGCs from different *Fusarium* species.

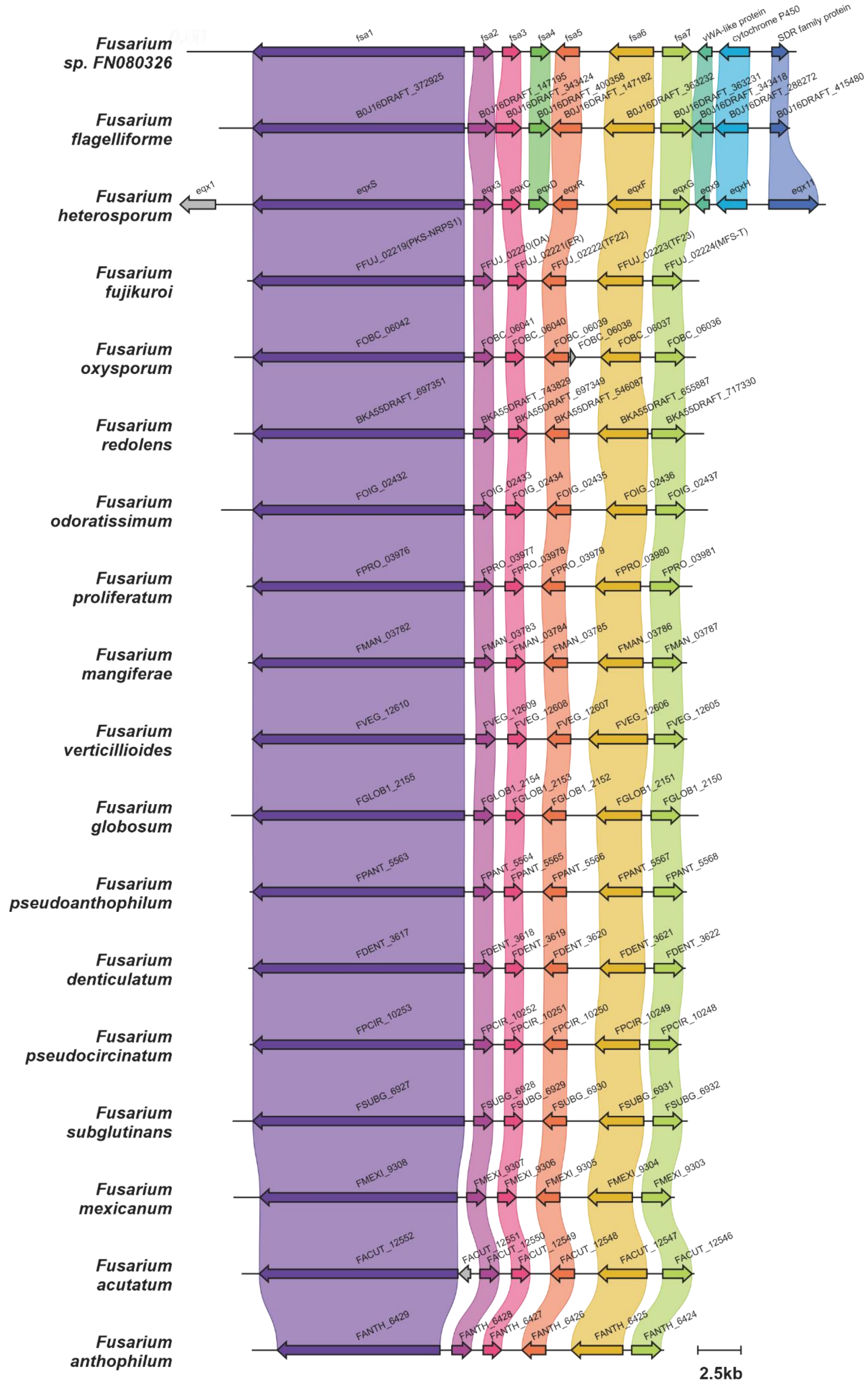

Figure S50: Comparison of *fsa*BGCs and *PKS-NRPS*BGCs from different *Fusarium* species.

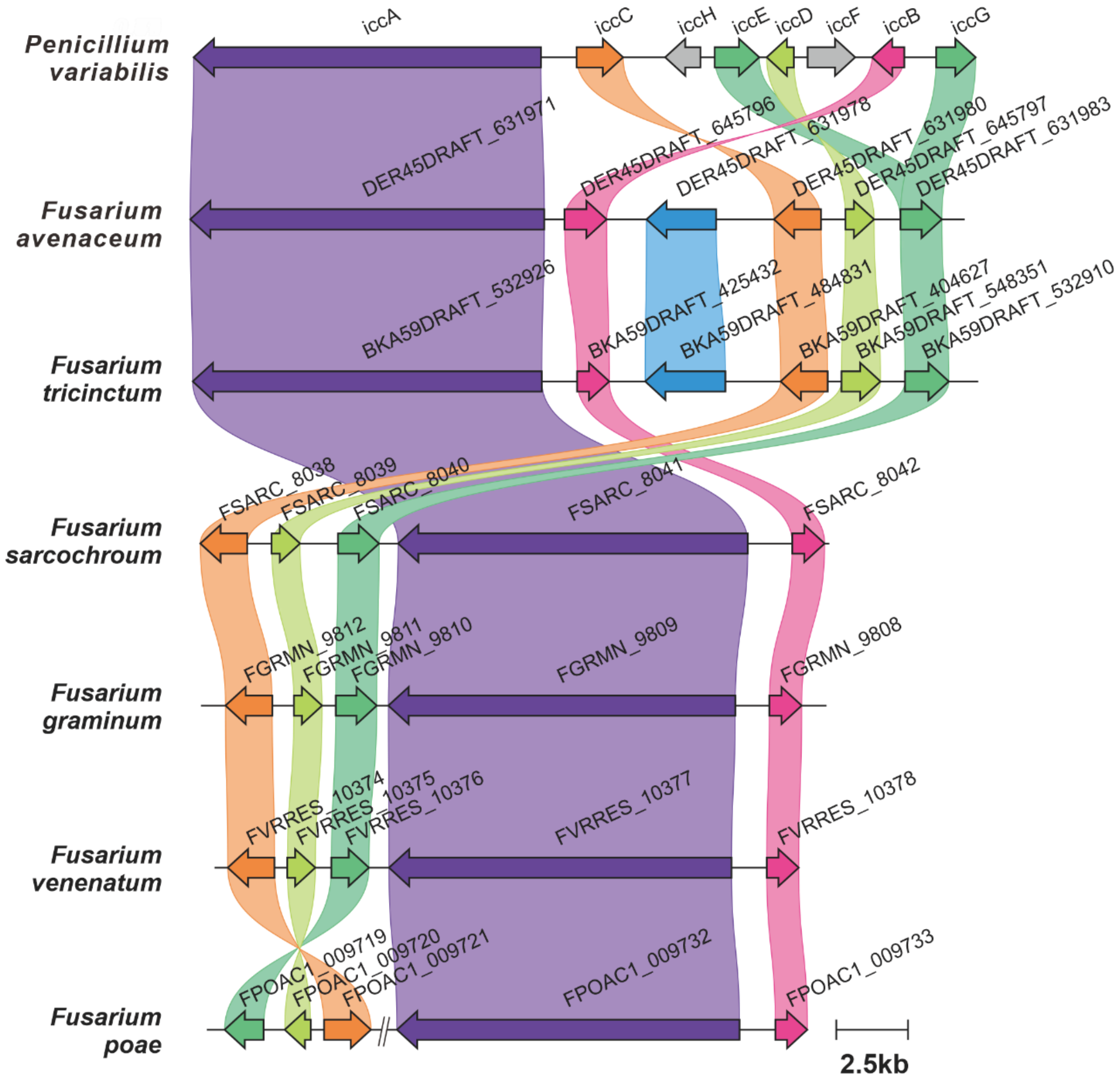

Figure S51: Comparison of *icc*BGCs from different *Fusarium* species.

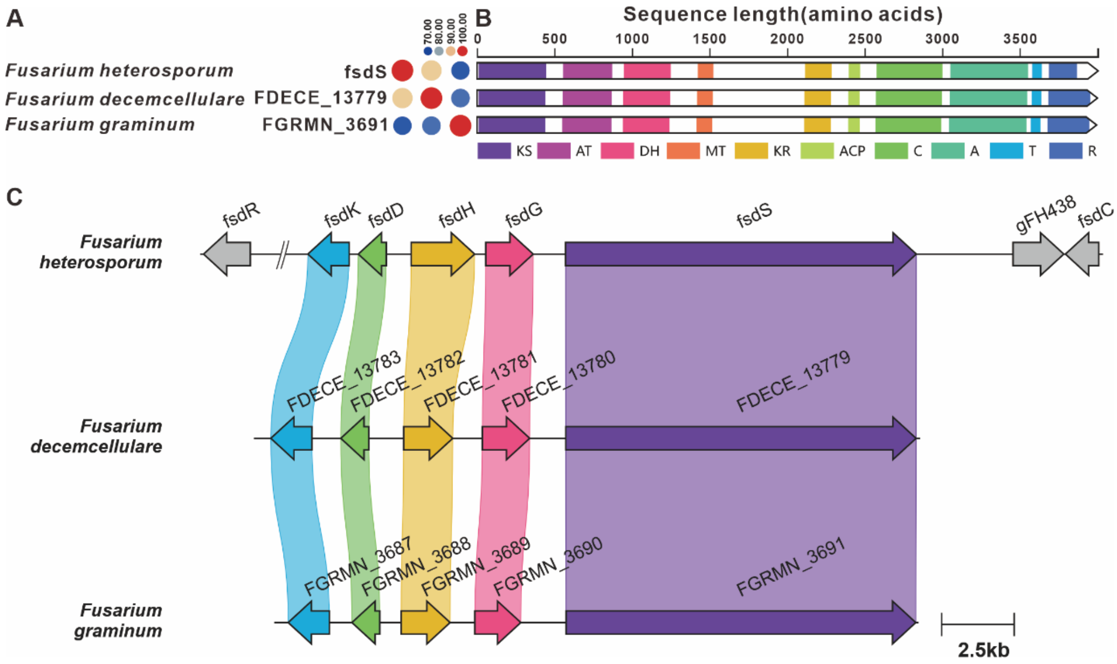

Figure S52: Comparison of the amino acid sequence identity of FsdS and its homologues (A), domain comparison of FsdS and its homologues (B), comparison of *fsd*BGCs from different *Fusarium* species (C).

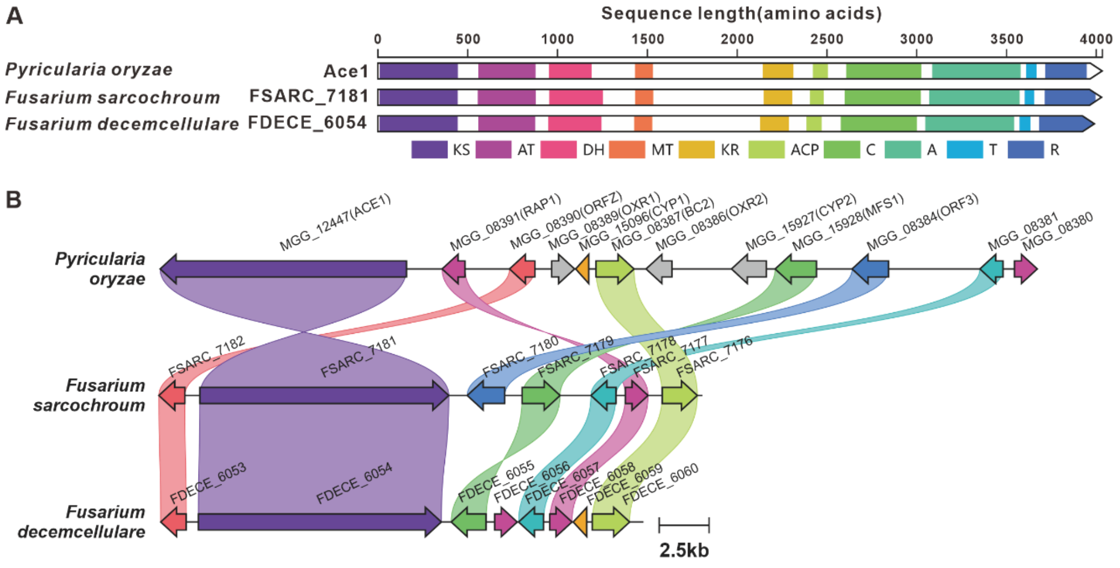

Figure S53: Domain comparison of ACE1 and its homologues (A), comparison of *ACE*BGCs from different species (B).

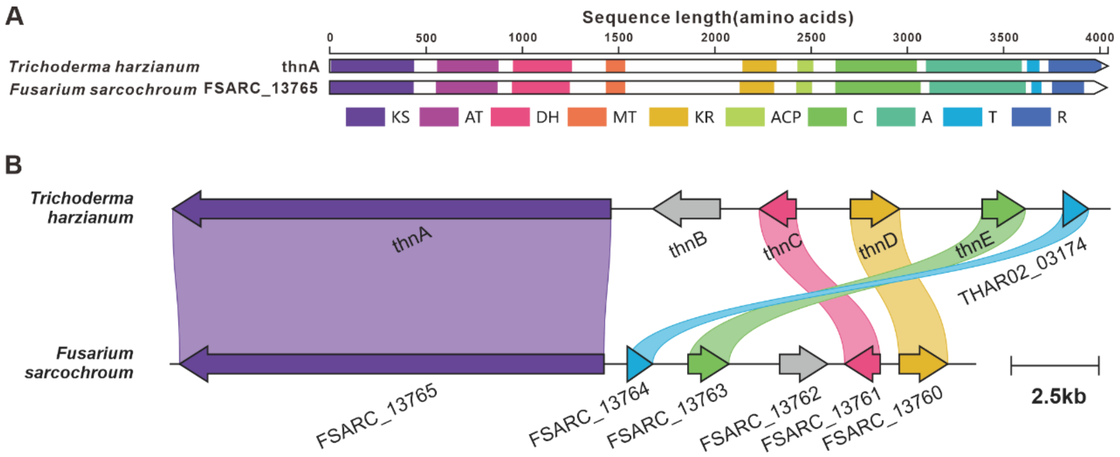

Figure S54: Domain comparison of ThnA and its homologues (A), comparison of *thn*BGCs from different species (B).

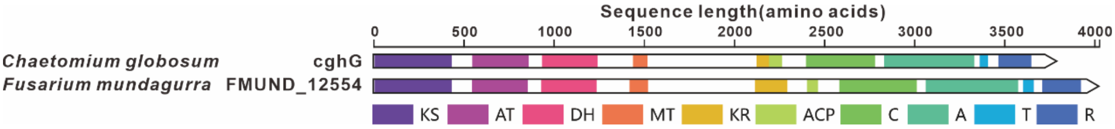

Figure S55: Domain comparison of CghG and its homologues.

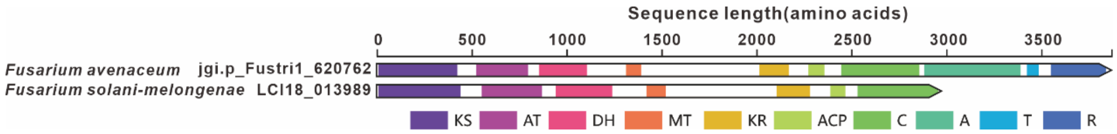

Figure S56: Domain comparison of LCI18_013989 and jgi.p_Fustri_620762.

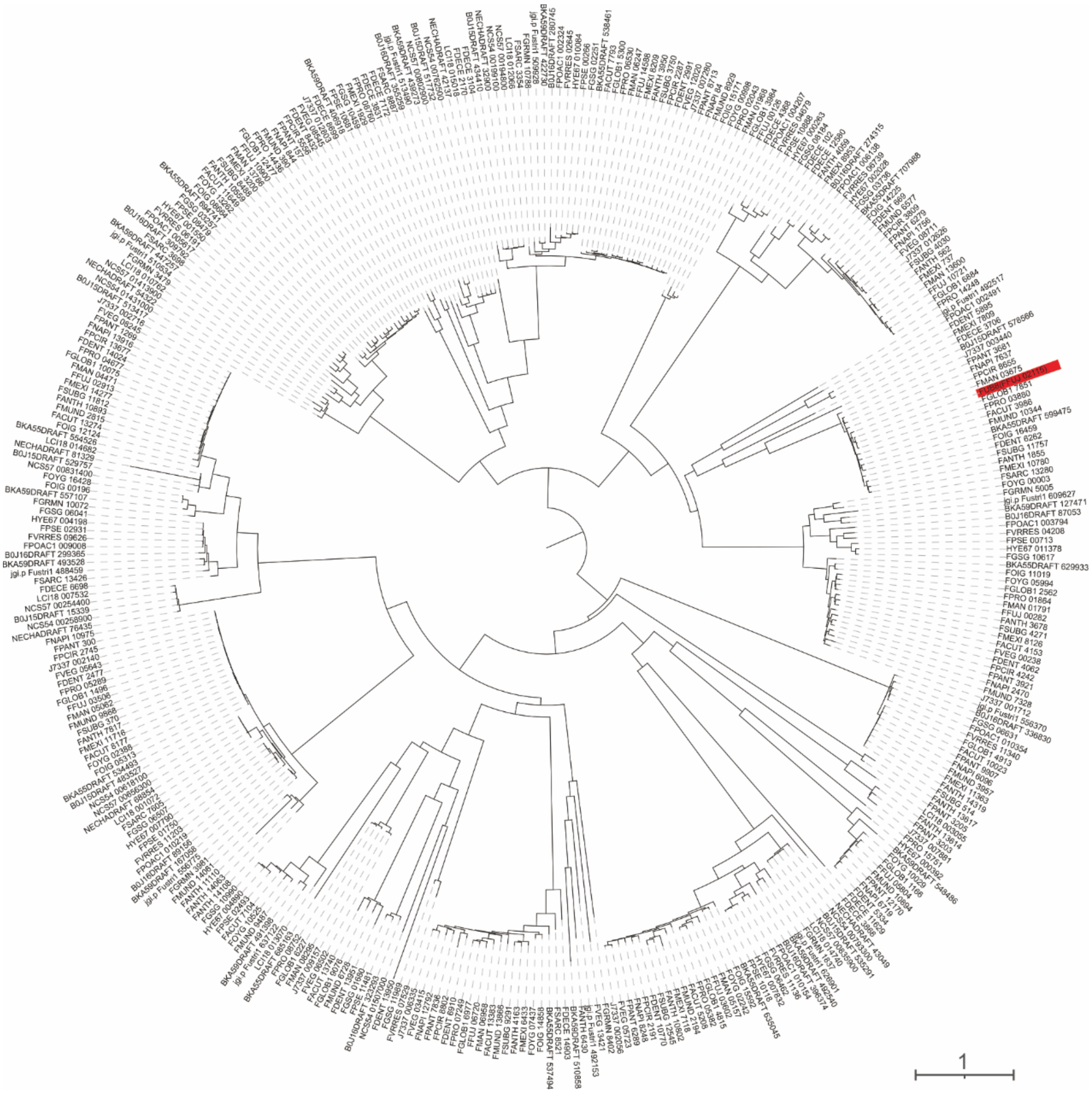

Figure S57: Cluster analysis of NRPS-like sequences based on phylogenetic tree.

Sequence with a red background indicate that the sequence has been characterised.

References.

1. Michielse, C.B. and M. Rep, *Pathogen profile update: Fusarium oxysporum.* MOL PLANT PATHOL, 2009. **10**(3): p. 311-324.
2. Matic, S., M.L. Gullino, and D. Spadaro, *The puzzle of bakanae disease through interactions between Fusarium fujikuroi and rice.* FRONT BIOSCI, 2017. **9**(2): p. 333-344.
3. Xie, S.Y., et al., *Whole-Genome Sequencing and Comparative Genome Analysis of Fusarium solani-melongenae Causing Fusarium Root and Stem Rot in Sweetpotatoes.* MICROBIOL SPECTR, 2022. **10**(4): p. e00683-22.
4. Debbarma, R., et al., *First report of root rot disease on Solanum lycopersicum L. caused by Fusarium vanettenii in India.* J PHYTOPATHOL, 2021. **169**(11-12): p. 752-756.
5. van Westerhoven, A.C., et al., *Dissemination of Fusarium wilt of banana in Mozambique caused by Fusarium odoratissimum Tropical Race 4.* PLANT DIS, 2023. **107**(ja): p. 628-632.
6. Farias, O.R., et al., *Occurrence of Fusarium proliferatum Causing Vascular Wilt on Cowpea (Vigna unguiculata) in Brazil.* PLANT DIS, 2022. **106**: p. 1992.
7. Drakopoulos, D., et al., *Control of Fusarium graminearum in Wheat With Mustard-Based Botanicals: From in vitro to in planta.* FRONT MICROBIOL, 2020. **11**: p. 1595.
8. Xie, Y., et al., *A Megabirnavirus Alleviates the Pathogenicity of Fusarium pseudograminearum to Wheat.* PHYTOPATHOLOGY, 2022. **112**(5): p. 1175-1184.
9. Atanasoff-Kardjalieff, A.K., et al., *Biosynthesis of Fusapyrone Depends on the H3K9 Methyltransferase, FmKmt1, in Fusarium mangiferae.* FRONT FUNGAL BIOL, 2021. **2**.
10. Scherm, B., et al., *Fusarium culmorum: causal agent of foot and root rot and head blight on wheat.* MOL PLANT PATHOL, 2013. **14**(4): p. 323-341.
11. Wang, R.-Y., et al., *First Report of Fusarium solani Causing Fusarium Root Rot and Stem Canker on Storage Roots of Sweet Potato in China.* PLANT DIS, 2014. **98**(1): p. 160-160.
12. Fan, S., et al., *American Ginseng Root Rot Caused by Fusarium redolens in China.* PLANT DIS, 2021. **105**: p. 2734.
13. Xu, X., et al., *Fusarium Species Associated with Maize Leaf Blight in Heilongjiang Province, China.* J FUNGI, 2022. **8**(11): p. 1170.
14. Degradi, L., et al., *Exploring Mitogenomes Diversity of Fusarium musae from Banana Fruits and Human Patients.* MICROORGANISMS, 2022. **10**(6): p. 1115.
15. Wang, C.G., et al., *First Report of Fusarium falciforme Causing Leaf Blight in Acacia mangium in China.* PLANT DIS, 2022. **0**(0): p. PDIS-07-22-1583-PDN.
16. Rigorth, K.S., M.R. Finckh, and A. Šišić, *First Report of Fusarium venenatum Causing Foot and Root Rot of Wheat (Triticum aestivum) in Germany.* PLANT DIS, 2021. **105**(6): p. 1855.
17. Wang, Y., R. Wang, and Y. Sha, *Distribution, pathogenicity and disease control of Fusarium tricinctum.* FRONT MICROBIOL, 2022. **13**: p. 939927.
18. Ghimire, B., et al., *First Report of Fusarium poae Causing Fusarium Head Blight of Wheat in Georgia, U.S.A.* PLANT DIS, 2021. **105**(2): p. 491-491.
19. Chiewchanvit, S., et al., *Melanization of Fusarium keratoplasticum (F. solani Species Complex) During Disseminated Fusariosis in a Patient with Acute Leukemia.* MYCOPATHOLOGIA, 2017. **182**(9-10): p. 879-885.
20. Chen, Y., H.C. Kistler, and Z. Ma, *Fusarium graminearum Trichothecene Mycotoxins: Biosynthesis, Regulation, and Management.* ANNU REV PHYTOPATHOL, 2019. **57**: p. 15-39.
21. de Souza, M., et al., *Fusarium napiforme systemic infection: case report with molecular characterization and antifungal susceptibility tests.* SPRINGERPLUS, 2014. **3**: p. 492.
22. Santillán-Mendoza, R., et al., *Genetic diversity of Fusarium mexicanum, causal agent of mango and big-leaf mahogany malformation in Mexico.* MOL BIOL REP, 2019. **46**(4): p. 3887-3897.
23. Serrato-Diaz, L.M., et al., *First Report of Fusarium decemcellulare Causing Inflorescence Wilt*

and Vascular and Flower Necrosis of Rambutan (Nephelium lappaceum), Longan (Dimocarpus longan), and Mango (Mangifera indica). PLANT DIS, 2015. **99**(8): p. 1187.
24. Gopalakrishnan, S., et al., *Chickpea wilt: identification and toxicity of 8-O-methyl-fusarubin from Fusarium acutatum.* PHYTOCHEMISTRY, 2005. **66**(13): p. 1536-1539.
25. Ferrigo, D., et al., *Influence of H(2)O(2)-Induced Oxidative Stress on In Vitro Growth and Moniliformin and Fumonisins Accumulation by Fusarium proliferatum and Fusarium subglutinans.* TOXINS, 2021. **13**(9): p. 653.
26. Blacutt, A.A., et al., *Fusarium verticillioides: Advancements in Understanding the Toxicity, Virulence, and Niche Adaptations of a Model Mycotoxigenic Pathogen of Maize.* PHYTOPATHOLOGY, 2018. **108**(3): p. 312-326.
27. Mesny, F., et al., *Genetic determinants of endophytism in the Arabidopsis root mycobiome.* NAT COMMUN, 2021. **12**(1): p. 7227.
28. Gagkaeva, T.Y., O.P. Gavrilova, and A.S. Orina, *First Report of Fusarium globosum Associated with Barley Grain in the Southwestern Part of Siberia.* PLANT DIS, 2019. **103**(3): p. 588-588.
29. Ono, L.T., et al., *Fungal communities in Brazilian cassava tubers and food products.* INT J FOOD MICROBIOL, 2023. **384**: p. 109909.
30. Freeman, S., et al., *First Report of Mango Malformation Disease Caused by Fusarium pseudocircinatum in Mexico.* PLANT DIS, 2014. **98**(11): p. 1583.
31. González, M.S., et al., *First Report of Fusarium denticulatum from Sweet Potato in Venezuela.* PLANT DIS, 2003. **87**(2): p. 202.
32. Amobonye, A., et al., *Characterisation, pathogenicity and hydrolytic enzyme profiling of selected Fusarium species and their inhibition by novel coumarins.* ARCH MICROBIOL, 2021. **203**(6): p. 3495-3508.
33. Nicolli, C.P., et al., *Fusarium fujikuroi species complex in Brazilian rice: Unveiling increased phylogenetic diversity and toxigenic potential.* INT J FOOD MICROBIOL, 2020. **330**: p. 108667.
34. Sandoval-Denis, M., et al., *Symptomatic Citrus trees reveal a new pathogenic lineage in Fusarium and two new Neocosmospora species.* PERSOONIA, 2018. **40**: p. 1-25.
35. Nelson, P.E., *Taxonomy and biology of Fusarium moniliforme.* MYCOPATHOLOGIA, 1992. **117**(1-2): p. 29-36.
36. Studt, L., et al., *Biosynthesis of fusarubins accounts for pigmentation of Fusarium fujikuroi perithecia.* APPL ENVIRON MICROBIOL, 2012. **78**(12): p. 4468-4480.
37. Son, S.W., et al., *Bikaverin and fusaric acid from Fusarium oxysporum show antioomycete activity against Phytophthora infestans.* J Appl Microbiol, 2008. **104**(3): p. 692-698.
38. von Bargen, K.W., et al., *Isolation and Structure Elucidation of Fujikurins A-D: Products of the PKS19 Gene Cluster in Fusarium fujikuroi.* J NAT PROD, 2015. **78**(8): p. 1809-1815.
39. Miyamoto, Y., et al., *ACTTS3 Encoding a Polyketide Synthase Is Essential for the Biosynthesis of ACT-Toxin and Pathogenicity in the Tangerine Pathotype of Alternaria alternata.* MOL PLANT MICROBE IN, 2010. **23**(4): p. 406-414.
40. Wight, W.D., et al., *Biosynthesis and Role in Virulence of the Histone Deacetylase Inhibitor Depudecin from Alternaria brassicicola.* Molecular Plant-Microbe Interactions®, 2009. **22**(10): p. 1258-1267.
41. Li, H., et al., *Chemical Ecogenomics-Guided Discovery of Phytotoxic alpha-Pyrones from the Fungal Wheat Pathogen Parastagonospora nodorum.* ORG LETT, 2018. **20**(19): p. 6148-6152.
42. Fujii, I., et al., *An iterative type I polyketide synthase PKSN catalyzes synthesis of the decaketide alternapyrone with regio-specific octa-methylation.* CHEM BIOL, 2005. **12**(12): p. 1301-1309.
43. Bonsch, B., et al., *Identification of genes encoding squalestatin S1 biosynthesis and in vitro production of new squalestatin analogues.* CHEM COMMUN, 2016. **52**(41): p. 6777-6780.
44. Brock, N.L., et al., *Genetic dissection of sesquiterpene biosynthesis by Fusarium fujikuroi.*

CHEMBIOCHEM, 2013. **14**(3): p. 311-315.
45. Hwang, I.S. and I.P. Ahn, *Multi-Homologous Recombination-Based Gene Manipulation in the Rice Pathogen Fusarium fujikuroi.* Plant Pathol J, 2016. **32**(3): p. 173-181.
46. Kimura, M., et al., *The trichothecene biosynthesis gene cluster of Fusarium graminearum F15 contains a limited number of essential pathway genes and expressed non-essential genes.* FEBS LETT, 2003. **539**(1-3): p. 105-110.
47. Lee, T., et al., *Tri13 and Tri7 determine deoxynivalenol- and nivalenol-producing chemotypes of Gibberella zeae.* APPL ENVIRON MICROBIOL, 2002. **68**(5): p. 2148-2154.
48. Tokai, T., et al., *Screening of putative oxygenase genes in the Fusarium graminearum genome sequence database for their role in trichothecene biosynthesis.* FEMS Microbiol Lett, 2005. **251**(2): p. 193-201.
49. Wollenberg, R.D., et al., *Chrysogine Biosynthesis Is Mediated by a Two-Module Nonribosomal Peptide Synthetase.* J NAT PROD, 2017. **80**(7): p. 2131-2135.
50. Romans-Fuertes, P., et al., *Identification of the non-ribosomal peptide synthetase responsible for biosynthesis of the potential anti-cancer drug sansalvamide in Fusarium solani.* CURR GENET, 2016. **62**(4): p. 799-807.
51. Slightom, J.L., et al., *Cloning and molecular characterization of the gene encoding the Aureobasidin A biosynthesis complex in Aureobasidium pullulans BP-1938.* GENE, 2009. **431**(1-2): p. 67-79.
52. Yoshimi, A., et al., *Heterologous Production of a Novel Cyclic Peptide Compound, KK-1, in Aspergillus oryzae.* Front Microbiol, 2018. **9**: p. 690.
53. Jin, J.M., et al., *Functional characterization and manipulation of the apicidin biosynthetic pathway in Fusarium semitectum.* MOL MICROBIOL, 2010. **76**(2): p. 456-466.
54. Droce, A., et al., *Functional Analysis of the Fusarielin Biosynthetic Gene Cluster.* MOLECULES, 2016. **21**(12): p. 1701.
55. Kim, Y.T., et al., *Two different polyketide synthase genes are required for synthesis of zearalenone in Gibberella zeae.* MOL MICROBIOL, 2005. **58**(4): p. 1102-1113.
56. Yin, W.B., et al., *A nonribosomal peptide synthetase-derived iron(III) complex from the pathogenic fungus Aspergillus fumigatus.* J Am Chem Soc, 2013. **135**(6): p. 2064-2067.
57. Kakule, T.B., et al., *Two related pyrrolidinedione synthetase loci in Fusarium heterosporum ATCC 74349 produce divergent metabolites.* ACS Chem Biol, 2013. **8**(7): p. 1549-1557.
